# A New Regression Model for the Analysis of Overdispersed and Zero-Modified Count Data

**DOI:** 10.3390/e23060646

**Published:** 2021-05-21

**Authors:** Wesley Bertoli, Katiane S. Conceição, Marinho G. Andrade, Francisco Louzada

**Affiliations:** 1 Department of Statistics, Federal University of Technology, Paraná, Av. Sete de Setembro, 3165 Rebouças, Curitiba 80230-901, PR, Brazil; 2 Department of Applied Mathematics and Statistics, Institute of Mathematical and Computer Sciences, University of São Paulo, Av. Trab. São Carlense, 400 Parque Arnold Schimidt, São Carlos 13566-590, SP, Brazil; katiane@icmc.usp.br (K.S.C.); marinho@icmc.usp.br (M.G.A.); louzada@icmc.usp.br (F.L.)

**Keywords:** Bayesian inference, hurdle model, Monte Carlo simulation, overdispersion, Poisson–Sujatha distribution, zero-modified data, 02.50.-r, 62E15, 62J20, 62F15

## Abstract

Count datasets are traditionally analyzed using the ordinary Poisson distribution. However, said model has its applicability limited, as it can be somewhat restrictive to handling specific data structures. In this case, the need arises for obtaining alternative models that accommodate, for example, overdispersion and zero modification (inflation/deflation at the frequency of zeros). In practical terms, these are the most prevalent structures ruling the nature of discrete phenomena nowadays. Hence, this paper’s primary goal was to jointly address these issues by deriving a fixed-effects regression model based on the hurdle version of the Poisson–Sujatha distribution. In this framework, the zero modification is incorporated by considering that a binary probability model determines which outcomes are zero-valued, and a zero-truncated process is responsible for generating positive observations. Posterior inferences for the model parameters were obtained from a fully Bayesian approach based on the g-prior method. Intensive Monte Carlo simulation studies were performed to assess the Bayesian estimators’ empirical properties, and the obtained results have been discussed. The proposed model was considered for analyzing a real dataset, and its competitiveness regarding some well-established fixed-effects models for count data was evaluated. A sensitivity analysis to detect observations that may impact parameter estimates was performed based on standard divergence measures. The Bayesian *p*-value and the randomized quantile residuals were considered for the task of model validation.

## 1. Introduction

The ordinary Poisson (P) distribution is often adopted for the analysis of count data, mainly due to its simplicity and having computational implementations available for most of the standard statistical packages. However, it is well-known that such a model is not suitable to describe over/underdispersed counts. Apart from data transformation, the most popular approach to circumvent such an issue is based on using hierarchical models that can accommodate different overdispersion levels [1].

The negative binomial (NB) distribution (that may arise as a P mixture model by using a gamma distribution for the continuous part) is undoubtedly the most popular alternative to model extra-P variability. There is extensive literature regarding other discrete mixed distributions that can accommodate different levels of overdispersion, for example, the Poisson–Lindley [2], the Poisson–lognormal [3], the Poisson–inverse Gaussian [4], the negative binomial–Lindley [5], the Poisson–Janardan [6], the two-parameter Poisson–Lindley [7], the Poisson–Amarendra [8], the Poisson–Shanker [9], the Poisson–Sujatha (PS) [10], the quasi-Poisson–Lindley [11], the weighted negative binomial–Lindley [12] the Poisson-weighted Lindley [13], the binomial-discrete Lindley [14], and the two-parameter Poisson–Sujatha [15], among many others.

Unfortunately, there is a significant drawback regarding such mixture models: they do not fit well when data present a modification in the frequency of zeros (typically underestimates the data dispersion and the frequency of zero-valued outcomes). The most common case in practice is the presence of an excessive number of zero-valued observations and a skewed distribution of positive values. In this way, developing P-based two-part models (zero-inflated/hurdle models) became necessary. Prominent works addressing this task are [16,17,18,19,20,21,22].

Several authors have considered these approaches to analyze real data, and here we point out a few. Ref. [23] have sought to deal with the excess of zeros on data from recreational trips. Ref. [24] have shown that the modeling of migration frequency data can be improved using zero-inflated Poisson models. Ref. [25] have exploited the apple shoot propagation data, and they have addressed the modeling task by using several zero-inflated regression models. In the social sciences, ref. [26] have considered the hurdle version of the P model for the number of homicides in Chicago (State of Illinois, US). Ref. [27] provided an application to private health insurance count data using ordinary and zero-inflated Poisson regression models. Further applications of these models were considered in quantitative studies about HIV-risk reduction [28,29], for the modeling of some occupational allergic diseases in France [30], for the analysis of DNA sequencing data [31], and for the modeling of several datasets on chromosomal aberrations induced by radiation [32]. A Bayesian approach for the zero-inflated Poisson (ZIP) distribution was considered by [33], and by [34] in a regression framework with fixed-effects.

Noticeably, most developed works are focused on the modeling of zero inflation, but zero-deflated data are also frequently observed in practice. However, there are still very few studies addressing this case [35], but this situation is often referred to in works handling zero inflation. In this context, a more comprehensive approach is provided by zero-modified models, which are flexible tools to handle count data with inflation/deflation at zero when there is no information about the nature of such a phenomenon.

Some of the most relevant works about zero-modified and hurdle models are cited in the following. Ref. [35] have introduced the zero-modified Poisson (ZMP) regression model, and ref. [36] have considered such a model as an alternative for the analysis of Brazilian leptospirosis notification data. The possible loss due to the specification of a ZMP model for analyzing samples without zero modification was studied by [37] using the Kullback–Leibler divergence. The hurdle version of the power series distribution was presented and well discussed by [38], and ref. [39] have adopted a Bayesian approach for the zero-modified Poisson model to predict match outcomes of the Spanish La Liga (2012–2013). Besides, ref. [40] have proposed the zero-modified Poisson–Shanker regression model, whose usefulness was illustrated through its application to fetal death notification data, and ref. [41] have introduced the zero-modified Poisson–Lindley regression model with fixed-effects under a fully Bayesian approach.

Accordingly, this paper aims to extend the works of [42,43] in the sense of developing a new fixed-effects regression model for count data based on the zero-modified Poisson–Sujatha distribution (ZMPS). Ref. [42] have introduced and exploited the theoretical ZMPS distribution’s main statistical properties. On the other hand, ref. [43] have proposed a new class of zero-modified models, whose baseline distributions are Poisson mixtures, including the PS. The present paper also extends the works of [40,41] since the ZMPS model differentiates from the zero-modified Poisson–Lindley and Poisson–Shanker by the ability, for example, to describe better (by adjusting its shape parameter) those discrete phenomena in which the probabilities of observing 0 s and 1 s are low (see [43], Figure 2).

Formally, a discrete random variable *Y* defined into $N_{0}=\left\{0,1,\ldots \right\}$ is said to follow a ZMPS distribution if its probability mass function (pmf) can be written as

(1) $$P_{*}\;\left(Y=y;\mu ,p\right)=\left(1-p\right)\delta_{y}+p\;P\left(Y=y;\mu \right),\;\;\;\;y\in N_{0},$$

where *p* is the zero-modification parameter and $\delta_{y}$ is an indicator function, so that $\delta_{y}=1$ if y=0 and $\delta_{y}=0$ otherwise. Additionally, $\mu \in R_{+}$ is the expected value of the ordinary PS distribution, whose reparameterized pmf is given by

$$\begin{array}{ccc} P\;\left(Y=y;\mu \right) & = & \frac{h^{3}\left(\mu \right)}{h^{2}\left(\mu \right)+h\left(\mu \right)+2}\\ & & \left[\frac{y^{2}+y\left[h\left(\mu \right)+4\right]+\left[h^{2}\left(\mu \right)+3h\left(\mu \right)+4\right]}{{\left[h\left(\mu \right)+1\right]}^{y+3}}\right],\;\;\;\;y\in N_{0}, \end{array}$$

where

(2) $$h\left(\mu \right)=\frac{1}{3\mu }\left[\left(s\left(\mu \right)-\mu +1\right)-\frac{\left(\mu -1\right)\left(5\mu +1\right)}{s\left(\mu \right)}\right],$$

with

$$s\left(\mu \right)={\left[3\mu \sqrt{21\mu^{4}+84\mu^{3}+513\mu^{2}+96\mu +15}+2\mu \left(4\mu^{2}+33\mu +3\right)+1\right]}^{1\;/\;3},$$

and $\mu =\left(\theta^{2}+2\theta +6\right){\left[\theta \left(\theta^{2}+\theta +2\right)\right]}^{-1}$ for $\theta \in R_{+}$ (shape parameter). This parameterization is particularly useful since our primary goal is to derive a regression model, in which the influence of fixed-effects can be evaluated directly over the mean of a zero-modified response variable. Unlike in zero-inflated models, here parameter *p* is defined on the interval $\left[0,P{\left(Y>0;\mu \right)}^{-1}\right]$, and so the ZMPS model is not a mixture distribution since *p* may assume values greater than 1. The expected value and variance of *Y* are given, respectively, by E(Y)=λ=μp and $V\left(Y\right)=\varsigma^{2}=p\left[\sigma^{2}+\left(1-p\right)\mu^{2}\right]$, where $\sigma^{2}\in R_{+}$ is the variance of the PS distribution (see [43], Table 4).

The hurdle version of the PS distribution can be obtained by taking ω=pP(Y>0;μ), and so rewriting Equation (1) as

(3) $$P_{*}\;\left(Y=y;\mu ,\omega \right)=\left(1-\omega \right)\delta_{y}+\omega \;P^{*}\;\left(Y=y;\mu \right),\;\;\;\;y\in N_{0},$$

for ω∈[0,1] and where $P^{*}\left(Y=y;\mu \right)$ is the pmf of the zero-truncated Poisson–Sujatha (ZTPS) distribution [44]. Noticeably, Equation (3) is only a reparameterization of the standard ZMPS, and so one can conclude that these models are interchangeable. For ease of notation and understanding, the acronym ZMPS will be used when we refer to the hurdle version of the PS distribution.

The corresponding cumulative distribution function (cdf) of *Y* is given by

(4) $$\begin{array}{ccc} F^{*}\;\left(y;\mu ,\omega \right) & = & 1-\frac{\omega }{P(Y>0;\mu )}\left\{\frac{yh\left(\mu \right)\left[h^{2}\left(\mu \right)+\left(y+6\right)h\left(\mu \right)+2\right]}{\left[h^{2}\left(\mu \right)+h\left(\mu \right)+2\right]{\left[h\left(\mu \right)+1\right]}^{y+3}}+\right.\\ & & \left.\frac{h^{4}\left(\mu \right)+4h^{3}\left(\mu \right)+10h^{2}\left(\mu \right)+7h\left(\mu \right)+2}{\left[h^{2}\left(\mu \right)+h\left(\mu \right)+2\right]{\left[h\left(\mu \right)+1\right]}^{y+3}}\right\},\;\;\;\;y\in N_{0}. \end{array}$$

Comparatively, the proposed model can be considered more flexible than zero-inflated models as it allows for zero-deflation, which is a structure often encountered when handling count data (see, for example, [45,46]). Besides, it can incorporate overdispersion that does not come only from inflation/deflation of zeros, as one of its parts is dedicated to describing the positive values’ behavior. In the regression framework that we have developed, discrepant points (outliers) can be identified, and through a careful sensitivity analysis, it is possible to quantify the influences of such observations. However, since the PS distribution accounts for different levels of overdispersion, its zero-modified version is naturally a robust alternative, as it may accommodate discrepant points that would significantly impact the parameter estimates of the ZMP model.

In this paper, the inferential procedures are conducted under a fully Bayesian perspective—an adaptation of the g-prior method [47] for the fixed-effects parameters is considered. The random-walk metropolis algorithm was used to draw pseudo-random samples from the posterior distribution of the model parameters. Local influence measures based on some well-known divergences were considered for the task of detecting influential points. Model validation metrics such as the Bayesian *p*-value and the randomized quantile residuals are presented. Intensive Monte Carlo simulation studies were performed to assess Bayesian estimators’ empirical properties; the obtained results are discussed, and the overall performance of the adopted methodology was evaluated. Additionally, an application using a real dataset is presented to assess the proposed model’s usefulness and competitivity.

This paper is organized as follows. In Section 2, we present the fixed-effects regression model based on the hurdle version of the PS distribution. In Section 3, we describe all the Bayesian methodologies and associated numerical procedures considered for inferential purposes. In Section 4, we discuss the results of an intensive simulation study, and in Section 5, a real data application using the proposed model is exhibited. General comments and concluding remarks are addressed in Section 6.

## 2. The ZMPS Regression Model

Suppose that a random experiment (designed or observational) is conducted with *n* subjects. The primary response for such an experiment is described by a discrete random variable $Y_{i}$ denoting the outcome for the *i*-th subject. The full response vector is given by $Y=(Y_{1},\ldots ,Y_{n})$, and we assume that the observed vector y is obtained conditionally to fixed-effects, here denoted by $\beta =(\beta_{1},\beta_{2})$. Assuming that $Y_{i}|\beta ∼\mathrm{ZMPS}\left(\mu_{i},\omega_{i}\right)$ holds for all *i*, a general fixed-effects regression model for count data based on the ZMPS distribution can be derived by rewriting Equation (3) as

(5) $$P_{*}\;\left(Y_{i}=y_{i};\beta \right)=\left(1-\omega_{i}\right)\delta_{y_{i}}+\omega_{i}P^{*}\;\left(Y_{i}=y_{i};\mu_{i}\right),\;\;\;\;y_{i}\in N_{0},$$

where $\mu_{i}\equiv \mu \left(x_{1i},\beta_{1}\right)$ and $\omega_{i}\equiv \omega \left(x_{2i},\beta_{2}\right)$ are parameterized nonlinear functions. In this framework, we have $\beta_{k}^{⊺}=\left(\beta_{k0},\ldots ,\beta_{kq_{k}}\right)$(k=1,2) related to $x_{ki}^{⊺}=\left(1,x_{ki}^{1},\ldots ,x_{ki}^{q_{k}}\right)$, where $x_{ki}$ is a vector of covariates that may include, for example, dummy variables, cross-level interactions, and polynomials. The quantity $q_{1}$$\left(q_{2}\right)$ denotes the number of covariates considered in the systematic component of a linear predictor for parameter $\mu_{i}$$\left(\omega_{i}\right)$. The full regression matrices of model (5) can be written as $X_{k}=\left(1_{n},X_{k,n\times q_{k}}\right)$, where $1_{n}$ is the intercept column and the submatrix $X_{k,n\times q_{k}}$ is defined in such a way that its *i*-th row contains the vector $\left(x_{ki}^{1},\ldots ,x_{ki}^{q_{k}}\right)$. The overall dimension of $X_{k}$ is $n\times (q_{k}+1)$.

Now, we have to specify two monotonic, invertible, and twice differentiable link functions, say $g_{1}$ and $g_{2}$, in which $\mu_{i}=g_{1}^{-1}\left(x_{1i}^{⊺}\beta_{1}\right)$ and $\omega_{i}=g_{2}^{-1}\left(x_{2i}^{⊺}\beta_{2}\right)$ are well defined on $R_{+}$ and (0,1), respectively. For this purpose, one may choose any suitable mappings $g_{1}$ and $g_{2}$ such that $g_{1}^{-1}\;:R\to R_{+}$ and $g_{2}^{-1}\;:R\to \left(0,1\right)$. The logarithm link function, $\log\left(\mu_{i}\right)=x_{1i}^{⊺}\beta_{1}$, is the natural choice for $g_{1}$. For $g_{2}$, the popular choice is the logit link function,

(6) $$\mathrm{logit}\left(\omega_{i}\right)=\log\left(\frac{\omega_{i}}{1-\omega_{i}}\right)=x_{2i}^{⊺}\beta_{2}.$$

The probit link function,

(7) $$\Phi^{-1}\left(\omega_{i}\right)=x_{2i}^{⊺}\beta_{2},$$

is also appropriate for the requested purpose. Another possible choice for $g_{2}$ is

(8) $$\log\left[-\log\left(1-\omega_{i}\right)\right]=x_{2i}^{⊺}\beta_{2},$$

which corresponds to the complementary log–log link function. One can notice that these link functions exclude the limit cases $p_{i}=0$ and $p_{i}=P{\left(Y>0;\mu_{i}\right)}^{-1}$. The link Function (8) is usually preferable when the occurrence probability of a specific outcome is considerably high/low as it accommodates asymmetric behaviors on the unit interval, which is not the case for link Functions (6) and (7). Besides, a more sophisticated approach considering power and reversal power link functions was proposed by [48], and can also be used to add even more flexibility when modeling parameter $\omega_{i}$.

We may refer to the proposed model as a “semi-compatible” regression model. The term “compatible” alludes to “zero-altered,” which defines the class proposed by [49], and extended by [50] in a setting including semiparametric zero-altered models that accommodate over/underdispersion. Zero-altered models are similar to zero-modified ones, but the compatibility arises from the linear predictors of $\mu_{i}$ and $\omega_{i}$ being the same. In our case, specifically, it is worthwhile to mention that identifiability problems may occur if one considers a fixed-effects regression model derived directly from (3), with parameters μ and *p* sharing covariates, even if $\beta_{2}\neq \beta_{1}$. Therefore, the adopted structure allows for more flexibility and robustness as μ and ω may share covariates not necessarily with $\beta_{2}=\beta_{1}$, and so the only requirement for ensuring model identifiability is the linear independence between covariates within linear predictors.

Given a set of covariates, the probability of a zero-valued count being observed for the *i*-th subject is given by $1-g_{2}^{-1}\left(x_{2i}^{⊺}\beta_{2}\right)$. Under the logistic regression model (6), $\beta_{2l}$$\left(l=1,\ldots ,q_{2}\right)$ represents the direct change in the log-odds of $Y_{i}$, it being positive per 1-unit change in $x_{2i}^{l}$, while holding the other covariates at fixed values. On the other hand, the same not apply if one adopts the link Function (8) since $e^{\beta_{2l}}$ is not the odds ratio for the *l*-th covariate effect, and so $\beta_{2l}$ does not have a straightforward interpretation in terms of contribution to log-odds. Likewise, it is not possible to interpret the coefficients of the probit model (7) directly, but one can evaluate the marginal effect of $\beta_{2l}$ by analyzing how much the conditional probability of $Y_{i}$ being positive is affected when the value of $x_{2i}^{l}$ is changed. The exact interpretation of $\beta_{1l}$$\left(l=1,\ldots ,q_{1}\right)$ is not direct in terms of the mean of the hurdle model since the positive counts are modeled by a zero-truncated distribution (ZTPS), and therefore, $\beta_{1l}$ represents the overall effect of $x_{1i}^{l}$ on the expected value $\mu_{i}$ when $y_{i}>0$, while holding the other covariates at fixed values.

The proposed model has $d=\dim\left(\beta \right)=q_{1}+q_{2}+2$ unknown quantities to be estimated. A fully Bayesian approach will be considered for parameter estimation and associated inference. The next section is dedicated to present details of such an approach.

## 3. Inference

In this section, we address the problem of estimating and making inferences about the proposed model from a fully Bayesian perspective. Firstly, we derive the model likelihood function, and then, a suitable set of prior distributions is considered to obtain a computationally tractable posterior density for the vector β. Beyond the primary distributional assumption that $Y_{i}|\beta ∼\mathrm{ZMPS}\left(\mu_{i},\omega_{i}\right)$ holds for all *i*, here we also assume that the outcomes for different subjects are unconditionally independent.

Let *Y* be a discrete random variable assuming values on $N_{0}$. Suppose that a random experiment is carried out *n* times independently and, subject to $x_{ki}$ for each *i*, a vector $y=(y_{1},\cdots ,y_{n})$ of observed values from *Y* is obtained. Considering model Formulation (5), the likelihood function of β can be written as

$$\begin{array}{ccc} L\left(\beta ;y\right) & = & \prod_{i=1}^{n}\omega_{i}{\left(\frac{1-\omega_{i}}{\omega_{i}}\right)}^{\delta_{y_{i}}}{\left[\frac{P\left(Y_{i}=y_{i};\mu_{i}\right)}{P\left(Y_{i}>0;\mu_{i}\right)}\right]}^{1-\delta_{y_{i}}}\\ & = & \prod_{i=1}^{n}g_{2}^{-1}\left(x_{2i}^{⊺}\beta_{2}\right){\left[\frac{1-g_{2}^{-1}\left(x_{2i}^{⊺}\beta_{2}\right)}{g_{2}^{-1}\left(x_{2i}^{⊺}\beta_{2}\right)}\right]}^{\delta_{y_{i}}}{\left\{\frac{P\left[Y_{i}=y_{i};g_{1}^{-1}\left(x_{1i}^{⊺}\beta_{1}\right)\right]}{P\left[Y_{i}>0;g_{1}^{-1}\left(x_{1i}^{⊺}\beta_{1}\right)\right]}\right\}}^{1-\delta_{y_{i}}}, \end{array}$$

and so the corresponding log-likelihood function is given by

(9) $$\begin{array}{ccc} \ell \left(\beta ;y\right) & = & \sum_{i=1}^{n}\left(1-\delta_{y_{i}}\right)\log\left\{\frac{P\left[Y_{i}=y_{i};g_{1}^{-1}\left(x_{1i}^{⊺}\beta_{1}\right)\right]}{P\left[Y_{i}>0;g_{1}^{-1}\left(x_{1i}^{⊺}\beta_{1}\right)\right]}\right\}+\\ & & \sum_{i=1}^{n}\left\{\log\left[g_{2}^{-1}\left(x_{2i}^{⊺}\beta_{2}\right)\right]-\delta_{y_{i}}\log\left[\frac{g_{2}^{-1}\left(x_{2i}^{⊺}\beta_{2}\right)}{1-g_{2}^{-1}\left(x_{2i}^{⊺}\beta_{2}\right)}\right]\right\}\\ & = & \ell_{1}\left(\beta_{1};y\right)+\ell_{2}\left(\beta_{2};y\right). \end{array}$$

In this work, we will consider a log-linear model for parameter $\mu_{i}$, that is, $g_{1}\left(\mu_{i}\right)=\log\left(\mu_{i}\right)=x_{1i}^{⊺}\beta_{1}$. The choice of $g_{2}$ is left open and the notation $\omega_{i}=g_{2}^{-1}\left(x_{2i}^{⊺}\beta_{2}\right)$ will be used when necessary. From Equation (9), one can easily notice that the vectors $\beta_{1}$ and $\beta_{2}$ are orthogonal and that $\ell_{1}$ depends only on the positive values of y. In this way, the log-likelihood function of $\beta_{1}$ takes the form

(10) $$\begin{array}{ccc} \ell_{1}\left(\beta_{1};y\right) & = & \sum_{j\in J_{1}}\log\left\{y_{j}^{2}+y_{j}\left[h\left(e^{x_{1j}^{⊺}\beta_{1}}\right)+4\right]+\left[h^{2}\left(e^{x_{1j}^{⊺}\beta_{1}}\right)+3h\left(e^{x_{1j}^{⊺}\beta_{1}}\right)+4\right]\right\}-\\ & & \sum_{j\in J_{1}}\log\left[h^{4}\left(e^{x_{1j}^{⊺}\beta_{1}}\right)+4h^{3}\left(e^{x_{1j}^{⊺}\beta_{1}}\right)+10h^{2}\left(e^{x_{1j}^{⊺}\beta_{1}}\right)+7h\left(e^{x_{1j}^{⊺}\beta_{1}}\right)+2\right]+\\ & & 3\sum_{j\in J_{1}}\log\left[h\left(e^{x_{1j}^{⊺}\beta_{1}}\right)\right]-\sum_{j\in J_{1}}y_{j}\log\left[h\left(e^{x_{1j}^{⊺}\beta_{1}}\right)+1\right], \end{array}$$

where $J_{1}=\left\{j:y_{j}>0,y_{j}\in y\right\}$ is the finite set of indexes regarding the positive observations of y. Adopting this setup is equivalent to assuming that each positive element of y comes from a ZTPS distribution. Here, we are extending the fact that estimating the P parameter θ using the zero-truncated Poisson (ZTP) distribution results in a loss of efficiency in the inference if there is no zero modification [35,37]. Now, the log-likelihood function of $\beta_{2}$ can be written as

(11) $$\ell_{2}\left(\beta_{2};y\right)=\sum_{i=1}^{n}\log\left[g_{2}^{-1}\left(x_{2i}^{⊺}\beta_{2}\right)\right]-\sum_{j\in J_{2}}\log\left[\frac{g_{2}^{-1}\left(x_{2j}^{⊺}\beta_{2}\right)}{1-g_{2}^{-1}\left(x_{2j}^{⊺}\beta_{2}\right)}\right],$$

where $J_{2}=\left\{j:y_{j}=0,y_{j}\in y\right\}$ is the finite set of indexes regarding the zero-valued observations of y.

### 3.1. Prior Distributions

The g-prior [47] is a popular choice among Bayesian users of the multiple linear regression model, mainly due to the fact of providing a closed-form posterior distribution for the regression coefficients. The g-prior is classified as an objective prior method which uses the inverse of the Fisher information matrix up to a scalar variance factor to obtain the prior correlation structure of the multivariate normal distribution. Such specification is quite attractive since the Fisher information plays a major role in determining large-sample covariance in both Bayesian and classical inference.

The problem of eliciting conjugate priors for a GLM was addressed by [51]. Their approach can be considered as a generalization of the original g-prior method. Still, its application is restricted for the class of GLMs since the proposed prior does not have closed-form for non-normal exponential families. Alternatively, ref. [52] have proposed the information matrix prior as a way to assess the prior correlation structure between the coefficients, not including the intercept since the regression matrix is centered as to ensure that $\beta_{0}$ is orthogonal to the other coefficients. This method uses the Fisher information similarly to a precision matrix whose elements are shrunken by a fixed variance factor. However, the authors have pointed out that such class of priors can only be considered Gaussian priors if the Fisher information matrix does not depend on the vector $\beta^{\prime }=\left(\beta_{1},\cdots ,\beta_{q}\right)$. In this way, ref. [53] had considered a similar approach when they proposed a class of hyper-g priors for GLMs, where the precision matrix is evaluated at the prior mode, hence obtaining an information matrix that is $\beta^{\prime }$ free.

The formal concept behind the information matrix prior is closely related to the unit information prior [54], whose main idea is that the amount of information provided by a prior distribution must be the same as the amount of information contained in a single observation. Such an idea can be applied in the previously mentioned approaches by simply considering the total sample size (n) as the variance factor. Ref. [52] have also considered fixed values for the scalar variance factor. On the other hand, some works, including [53,55,56] do consider prior elicitation and inference procedures for the variance scale factor. Here, we will adopt a methodology based on the unit information prior idea combined with the “noninformative g-prior” proposed by [57] for binary regression models. Based on such an approach, it is possible to obtain a quite simple prior distribution for the fixed-effects of the proposed model as $\beta_{k}∼N_{{\bar{q}}_{k}}\left(0,n{\left(X_{k}^{{}^{⊺}}X_{k}\right)}^{-1}\right)$, where ${\bar{q}}_{k}=q_{k}+1$.

It is worthwhile to mention that, in cases where $X_{k}$ is rank deficient $\left(n<q_{k}+1\right)$ or contains collinear covariates, it is highly advisable to compute the generalized inverse of $X_{k}^{{}^{⊺}}X_{k}$ otherwise the prior covariance matrix of $\beta_{k}$ may not be defined.

Analogously to Marin and Robert’s approach, we do not consider centered regression matrices in the prior specification. Hence, we are able to include $\beta_{10}$ in the proposed g-prior but, in this case, the intercept is a priori correlated with the other coefficients $\left(\beta_{11},\cdots ,\beta_{1q_{1}}\right)$. The same applies for $\beta_{20}$ and the vector $\left(\beta_{21},\cdots ,\beta_{2q_{2}}\right)$.

### 3.2. Posterior Distributions and Estimation

Considering the outlined structure for the ZMPS regression model, the unnormalized joint posterior distribution of the unknown vector β is given by

(12) $$\pi \left(\beta ;y\right)\propto \exp\left\{\ell \left(\beta ;y\right)\right\}\pi \left(\beta \right).$$

However, since $\beta_{1}$ and $\beta_{2}$ are orthogonal, we have that

(13) $$\pi_{1}\left(\beta_{1};y\right)\propto \exp\left\{\ell_{1}\left(\beta_{1};y\right)\right\}\pi_{1}\left(\beta_{1}\right)\;\;\;\;\mathrm{and}\;\;\;\;\pi_{2}\left(\beta_{2};y\right)\propto \exp\left\{\ell_{2}\left(\beta_{2};y\right)\right\}\pi_{2}\left(\beta_{2}\right),$$

where $\ell_{1}$ and $\ell_{2}$ are given by (10) and (11), respectively. Naturally, in the discrete setting, the use of proper (Gaussian) priors prevents $\pi_{1}$ and $\pi_{2}$ from being improper.

From the Bayesian point of view, inferences for the elements of $\beta_{k}$ can be obtained from their marginal posterior distributions. However, deriving analytical expressions for these densities is infeasible, mainly due to the associated log-likelihood function’s complexity. In this case, to make inferences for $\beta_{k}$, we must resort to a suitable iterative procedure to drawn pseudo-random samples from their posterior densities. Hence, aiming to generate *N* chains for $\beta_{k}$, we will adopt the well-known random-walk metropolis (RwM) algorithm [58,59]. For the posterior densities in (13), we consider a multivariate normal distributions for the proposal (candidate-generating) densities in the algorithm. These distributions will be used as the main terms in the transition kernels when computing the acceptance probabilities. Hence, at any state t>0, the MCMC simulation are performed by proposing a candidate $\psi_{k}$ for $\beta_{k}$ as

$$\psi_{k}|\beta_{k}^{\left(t-1\right)}∼N_{{\bar{q}}_{k}}\;\left[\nu \beta_{k}^{\left(t-1\right)},\nu S_{k}^{\left(t-1\right)}\right],$$

where $\nu =n{\left(n+1\right)}^{-1}$. One can notice that transitions depend on the acceptance of pseudo-random vectors generated with mean given by the actual state of the chain, which is shrunken by the factor ν. Besides, at any state t>0, the covariance matrix of the candidate vector $\psi_{k}$ can be approximated numerically by evaluating $S_{k}=H_{k}^{-1}$ at $\beta_{k}=\nu \beta_{k}^{\left(t-1\right)}$, where

$$H_{k}=-\frac{\partial^{2}\log\left[\pi_{k}\left(\beta_{k};y\right)\right]}{\partial \beta_{k}\partial \beta_{k}^{⊺}}.$$

The procedure to generate pseudo-random samples from the approximate posterior distribution of β is summarized in Algorithm A1 (see Appendix A). To run it, one has to specify the size of chains to be generated (N) and the initial state vectors $\beta_{1}^{\left(0\right)}$ and $\beta_{2}^{\left(0\right)}$ beforehand. For a specific asymptotic Gaussian environment, [59] have shown that the optimal acceptance rate should be around 45% for 1-dimensional problems and asymptotically approaches to 23.40% in higher-dimensional problems. We consider acceptance rates varying between 23.40% and 32% as quite reasonable since the proposed model will generally have at least four parameters to be estimated. Indeed, the higher the value of *n*, the lower the acceptance rate in the RwM algorithm, which results in lower variability of estimates.

The convergence of the simulated sequences can be monitored by using trace and autocorrelation plots, and the run-length control method with a half-width test [60], the Geweke *z*-score diagnostic [61], and the Brooks-Gelman-Rubin scale-reduction statistic [62]. After diagnosing convergence, some samples can be discarded as burn-in. The strategy to decrease the correlation between and within generated chains is based on getting thinned steps, and so the final sample is supposed to have size M≪N for each parameter. A full descriptive summary of the posterior distribution (12) can be obtained through Monte Carlo (MC) estimators using the sequence ${\left\{\beta^{t}\right\}}_{t=1}^{M}$. We choose the posterior expected value as the Bayesian point estimator for θ, that is,

(14) $$\hat{\beta }=\frac{1}{M}\sum_{t=1}^{M}\beta^{\left(t\right)},$$

which is also known as the minimum mean square error estimator.

In the next section, we discuss the results of the Monte Carlo simulation studies performed to assess the proposed Bayesian methodology’s performance. In Section 5, the proposed model’s usefulness and competitivity are illustrated by using a real dataset. All computations were performed using the R environment [63]. The executable scripts were made available at the publisher’s website.

### 3.3. Posterior Predictive Distribution

In a Bayesian context, the posterior predictive distribution (ppd) is defined as the distribution of possible future (unobserved) values conditioned on the observed ones. Under the ZMPS distribution, the pmf of any observation $w\in N_{0}$ (subject to the vectors $x_{1w}^{⊺}$ and $x_{2w}^{⊺}$ of covariates) is given by

$$\begin{array}{ccc} P_{\pi }\;\left(Y=w\right) & = & \int_{R^{d}}P^{*}\;\left(Y=w;\mu_{w},\omega_{w}\right)\pi \left(\beta ;y\right)\;d\beta \\ & = & \int_{R^{{\bar{q}}_{1}}}{\left[\frac{P\;\left(Y=w;e^{x_{1w}^{⊺}\beta_{1}}\right)}{P\;\left(Y>0;e^{x_{1w}^{⊺}\beta_{1}}\right)}\right]}^{1-\delta_{w}}\pi_{1}\left(\beta_{1};y\right)\;d\beta_{1}\times \\ & & \int_{R^{{\bar{q}}_{2}}}g_{2}^{-1}\left(x_{2w}^{⊺}\beta_{2}\right){\left[\frac{1-g_{2}^{-1}\left(x_{2w}^{⊺}\beta_{2}\right)}{g_{2}^{-1}\left(x_{2w}^{⊺}\beta_{2}\right)}\right]}^{\delta_{w}}\pi_{2}\left(\beta_{2};y\right)\;d\beta_{2}, \end{array}$$

where $\delta_{w}=1$ if w=0 and $\delta_{w}=0$ otherwise. Noticeably, the ppd has no closed-form available, and therefore, an MC estimator for this quantity is given by

(15) $${\hat{P}}_{\pi }\;\left(Y=w\right)=\frac{1}{M^{2}}\sum_{t=1}^{M}g_{2}^{-1}\left(x_{2w}^{⊺}\beta_{2}^{\left(t\right)}\right){\left[\frac{1-g_{2}^{-1}\left(x_{2w}^{⊺}\beta_{2}^{\left(t\right)}\right)}{g_{2}^{-1}\left(x_{2w}^{⊺}\beta_{2}^{\left(t\right)}\right)}\right]}^{\delta_{w}}\sum_{t=1}^{M}b_{t}\left(w\right),$$

where

$$\begin{array}{c} b_{t}\left(w\right)=\left\{\frac{h^{3}\;\left(e^{x_{1w}^{⊺}\beta_{1}^{\left(t\right)}}\right)}{h^{4}\;\left(e^{x_{1w}^{⊺}\beta_{1}^{\left(t\right)}}\right)+4h^{3}\;\left(e^{x_{1w}^{⊺}\beta_{1}^{\left(t\right)}}\right)+10h^{2}\;\left(e^{x_{1w}^{⊺}\beta_{1}^{\left(t\right)}}\right)+7h\;\left(e^{x_{1w}^{⊺}\beta_{1}^{\left(t\right)}}\right)+2}\right.\times \\ {\left.\frac{w^{2}+w\left[h\;\left(e^{x_{1w}^{⊺}\beta_{1}^{\left(t\right)}}\right)+4\right]+\left[h^{2}\;\left(e^{x_{1w}^{⊺}\beta_{1}^{\left(t\right)}}\right)+3h\;\left(e^{x_{1w}^{⊺}\beta_{1}^{\left(t\right)}}\right)+4\right]}{{\left[h\;\left(e^{x_{1w}^{⊺}\beta_{1}^{\left(t\right)}}\right)+1\right]}^{w}}\right\}}^{1-\delta_{w}}. \end{array}$$

From Equation (15), one can easily estimate, for example, the posterior probability of Y=0 (subject to $x_{10}^{⊺}$ and $x_{20}^{⊺}$) as

$${\hat{P}}_{\pi }\;\left(Y=0;x_{10}^{⊺},x_{20}^{⊺}\right)=\frac{1}{M}\sum_{t=1}^{M}g_{2}^{-1}\left(x_{20}^{⊺}\beta_{2}^{\left(t\right)}\right)\left[\frac{1-g_{2}^{-1}\left(x_{20}^{⊺}\beta_{2}^{\left(t\right)}\right)}{g_{2}^{-1}\left(x_{20}^{⊺}\beta_{2}^{\left(t\right)}\right)}\right].$$

## 4. Simulation Study

The empirical properties of an estimator can be accessed through Monte Carlo simulations. In this way, we have performed an intensive simulation study aiming to validade the Bayesian approach in some specific situations. The simulation process was carried out by generating 500 pseudo-random samples of sizes n=50,100,200,and500 of a variable *Y* following a ZMPS distribution under the regression framework presented in Section 2. For the whole process, it was considered a n×2 regression matrix $X_{1}=\left(1_{n},X_{1,n\times 1}\right)$ in which $X_{n\times 1}$ is a vector containing *n* generated values from a Uniform distribution on the unit interval. Here, we have fixed $X_{2}=X_{1}$. Moreover, we have assigned different values for the vectors $\beta_{1}^{⊺}=\left(\beta_{10},\beta_{11}\right)$ and $\beta_{2}^{⊺}=\left(\beta_{20},\beta_{21}\right)$ in order to generate both zero-inflated and zero-deflated artificial samples. The logarithm link function was considered for $g_{1}$. For $g_{2}$, we have considered the link Functions (6)–(8) as a way to evaluate how these different specifications affect the estimation of β.

Algorithm A2 (see Appendix A) can be used to generate a single pseudo-random realization from the ZMPS distribution in the regression framework with covariate U(0,1) for μ and ω. The extension for the use of more covariates is straightforward. The process to generate a pseudo-random sample of size *n* consists of running the algorithm as often as necessary, say $n^{*}$ times $\left(n^{*}⩾n\right)$. The sequential search is a black-box algorithm and works with any computable probability vector. The main advantage of such a procedure is its simplicity. On the other hand, sequential search algorithms may be slow as the while-loop may have to be repeated very often. More details about this algorithm can be found at [64].

Under the ZMPS distribution, the expected number of iterations (NI), that is, the expected number of comparisons in the while condition, is given by

$$E\;\left(\mathrm{NI}\right)=\lambda +1=\frac{\omega \mu \left[h^{2}\left(\mu \right)+h\left(\mu \right)+2\right]{\left[h\left(\mu \right)+1\right]}^{3}}{h^{4}\left(\mu \right)+4h^{3}\left(\mu \right)+10h^{2}\left(\mu \right)+7h\left(\mu \right)+2}+1,$$

where h(μ) is given by Equation (2).

We have considered four scenarios for each kind of zero-modification. Table 1 presents the true parameter values that were considered in our study. For the zero-inflated (zero-deflated) case, the samples were generated from the ZMPS distribution by considering that $p_{i}\in \left(0,1\right)$$\left(p_{i}\in \left[1,P{\left(Y>0;\mu_{i}\right)}^{-1}\right]\right)$ for all *i*. Here, the regression coefficients were chosen by taking into account that zero-inflated (zero-deflated) samples have, naturally, proportion of zeros greater (lower) than expected under an ordinary count distribution and therefore, the variable $Y_{i}$(i=1,…,n) was generated with mean far from zero (close to zero). Table 1 also presents the range of parameters $\mu_{i}$ and $p_{i}$ in each scenario. The bounds were obtained by evaluating the linear predictors $\beta_{10}+\beta_{11}x$ and $\beta_{20}+\beta_{21}x$ at x=0 and x=1 (limit values of the adopted covariate). Scenarios 1 and 2 of the zero-inflated case were considered to illustrate the Bayesian estimators’ behavior when the proposed model is used to fit (right) long-tailed count data.

To apply the proposed Bayesian approach to each scenario, we have considered the RwM algorithm for MCMC sampling. For each generated sample, a chain with N= 50,000 values was generated for each parameter, considering a burn-in period of 20% of the chain size. To obtain pseudo-independent samples from the posterior distributions given in (13), one out every 10 generated values were kept, resulting in chains of size M=4000 for each parameter. Using trace plots and Geweke’s *z*-score diagnostic, the remaining chains’ stationarity was revealed. When running the simulations, the acceptance rates were ranging between 23.40% and 32%. The posterior mean (14) was considered as the Bayesian point estimator, and its performance was studied by assessing its bias (B), its mean squared error (MSE), and its mean absolute percentage error (MAPE). Besides, the coverage probability (CP) of the 95% highest posterior density intervals (HPDIs) was also estimated.

Using the generated samples and letting $\gamma =\beta_{10},\beta_{11},\beta_{20}\;\;\mathrm{or}\;\;\beta_{21}$, the MC estimators for these measures are given by

$${\hat{B}}_{\hat{\gamma }}=\frac{1}{500}\sum_{j=1}^{500}\left({\hat{\gamma }}_{j}-\gamma \right),\;{\hat{\mathrm{MSE}}}_{\hat{\gamma }}=\frac{1}{500}\sum_{j=1}^{500}{\left({\hat{\gamma }}_{j}-\gamma \right)}^{2},\;\mathrm{and}\;{\hat{\mathrm{MAPE}}}_{\hat{\gamma }}=\frac{1}{500}\sum_{j=1}^{500}\left|\frac{{\hat{\gamma }}_{j}-\gamma }{\gamma }\right|.$$

The variance of $\hat{\gamma }$ was estimated as the difference between the MSE and the square of the bias. Moreover, the CP of the HPDIs was estimated by

$${\hat{\mathrm{CP}}}_{\gamma }=\frac{1}{500}\sum_{j=1}^{500}\delta_{j}\;\left(\gamma \right),$$

where $\delta_{j}\left(\gamma \right)$ assumes 1 if the *j*-th HPDI contains the true value γ and 0 otherwise. We have also estimated the below noncoverage probability (BNCP) and the above noncoverage probability (ANCP) of the HPDIs. These measures are computed analogously to CP. The BNCP and ANCP may be useful measures to determine asymmetrical behaviors as they provide the probabilities of finding the actual value of γ on the tails of its posterior distribution.

Due to the massive amount of results, the obtained results were made available on the publisher’s website as supplementary material. In our study, we have noticed that, as expected, the parameter estimates became more accurate with increasing sample sizes since the estimated biases and mean squared errors have decreased considerably as *n* increased. The squared ratio between the mean squared error and the estimated variance approaches 1 as *n* increases. Although high MAPE values were obtained for some parameters (when using small sample sizes), this does not compromise the overall estimation accuracy. For example, when n=100, we have obtained a estimated MAPE value of approximately 56% for $\beta_{11}$ (see Table S25, Scenario S1, Supplementary Material). Taking into account the true value of such parameter (1.00), we have that the estimates for $\beta_{11}$ were ranging mostly between 0.44 and 1.56, which do not represent a significant impact on the estimated mean (μ). When (right) long-tailed count data are available, the CP of the HPDI for $\beta_{11}$ is considerably lower than the adopted nominal level (for small sample sizes) as its posterior distribution tends to be more asymmetric towards higher values on the parameter space. However, we have observed that the estimated CP of the HPDIs is converging to 95% in both zero-modified cases, and the posterior distributions became more symmetric with increasing sample sizes.

Considering the predefined scenarios, we conclude that our simulation study provides favorable indications about the adopted Bayesian methodology’s suitability to estimate the parameters of the proposed model. We believe that in a similar procedure with a different set of actual values, the estimators’ overall behavior should resemble the results that we have described here. Besides, the adopted methodology would also be reliable if one or more than one covariates (possibly of other nature) were included in the linear predictors of $\mu_{i}$ and $\omega_{i}$.

## 5. Chromosomal Aberration Data Analysis

In this section, the ZMPS regression model is considered for analyzing a real dataset obtained from a cytogenetic dosimetry experiment that was first presented by [65]. In this study, the response variable is the number of cytogenetic chromosomal aberrations after the DNA molecule is treated with induced radiation. The dataset was obtained by irradiating five blood samples from a healthy donor with different doses $x_{i}$(i=1,⋯,5) ranging between 0.1 and 1.0 Gy with 2.1 MeV neutrons in three different culture times (48 h, 56 h, and 72 h), considering partial-body exposure-densely ionizing radiation. In the following, $n_{i}$ cells were examined in each irradiated sample and the number of dicentrics and centric ring aberrations $y_{ij}$$\left(j=1,\cdots ,n_{i}\right)$ was recorded.

While [65] have used a *t*-test to analyze whether the averages of the relative number of dicentrics plus centric ring aberration frequencies differed significantly between the three different culture times, we are primarily interested in evaluating if the averages of the number of dicentrics plus centric ring aberration differ significantly between doses of ionizing radiation, considering data from culture times of 72 h.

**Figure 1:**
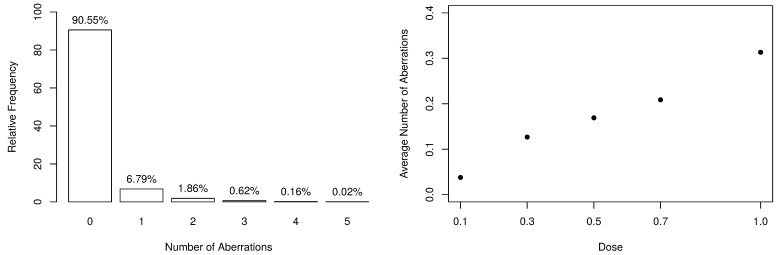
Summary of the numbers of dicentrics and centric ring aberrations.

The frequency distribution of the collected data is available in Table 2, along with some descriptive statistics. From the observed dataset, there exist evidences that the response variable is slightly overdispersed since ${\bar{y}}_{.}=0.131<s_{.}^{2}=0.210$ and $s_{.}^{2}/{\bar{y}}_{.}=1.607$. Additionally, the number of aberrations appears to be heavily zero-inflated, as shown in the left-panel of Figure 1. On the other hand, one can notice that, as the dose of ionizing radiation increases, the number of observed zeros decreases. Still, the distribution becomes more overdispersed since it naturally increases the number of aberrations.

According to [32], when considering higher linear energy transfer radiations, the incidence of chromosomal aberrations becomes a linear function of the dose because the more densely ionizing nature of the radiation leads to an “one track” distribution of damage. Such an aspect can be seen in the right-panel of Figure 1, which highlights the linear behavior between the average number of aberrations and the doses. In this way, our assumption is that $Y_{ij}|x_{i}∼\mathrm{ZMPS}\left(\mu_{ij},\omega_{ij}\right)$, where parameters $\mu_{ij}$ and $\omega_{ij}$ are specified as linear dose models, that is,

$$\log\left(\mu_{ij}\right)=\beta_{10}+\beta_{11}x_{i}\;\;\;\;\mathrm{and}\;\;\;\;g_{2}\left(\omega_{ij}\right)=\beta_{20}+\beta_{21}x_{i}.$$

To fit the ZMPS regression model with dose as the only covariate, we have adopted the same procedure used in the previous section. The link Function (7) was chosen to relate $\omega_{ij}$ with the linear predictor $\beta_{20}+\beta_{21}x_{i}$ and so we have the probit hurdle regression model. In this framework, the coefficient $\beta_{11}$ represents the effect of the dose of ionizing radiation on the expected count $\mu_{i}$ when $Y_{ij}>0$, and $\beta_{21}$ indicates the effect of the dose on the probability of aberrations to occur. We have considered the RwM for MCMC sampling, generating a chain of size N= 50,000 for each parameter whereby the first 10,000 values were discarded as burn-in. The stationarity of the chains was revealed using the Geweke *z*-score diagnostic of convergence. To obtain the pseudo-independent samples from the posterior distributions given in (13), we have considered one value out of every 10 generated ones, resulting in chains of size M=4000 for each parameter.

**Figure 2:**
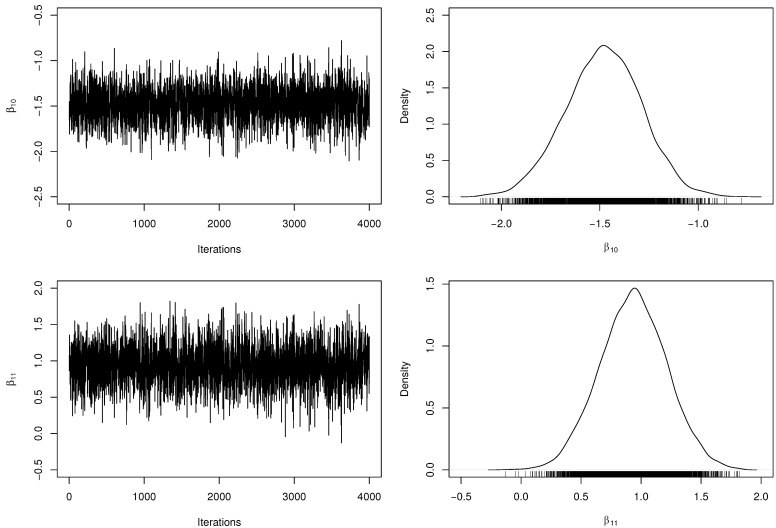
Trace plots and marginal posterior distributions of parameters $\beta_{10}$ and $\beta_{11}$ from the ZMPS regression model.

**Figure 3:**
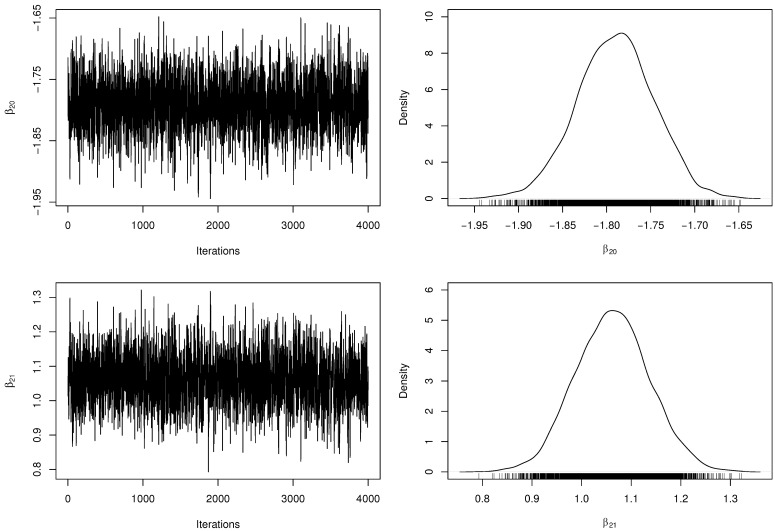
Trace plots and marginal posterior distributions of parameters $\beta_{20}$ and $\beta_{21}$ from the ZMPS regression model.

Table 3 presents the posterior parameter estimates and 95% HPDIs from ZMPS fitted model. When obtaining the MCMC samples, the acceptance rate in the RwM algorithm was approximately 32%. Besides, we have computed the number of effectively pseudo-independent draws, that is, the Effective Sample Size (ESS) for each parameter. Figure 2 and Figure 3 depict the chains’ history (trace plots) and the marginal posterior distributions of the regression coefficients. The normality assumption of the generated chains is quite reasonable, even with slight tails on the estimated densities. Additionally, there exists evidence of symmetry since the posterior means and medians are very close to each other. For each parameter, the ESS was estimated at approximately half of *M*, indicating a good mixing of the generated chains without computational waste.

**Figure 4:**
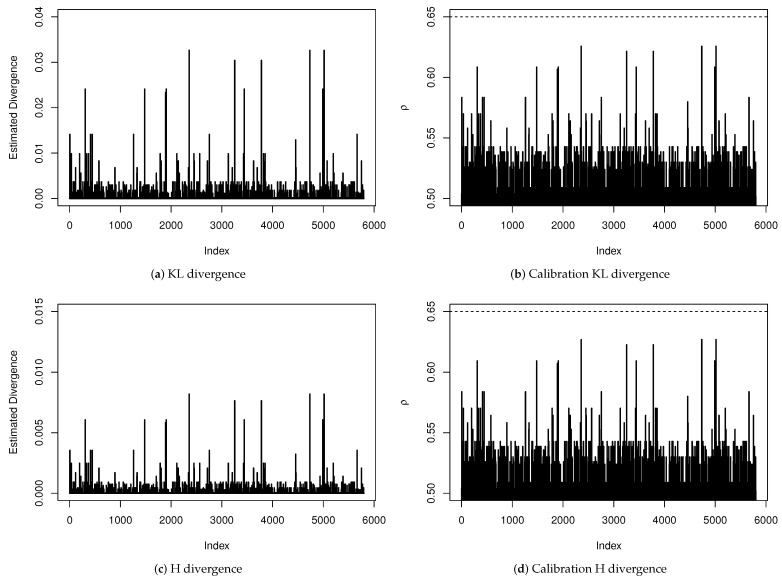
Sensitivity analysis for diagnosis of influential points.

A sensitivity analysis to verify the existence of influential points is presented in Figure 4. We have estimated all divergence measures presented in Table A1 but, since the obtained results led to the same conclusions, we are only reporting the KL and H divergences and their calibration for each observation. Even being very conservative by considering an observation whose distance has a calibration exceeding 0.65 as an influential point, we do not have found evidence that any observation has influenced the estimation of any coefficient of the ZMPS regression model significantly.

For comparison purposes, identical Bayesian procedures were adopted to fit the P, the NB, the PS, the ZMP and the ZMNB regression models. To estimate the fixed dispersion parameter (ϕ) of NB and ZMNB models, we have considered a noninformative inverse-gamma prior distribution with hyperparameters a=b=1.0. For each fitted model, we have estimated the measures presented in Appendix C. The model comparison procedure is summarized in Table 4. One can notice that the zero-modified models have performed considerably better with ZMPS outperforming all. These results are highlighting that the proposed model is highly competitive with well-established models in the literature. This feature can be considered one of the most relevant achievements of the ZMPS model since it has to deal with the positive observations using fewer parameters than, for example, the ZMNB model.

**Figure 5:**
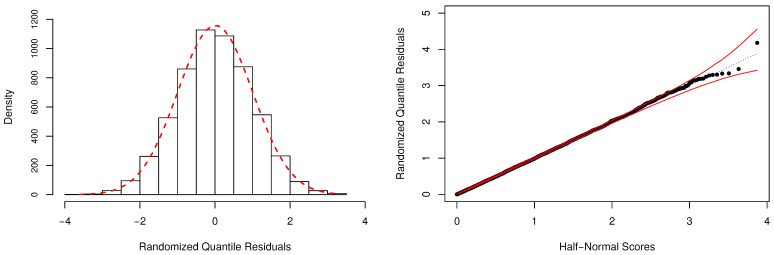
Frequency distribution and half-normal plot with simulated envelope for the randomized quantile residuals (RQRs).

In Table 4, we have also reported the Bayesian *p*-values as a way to evaluate the adequacy of the fitted models. As expected, the P model is unsuitable to describe the considered dataset, and the fit provided by the NB regression model is also highly questionable. For the zero-modified models, there is no indication of overall lack-of-fit, since the posterior values of $p_{{}_{B}}$ were estimated close to 0.50. Figure 5 depicts additional evidence based on the RQRs for validating the fitted ZMPS regression. This residual metric was computed as discussed in Appendix D, using Equation (4). One can notice that the normality assumption of the residuals is easily verified by the behavior of its frequency distribution (left-panel). Additionally, the half-normal probability plot indicates that the fit of the ZMPS model was very satisfactory since all estimated residuals are lying within the simulated envelope (right-panel).

From the results displayed in Table 3, one can make some conclusions. Firstly, we have observed that the HPDIs of parameters $\beta_{11}$ and $\beta_{21}$ do not contain the value zero, which constitutes the dose of ionizing radiation as a relevant covariate to describe the average number of chromosomal aberrations as well the probability of not observing at least one aberration $\left(p_{0}\right)$. For example, the expected number of dicentrics and centric rings in a cell that was exposed to 1.0 Gy is 0.363, and the probability of such aberrations not to occur is ${\hat{p}}_{0}=\Phi \left(1.790-0.319\right)=0.929$. Therefore, based on the posterior estimates, the components of the fitted ZMPS model can be expressed by

$${\hat{\mu }}_{ij}=\exp\left\{-1.481+0.935x_{i}\right\}\;\;\;\;\mathrm{and}\;\;\;\;{\hat{\omega }}_{ij}=\Phi \left(-1.790+1.062x_{i}\right),$$

where $x_{i}$ is the dose of ionizing radiation.

**Figure 6:**
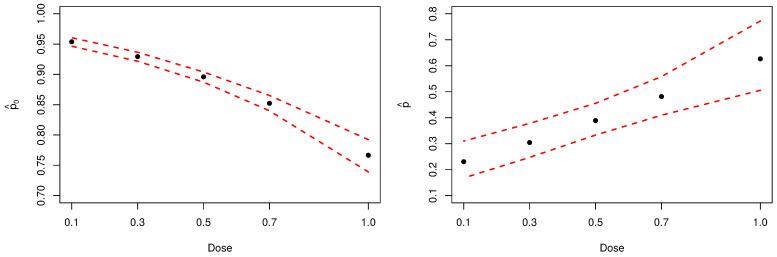
Posterior estimates of parameters $p_{0}$ and *p*. The dashed red lines represent the 95% HPDIs.

Figure 6 present the Bayesian estimates, by dose, for the probability of not observing at least one aberration (left-panel) and for parameter *p* (right-panel). Noticeably, inferences about parameter *p* confirm the initial assumption that the analyzed sample has an excessive amount of zeros.

Table 5 presents a general posterior summary of the models that were fitted to the chromosomal aberration data. Here, parameter λ as estimated as $n^{-1}\sum_{i=1}^{5}\sum_{j=1}^{n_{i}}{\hat{\lambda }}_{ij}$ and $\varsigma^{2}$ was estimated analogously. One can notice that the expected number of zeros $\left({\hat{n}}_{0}\right)$ obtained by the P, the NB and the PS models are slightly lower than the observed $n_{0}$, while those provided by the zero-modified models are very close (or exactly equal) to 5252. Through these measures, one can better understand how the fitted models are adhering to the data since the nature of the observed counts should be well described regarding its overdispersion level and the frequency and the average number of nonzero observations.

**Figure 7:**
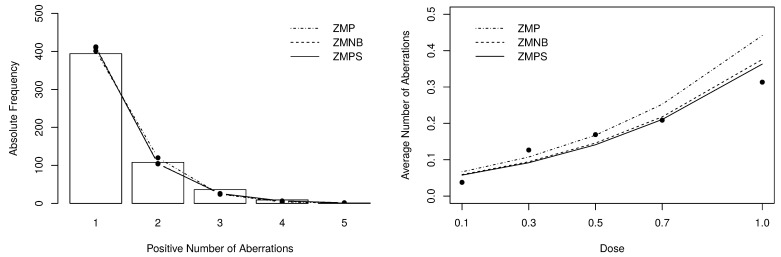
Posterior expected frequencies and dose-response curve fitted by the zero-modified models.

The goodness-of-fit of the fitted models can be evaluated by the $\chi^{2}$ statistic obtained from the observed and expected frequencies. To compute such measure, we have grouped cells with frequencies lower or equal than 5, resulting in 4 degrees of freedom. The obtained statistics are also presented in Table 5. Figure 7 depict the positive expected frequencies (left-panel) and the dose-response curves (right-panel) that were estimated by the zero-modified models. Noticeably, the zero-modified models describe much better the data’s behavior, especially the ZMNB and the ZMPS distributions.

From the obtained results, one can conclude that despite the suitable fit provided by the ZMNB regression model, the proposed model have adhered better to the chromosomal aberration data. This achievement can be regarded as extremely relevant since the ZMNB model has an additional (dispersion) parameter to handle the non-zero observations. In contrast, the proposed model was proved highly competitive by its ability to accommodate the data overdispersion and zero modification using fewer parameters.

## 6. Concluding Remarks

This work aimed to introduce the ZMPS regression model as an alternative for the analysis of overdispersed datasets exhibiting zero-modification in the presence of covariates. Intensive Monte Carlo simulation studies were performed, and the obtained results have allowed us to assess the empirical properties of the Bayesian estimators and then conclude about the suitability of the adopted methodology to the predefined scenarios. The proposed model was considered for analyzing a real dataset on the number of cytogenetic chromosomal aberrations, considering the dose of ionizing radiation as the covariate for both model components. The response variable was identified as overdispersed and heavily zero-inflated, which justified using the ZMPS regression model. The main conclusion one can make from the fitted models is that the dose is statistically relevant to describe either the probability of occurrence and the average incidence of aberrations. Besides, when looking at the $\chi^{2}$ statistic and the posterior-based comparison criteria, we have noticed that the proposed model has presented a better fit when compared to its competitors and therefore, it can be considered an excellent addition to the set of models that can be used for the analysis of overdispersed and zero-modified count data.

## Figures and Tables

**Table 1:** Actual parameter values for simulation of zero-modified artificial datasets.

Case	Scenario	Link	$\beta_{10}$	$\beta_{11}$	$\beta_{20}$	$\beta_{21}$	Range	Range
							$\mu_{i}$	$p_{i}$
I	1	Logit Probit CLL	1.50	3.00	−1.00	−1.00	$\left(4.48;90.02\right)$	$\left(0.12;0.30\right)$ $\left(0.02;0.18\right)$ $\left(0.13;0.34\right)$
	2	Logit Probit CLL	1.50	3.00	−1.00	0.50	$\left(4.48;90.02\right)$	$\left(0.30;0.38\right)$ $\left(0.18;0.31\right)$ $\left(0.34;0.45\right)$
	3	Logit Probit CLL	1.50	−1.50	−1.00	−1.00	$\left(1.00;4.48\right)$	$\left(0.23;0.30\right)$ $\left(0.04;0.18\right)$ $\left(0.24;0.34\right)$
	4	Logit Probit CLL	1.50	−1.50	−1.00	0.50	$\left(1.00;4.48\right)$	$\left(0.30;0.73\right)$ $\left(0.18;0.59\right)$ $\left(0.34;0.88\right)$
D	1	Logit	−1.00	1.00	0.50	0.50	$\left(0.37;1.00\right)$	$\left(1.41;2.30\right)$
		Probit						$\left(1.62;2.56\right)$
		CLL						$\left(1.80;2.99\right)$
	2	Logit	−1.00	1.00	1.50	−1.00	$\left(0.37;1.00\right)$	$\left(1.20;3.02\right)$
		Probit						$\left(1.33;3.45\right)$
		CLL						$\left(1.56;3.66\right)$
	3	Logit	−1.00	−1.50	0.50	0.50	$\left(0.08;0.37\right)$	$\left(2.30;9.64\right)$
		Probit						$\left(2.56;11.09\right)$
		CLL						$\left(2.99;12.31\right)$
	4	Logit	−1.00	−1.50	1.50	−1.00	$\left(0.08;0.37\right)$	$\left(3.02;8.21\right)$
		Probit						$\left(3.45;9.12\right)$
		CLL						$\left(3.66;10.65\right)$

I: inflation; D: deflation; and CLL: complementary log–log.

**Table 2:** Descriptive summary of the numbers of dicentrics and centric ring aberrations.

$x_{i}$	$y_{\mathrm{ij}}$						$n_{i}$	${\bar{y}}_{i}$	$s_{i}$	$s_{i}^{2}/{\bar{y}}_{i}$
	0	1	2	3	4	5				
0.1	2130	59	9	2	0	0	2200	0.038	0.224	1.316
0.3	1088	84	19	6	3	0	1200	0.127	0.449	1.591
0.5	875	88	30	7	0	0	1000	0.169	0.493	1.438
0.7	679	88	23	8	1	1	800	0.209	0.568	1.545
1.0	480	75	27	13	5	0	600	0.313	0.732	1.712

**Table 3:** Posterior parameter estimates and 95% highest posterior density intervals (HPDIs) from ZMPS fitted model.

Parameter	Mean	Median	Std. Dev.	ESS	95% HPDI	
					Lower	Upper
$\beta_{10}$	−1.481	−1.479	0.192	1874.876	−1.868	−1.119
$\beta_{11}$	−0.935	−0.937	0.279	1912.372	−0.411	−1.497
$\beta_{20}$	−1.790	−1.789	0.044	1834.592	−1.873	−1.706
$\beta_{21}$	−1.062	−1.063	0.074	1910.648	−0.924	−1.211

**Table 4:** Comparison criteria and adequacy measures for the fitted models.

Model	DIC	EAIC	EBIC	NLMPL	$p_{{}_{B}}$
P	4650.631	4652.624	4665.955	2325.750	1.000
NB	4340.938	4343.915	4363.912	2170.321	0.936
PS	4436.313	4438.312	4451.643	2218.355	0.578
ZMP	4323.300	4327.164	4353.826	2161.734	0.516
ZMNB	4321.668	4326.960	4360.288	2160.530	0.598
ZMPS	4320.539	4324.549	4351.212	2160.138	0.542

**Table 5:** Posterior parameter estimates and goodness-of-fit evaluation.

Model	Parameter	$\hat{\lambda }$	${\hat{\varsigma }}^{2}$	${\hat{n}}_{0}$	$\chi^{2}$	*p*-Value
P	${\hat{\beta }}_{10}=-2.97$	0.131	0.131	5086	2343.773	<0.001
	β^11=−1.95					
NB	${\hat{\beta }}_{10}=-3.02$	0.133	0.232	5202	20.050	<0.001
	β^11=−2.07					
	ϕ^−=−0.28					
PS	${\hat{\beta }}_{10}=-2.99$	0.132	0.157	5126	266.458	<0.001
	β^11=−1.98					
ZMP	${\hat{\beta }}_{10}=-0.86$	0.132	0.199	5251	16.456	0.002
	β^11=−0.82					
	${\hat{\beta }}_{20}=-1.79$					
	β^21=−1.06					
ZMNB	${\hat{\beta }}_{10}=-1.33$	0.131	0.206	5251	7.255	0.123
	β^11=−0.88					
	${\hat{\beta }}_{20}=-1.79$					
	β^21=−1.07					
	ϕ^−=−1.51					
ZMPS	Table 3	0.132	0.210	5252	5.298	0.258

## Data Availability

The dataset analyzed in this work was made available in the Supplementary Material.

## Supplementary Materials

The following are available online at https://www.mdpi.com/article/10.3390/e23060646/s1.

## Abbreviations

   The following abbreviations are used in this manuscript:

ANCP	Above noncoverage probability
B	Bias
BNCP	Below noncoverage probability
CDF	Cumulative distribution function
CP	Coverage probability
CPO	Conditional predictive ordinate
CS	Chi-square
DIC	Deviance information criterion
EAIC	Expected Akaike information criterion
EBIC	Expected Bayesian information criterion
ESS	Effective sample size
GLM	Generalized linear model
H	Hellinger
HPDI	Highest posterior density interval
J	Jeffrey
KL	Kullback–Leibler
L${}^{1}$	Variational divergence
LMPL	Log-marginal pseudo-likelihood
MAPE	Mean absolute percentage error
MC	Monte Carlo
MCMC	Markov chain Monte Carlo
MSE	Mean squared error
NB	Negative binomial
NI	Number of iterations
P	Poisson
PMF	Probability mass function
PS	Poisson–Sujatha
RQR	Randomized quantile residuals
RwM	Random-walk metropolis
ZIP	Zero-inflated Poisson
ZMP	Zero-modified Poisson
ZMPS	Zero-modified Poisson–Sujatha
ZTP	Zero-truncated Poisson
ZTPS	Zero-truncated Poisson–Sujatha

## Appendix A. Algorithms

### Appendix A.1. Random-Walk Metropolis

**Algorithm A1** Random-walk metropolis.
1: **procedure**RwM($N,\beta_{1}^{\left(0\right)},\beta_{2}^{\left(0\right)}$) 2: Set t←1 and $\nu \leftarrow \;n{\left(n+1\right)}^{-1}$ 3: **while** t⩽N **do** 4: Generate $\psi_{1}∼N_{{\bar{q}}_{1}}\left[\nu \beta_{1}^{\left(t-1\right)},\nu S_{1}^{\left(t-1\right)}\right]$ and $\psi_{2}∼N_{{\bar{q}}_{2}}\left[\nu \beta_{2}^{\left(t-1\right)},\nu S_{2}^{\left(t-1\right)}\right]$ 5: Set $\alpha_{1}\leftarrow \;\exp\left\{\pi_{1}\left(\psi_{1};y\right)-\pi_{1}\left(\beta_{1}^{\left(t-1\right)};y\right)\right\}$ 6: Set $\alpha_{2}\leftarrow \;\exp\left\{\pi_{2}\left(\psi_{2};y\right)-\pi_{2}\left(\beta_{2}^{\left(t-1\right)};y\right)\right\}$ 7: Set $\beta_{1}^{\left(t\right)}\leftarrow \;\beta_{1}^{\left(t-1\right)}$ and $\beta_{2}^{\left(t\right)}\leftarrow \;\beta_{2}^{\left(t-1\right)}$ 8: Generate $u_{1},u_{2}∼\;U\left(0,1\right)$ 9: **if** $u_{1}⩽\min\left\{1,\alpha_{1}\right\}$ and $u_{2}⩽\min\left\{1,\alpha_{2}\right\}$ **then** 10: Set $\beta_{1}^{\left(t\right)}\leftarrow \;\psi_{1}$ and $\beta_{2}^{\left(t\right)}\leftarrow \;\psi_{2}$ 11: **end if** 12: Set t←t+1 13: **end while** 14: **return** ${\left\{\beta^{t}\right\}}_{t=1}^{N}={\left\{\beta_{1}^{t},\beta_{2}^{t}\right\}}_{t=1}^{N}$ 15: **end procedure**

### Appendix A.2. Sequential Search

**Algorithm A2** Sequential search.
1: **procedure**SeqSea($\beta_{10},\beta_{11},\beta_{20},\beta_{21}$) 2: Generate $x,u∼\;U\left(0,1\right)$ 3: Set $\mu \leftarrow \;\exp\{\beta_{10}+\beta_{11}x\}$ and $\omega \leftarrow \;g_{2}^{-1}\;\left(\beta_{20}+\beta_{21}x\right)$ 4: Set $k\leftarrow \;\left(1-\omega \right)$ and y←0 5: **while** u>k **do** 6: Set y←y+1 and $k\leftarrow \;k+\omega \;P_{*}\;\left(Y=y;\mu \right)$ 7: **end while** 8: **return** *y* 9: **end procedure**

## Appendix B. Influential Points

Identifying influential observations is a crucial step in any statistical analysis. Usually, the presence of influential points impacts the inferential procedures and the subsequent conclusions considerably. In this way, this subsection is dedicated to present some case deletion Bayesian diagnostic measures that can be used to quantify the influence of observations from each subject in a given dataset.

The computation of divergence measures between posterior distributions is a useful way to quantify influence. According [66], the φ-divergence measure between two densities *f* and *g* for θ∈Θ is defined by

$$d_{\varphi }=\int_{\Theta }g\left(\theta \right)\varphi \;\left[\frac{f\left(\theta \right)}{g\left(\theta \right)}\right]\;d\theta ,$$

where φ is a smooth convex, lower semicontinuous function such that φ(1)=0. Some popular divergence measures can be obtained by choosing specific functions for φ. The well-known Kullback–Leibler (KL) divergence is obtained by considering φ(z)=−log(z). A symmetric version of the KL divergence, the Jeffrey (J) divergence, can be obtained by specifying φ(z)=(z−1)log(z) and the variational divergence ($L^{1}$ norm) is obtained when φ(z)=0.50|z−1|. In addition, the Chi-square (CS) divergence is obtained by considering $\varphi \left(z\right)={\left(z-1\right)}^{2}$ and the Hellinger (H) distance arises when $\varphi \left(z\right)=0.50{\left(\sqrt{z}-1\right)}^{2}$. We refer to [67] for a detailed study on several types of φ-divergence.

Let $g\left(\beta \right)=\pi \left(\beta ;y_{i}\right)$ be the joint posterior distribution of β based only on the *i*-th observation and let $f\left(\beta \right)=\pi \left(\beta ;y_{i}^{j}\right)$, where $y_{-i}\;=\left(y_{1},\cdots ,y_{i-1},y_{i+1},\cdots ,y_{n}\right)$ is the response vector without the *i*-th observation. After some algebra (see [68] for the KL divergence case), one can verify that the φ-divergence corresponds to

$$d_{\varphi }=E_{\beta }\;\left\{\varphi \;\left[\frac{E_{\beta }\;{\left[P_{*}\;{\left(Y_{i}=y_{i};\beta \right)}^{-1}\;;y\right]}^{-1}}{P_{*}\;\left(Y_{i}=y_{i};\beta \right)}\right]\;;y\right\},$$

where $E_{\beta }{\left[P_{*}{\left(Y_{i}=y_{i};\beta \right)}^{-1};y\right]}^{-1}$ is the conditional predictive ordinate (CPO) statistic [69] for the *i*-th observation. Here, we are also not able to compute the inner expectation over β analytically and so, an MC estimator for the ${\mathrm{CPO}}_{i}$ is given by

(A1) $${\hat{\mathrm{CPO}}}_{i}={\left[\frac{1}{M}\sum_{t=1}^{M}P_{*}\;{\left(Y_{i}=y_{i};\beta^{\left(t\right)}\right)}^{-1}\right]}^{-1}.$$

According to [70], the harmonic mean estimator (A1) is stable when most of the individual log-likelihood values exceed -10. Using the estimated CPO, one can approximate the local influence of a particular $y_{i}$ on the joint posterior distribution (12) as

$${\hat{d}}_{\varphi }=\frac{1}{M}\sum_{t=1}^{M}\varphi \;\left[\frac{{\hat{\mathrm{CPO}}}_{i}}{P_{*}\;\left(Y_{i}=y_{i};\beta^{\left(t\right)}\right)}\right].$$

One can notice that, if $\pi \left(\beta ;y_{-i}\right)=\pi \left(\beta ;y\right)$, then there is no divergence caused by observation $y_{i}$. In practice, however, it may not be elementary to define a threshold value for the divergence to decide about the magnitude of the influence [71]. A measure of calibration for the KL divergence was proposed by [72]. The idea is based on the typical toy binary example of tossing a coin once and observing its upper face. This experiment can be described by $P\left(Y=y;\rho \right)=\rho^{y}{\left(1-\rho \right)}^{1-y}$, y∈{0,1}, where ρ∈[0,1] is the probability of success. Regardless of what success means, if the coin is unbiased, then P(Y=y;ρ)=0.50. Thus, the φ-divergence between a (possibly) biased and an unbiased coin is given by

$$d_{\varphi }\;\left(\rho \right)=\frac{\varphi \left(2\rho \right)+\varphi \left[2\left(1-\rho \right)\right]}{2},$$

from which one can conclude that the divergence between two posteriors distributions can be associated with the biasedness of a coin [67]. By analogy, this implies that predict unobserved responses using $\pi (\beta ;y_{-i})$ instead of π(β;y) is equivalent to describe an unobserved event as having probability $\rho_{i}$, when the correct probability is 0.50. Considering some specific choices for φ, in Table A1 we present MC estimators that can be used to compute the local influence of each $y_{i}$. Besides, we also present the expression of $d_{\varphi }\left(\rho \right)$ for each φ. For ease of notation, we assume $f_{i}^{t}=P\left(Y_{i}=y_{i};\beta^{\left(t\right)}\right)$.

**Table A1:** MC estimators for some φ-divergence measures and their calibration.

φ	${\hat{d}}_{\varphi }$	$d_{\varphi }\left(\rho \right)$	${\hat{\rho }}_{\varphi }$
${\mathrm{KL}}_{i}$	$\frac{1}{M}\sum_{t=1}^{M}\log\left(f_{i}^{t}\right)-\log\left({\hat{\mathrm{CPO}}}_{i}\right)$	$-\frac{1}{2}\log\left[4\rho_{i}\left(1-\rho_{i}\right)\right]$	$\frac{1}{2}\left[1+\sqrt{1-e^{-2{\hat{d}}_{i}}}\right]$
$J_{i}$	$\frac{1}{M}\sum_{t=1}^{M}\left(\frac{{\hat{\mathrm{CPO}}}_{i}}{f_{i}^{t}}-1\right)\log\left(\frac{{\hat{\mathrm{CPO}}}_{i}}{f_{i}^{t}}\right)$	$-\frac{\left(1-2\rho_{i}\right)}{2}\log\left(\frac{\rho_{i}}{1-\rho_{i}}\right)$	no closed-form
$L_{i}^{1}$	$\frac{1}{2M}\sum_{t=1}^{M}\frac{\left|{\hat{\mathrm{CPO}}}_{i}-f_{i}^{t}\right|}{f_{i}^{t}}$	$\frac{1}{2}\left|1-2\rho_{i}\right|$	$\frac{1}{2}+{\hat{d}}_{i}$
${\mathrm{CS}}_{i}$	$\frac{1}{M}\sum_{t=1}^{M}\frac{{\left({\hat{\mathrm{CPO}}}_{i}-f_{i}^{t}\right)}^{2}}{{\left(f_{i}^{t}\right)}^{2}}$	${\left(1-2\rho_{i}\right)}^{2}$	$\frac{1}{2}\left[1+\sqrt{{\hat{d}}_{i}}\right]$
$H_{i}$	$\frac{1}{2M}\sum_{t=1}^{M}{\left(\sqrt{\frac{{\hat{\mathrm{CPO}}}_{i}}{f_{i}^{t}}}-1\right)}^{2}$	$1-\frac{1}{\sqrt{2}}\;\left(\sqrt{\rho_{i}}+\sqrt{1-\rho_{i}}\right)$	$\frac{1}{2}+\left[\sqrt{{\hat{d}}_{i}}-\sqrt{{\hat{d}}_{i}^{3}}\right]\sqrt{2-{\hat{d}}_{i}}$

KL: K; J: Jeffrey; $L^{1}$: Variational; CS: Chi-Square; and H: Hellinger.

The function $d_{\varphi }\left(\rho \right)$ is symmetric about 0.50 and increases as ρ moves away from 0.50. In addition, $\inf_{\rho \in (0,1)}d_{\varphi }\left(\rho \right)=0$, which is attained at ρ=0.50 since $d_{\varphi }\left(0.50\right)=\varphi \left(1\right)=0$. Therefore, a general measure of calibration based on the φ-divergence can be obtained by solving

$$2d_{\varphi }\left(\rho \right)-\varphi \left(2\rho \right)-\varphi \left[2\left(1-\rho \right)\right]=0.$$

An estimator for the calibration measure $\left(\rho_{\varphi }\right)$ associated with each φ-divergence type is also presented in Table A1. Clearly, depending on the form of φ, such an equation may not have a closed-form, which is the case of the J divergence. Besides, one can notice that $\rho_{i}\in \left[0.50,1\right]$ and so, for $\rho_{i}\gg 0.50$, the *i*-th observation may be considered an influential point. For example, if $\rho_{i}>0.80$ is considered a significative bias, then $y_{i}$ will be classified as influential if ${\hat{d}}_{i}>0.223$$\left(d_{\varphi }\left(0.80\right)\approx 0.223\right)$ under the KL divergence or yet if ${\hat{d}}_{i}>0.051$$\left(d_{\varphi }\left(0.80\right)\approx 0.051\right)$ under the H divergence.

## Appendix C. Model Comparison and Adequacy

There are several techniques for Bayesian model selection that are useful to compare competing models. The most popular method is the deviance information criterion (DIC), which was proposed to work simultaneously to measure fit and complexity of the model. The DIC criterion is defined as

$$\mathrm{DIC}=E_{\beta }\;\left[D\left(\beta \right)\right]+\varrho_{{}_{D}}=\underline{D}\;\left(\beta \right)+\varrho_{{}_{D}},$$

where D(β)=−2ℓ(β;y) is the deviance function and $\varrho_{{}_{D}}=\underline{D}\left(\beta \right)-D\left(\hat{\beta }\right)$ is the effective number of model parameters, with $\hat{\beta }$ given by (14). A negative value for $\varrho_{{}_{D}}$ may suggest that the log-likelihood function is non-concave, the prior distribution is misspecified, or the posterior expected value is not a good estimator for β. On the other hand, when $\varrho_{{}_{D}}\gg d$, then there is an indication of overfitting with estimate $\hat{\beta }$.

Noticeably, we are not able to compute the expectation of D(β) over β analytically. In this case, an MC estimator for such a measure is given by

$$\hat{\underline{D}}\;\left(\beta \right)=-\frac{2}{M}\sum_{t=1}^{M}\ell \;\left(\beta^{\left(t\right)};y\right),$$

and so the DIC can be estimated by $\hat{\mathrm{DIC}}=2\hat{\underline{D}}\left(\beta \right)-D\left(\hat{\beta }\right)$.

The expected Akaike (EAIC) and the expected Bayesian (EBIC) information criteria can also be used when comparing Bayesian models [73,74]. Using the approximation for the expected value of D(β), these measures can be estimated by

$$\hat{\mathrm{EAIC}}=\hat{\underline{D}}\;\left(\beta \right)+2d\;\;\;\;\mathrm{and}\;\;\;\;\hat{\mathrm{EBIC}}=\hat{\underline{D}}\;\left(\beta \right)+d\log\left(n\right).$$

Another widely used criterion is derived from the CPO statistic, which is based on the cross-validation criterion to compare models. For the *i*-th observation, the CPO can be estimated through Equation (A1). A summary statistic of the estimated CPO’s is the log-marginal pseudo-likelihood (LMPL) given by the sum of the logarithms of ${\hat{\mathrm{CPO}}}_{i}$’s. Regarding model comparison, we have that the lower the values of DIC, EAIC, EBIC, and NLMPL (negative LMPL), the better the fit.

In addition to comparing, researchers are often interested in verifying the adequacy of the fitted models. An effective way to evaluate model suitability is based on the use of measures derived from the ppd. For instance, if any observation is extremely unlikely relative to the ppd, the obtained fit’s adequacy might be questionable. Ref. [75] proposed a widespread discrepancy measure between model and data. In our case, we need a slightly adapted version of such a measure, which is given by

$$T\left(y,\beta \right)=-2\sum_{i=1}^{n}\log\left[P_{*}\;\left(Y_{i}=y_{i};\beta \right)\right].$$

The Bayesian *p*-value (posterior predictive *p*-value), proposed by [76], is defined as

$$p_{{}_{B}}=P\left[T\left(y_{r},\beta \right)⩾T\left(y,\beta \right);y\right],$$

where $y_{r}$ denotes the response vector that might have been observed if the conditions generating y were reproduced. This predictive measure can be empirically estimated as the relative number of times that $T(y_{r},\hat{\beta })$ exceeds $T(y,\hat{\beta })$ out of *B* simulations. In general, the model fit becomes suspect if the discrepancy is of practical relevance, and the associated Bayesian *p*-value is close either to 0 or 1 [75]. A large (small) value of $p_{{}_{B}}$, say greater than 0.95 (lower than 0.05), indicates model misspecification (lack-of-fit), that is, the observed behavior would be unlikely to be seen if we replicate the response vector using the fitted model.

## Appendix D. Residual Analysis

The residual analysis plays an essential role in the task of validating the results obtained from a regression model. In general, residual metrics are responsible for indicating departures from the underlying model assumptions by quantifying the portion of data variability that the fitted model is not explaining. Assessing a regression model’s adequacy using residual metrics is a common practice nowadays due to the availability of statistical packages providing diagnostic tools for well-established models. However, deriving appropriate residuals is not always an easy task for non-normal models that accommodate overdispersion. In this way, we will consider a popular residual metric proposed by [77], the randomized quantile residuals (RQRs), which can be straightforwardly used in our context to assess the appropriateness of the proposed model when fitted to real data.

For obvious reasons, we focus on the definition of RQRs for discrete random variables. In this case, the RQR associated with the *i*-th observation is defined as $r_{i}=\Phi^{-1}\left(u_{i}\right)$, where Φ denotes the cdf of the standard normal distribution and $u_{i}$ is a Uniform random variable defined on $\left(a_{i},b_{i}\right]$, with $a_{i}=\lim_{y↑y_{i}}F\left(y_{i}\right)$ and $b_{i}=F\left(y_{i}\right)$, where $F(y_{i})$ is the cdf of the current model. In our case, we may obtain an MC estimator for the RQR as ${\hat{r}}_{i}=\Phi^{-1}\left(u_{i}\right)$, with $u_{i}∼U\left(\lim_{y↑y_{i}}{\hat{F}}_{*}\left(y_{i}\right),{\hat{F}}_{*}\left(y_{i}\right)\right]$. Here, ${\hat{F}}_{*}\left(y_{i}\right)\equiv F_{*}\left(y_{i};{\hat{\mu }}_{i},{\hat{\omega }}_{i}\right)$ is an estimate for the probability of $Y_{i}⩽y_{i}$ using cdf (4), where ${\hat{\mu }}_{i}$ and ${\hat{\omega }}_{i}$ depend on the fitted model as ${\hat{\mu }}_{i}=\log\left(x_{1i}^{⊺}{\hat{\beta }}_{1}\right)$ and ${\hat{\omega }}_{i}=g_{2}^{-1}\left(x_{2i}^{⊺}{\hat{\beta }}_{2}\right)$.

The primary assumption for this metric is that ${\hat{r}}_{i}∼N\left(0,1\right)$ must hold, whichever the variability degree of ${\hat{\mu }}_{i}$ and ${\hat{\omega }}_{i}$. In this case, after model fitting, one has to evaluate if these residuals are normally distributed around zero, which can be made through adherence tests and by using graphical techniques as histograms and half-normal probability plots. An excellent alternative for checking whether RQRs are consistent with the fitted model is the inclusion of simulated envelopes in their half-normal plot. Thus, if a significant subset of estimated residuals falls outside the envelope bands, then the fitted model’s adequacy must be questioned, and further investigation on the corresponding observations is necessary. Ref. [78] provides an algorithm for obtaining simulated envelopes for a half-normal plot.
