# Supplementary material for: A New Regression Model for the Analysis of Overdispersed and Zero-Modified Count Data
Source: Entropy (Basel). 2021 May 21;23(6):646. doi: 10.3390/e23060646 (PMC8224290; doi:10.3390/e23060646)
Supplement: Supplementary file 1 [file entropy-23-00646-s001.zip › Supplementary_Material/Simulation_Results/simulation_results.pdf]

# Supplementary Material

# A New Regression Model for the Analysis of Overdispersed and Zero-modified Count Data

Wesley Bertoli, Katiane S. Conceição,
Marinho G. Andrade and Francisco Louzada

**Abstract**

This supplementary material contains all the results obtained from the Monte Carlo simulation study described in Section 4 of the manuscript. In all Figures, the straight blue lines represent the actual values, and the 95% HPDIs are represented by the dashed red lines. The filled black dots represent the estimated values for each generated observation.

# 1 Zero-inflated artificial data

## 1.1 Using *logit* link function

Table 1: Empirical properties of the Bayesian estimators using zero-inflated artificial samples (Scenarios 1 and 2).

| $n$ | Parameter | Bias | MSE | $\sqrt{\frac{\text{MSE}}{\text{Var}}}$ | MAPE (%) |
|---|---|---|---|---|---|
| | | | Scenario 1 | | |
| 50 | $\beta_{10}$ | −0.116 | 0.336 | 1.020 | 29.127 |
| | $\beta_{11}$ | −0.527 | 1.784 | 1.088 | 30.652 |
| | $\beta_{20}$ | 0.119 | 0.329 | 1.022 | 45.714 |
| | $\beta_{21}$ | 0.116 | 0.990 | 1.007 | 78.781 |
| 100 | $\beta_{10}$ | −0.097 | 0.161 | 1.031 | 20.605 |
| | $\beta_{11}$ | −0.138 | 0.480 | 1.020 | 18.120 |
| | $\beta_{20}$ | 0.084 | 0.247 | 1.014 | 40.288 |
| | $\beta_{21}$ | 0.053 | 0.774 | 1.002 | 70.962 |
| 200 | $\beta_{10}$ | −0.033 | 0.058 | 1.010 | 12.471 |
| | $\beta_{11}$ | −0.098 | 0.193 | 1.026 | 11.590 |
| | $\beta_{20}$ | 0.012 | 0.110 | 1.001 | 26.005 |
| | $\beta_{21}$ | 0.061 | 0.412 | 1.004 | 50.919 |
| 500 | $\beta_{10}$ | −0.032 | 0.022 | 1.024 | 7.870 |
| | $\beta_{11}$ | −0.011 | 0.064 | 1.001 | 6.794 |
| | $\beta_{20}$ | 0.009 | 0.049 | 1.001 | 17.633 |
| | $\beta_{21}$ | 0.023 | 0.169 | 1.002 | 33.136 |
| | | | Scenario 2 | | |
| 50 | $\beta_{10}$ | −0.164 | 0.180 | 1.085 | 21.689 |
| | $\beta_{11}$ | −0.136 | 0.382 | 1.025 | 16.319 |
| | $\beta_{20}$ | 0.128 | 0.328 | 1.026 | 45.883 |
| | $\beta_{21}$ | −0.081 | 0.896 | 1.004 | 151.990 |
| 100 | $\beta_{10}$ | −0.078 | 0.094 | 1.034 | 15.908 |
| | $\beta_{11}$ | −0.075 | 0.204 | 1.014 | 11.916 |
| | $\beta_{20}$ | 0.049 | 0.217 | 1.006 | 36.781 |
| | $\beta_{21}$ | −0.016 | 0.574 | 1.000 | 117.390 |
| 200 | $\beta_{10}$ | −0.051 | 0.044 | 1.031 | 11.079 |
| | $\beta_{11}$ | −0.010 | 0.104 | 1.000 | 8.443 |
| | $\beta_{20}$ | 0.010 | 0.101 | 1.000 | 25.223 |
| | $\beta_{21}$ | 0.013 | 0.322 | 1.000 | 90.972 |
| 500 | $\beta_{10}$ | −0.024 | 0.015 | 1.019 | 6.533 |
| | $\beta_{11}$ | −0.005 | 0.033 | 1.000 | 4.778 |
| | $\beta_{20}$ | 0.005 | 0.039 | 1.000 | 15.802 |
| | $\beta_{21}$ | 0.002 | 0.104 | 1.000 | 52.292 |

Table 2: Empirical properties of the Bayesian estimators using zero-inflated artificial samples (Scenarios 3 and 4).

| $n$ | Parameter | Bias | MSE | $\sqrt{\frac{\text{MSE}}{\text{Var}}}$ | MAPE (%) |
|---|---|---|---|---|---|
| Scenario 3 | | | | | |
| 50 | $\beta_{10}$ | −0.338 | 0.584 | 1.115 | 37.493 |
| | $\beta_{11}$ | 0.281 | 1.758 | 1.023 | 69.019 |
| | $\beta_{20}$ | 0.109 | 0.388 | 1.016 | 49.700 |
| | $\beta_{21}$ | 0.186 | 1.102 | 1.016 | 84.008 |
| 100 | $\beta_{10}$ | −0.120 | 0.241 | 1.032 | 24.811 |
| | $\beta_{11}$ | −0.036 | 1.045 | 1.001 | 52.507 |
| | $\beta_{20}$ | 0.056 | 0.222 | 1.007 | 38.480 |
| | $\beta_{21}$ | 0.104 | 0.681 | 1.008 | 67.186 |
| 200 | $\beta_{10}$ | −0.071 | 0.105 | 1.025 | 16.547 |
| | $\beta_{11}$ | −0.005 | 0.526 | 1.000 | 38.617 |
| | $\beta_{20}$ | 0.006 | 0.106 | 1.000 | 25.932 |
| | $\beta_{21}$ | 0.066 | 0.405 | 1.005 | 50.259 |
| 500 | $\beta_{10}$ | −0.024 | 0.039 | 1.008 | 10.202 |
| | $\beta_{11}$ | −0.002 | 0.171 | 1.000 | 21.413 |
| | $\beta_{20}$ | 0.005 | 0.051 | 1.000 | 17.858 |
| | $\beta_{21}$ | 0.043 | 0.166 | 1.006 | 32.488 |
| Scenario 4 | | | | | |
| 50 | $\beta_{10}$ | −0.221 | 0.386 | 1.070 | 31.170 |
| | $\beta_{11}$ | 0.138 | 1.024 | 1.009 | 54.128 |
| | $\beta_{20}$ | 0.086 | 0.380 | 1.010 | 48.477 |
| | $\beta_{21}$ | −0.063 | 1.108 | 1.002 | 165.519 |
| 100 | $\beta_{10}$ | −0.114 | 0.192 | 1.035 | 23.082 |
| | $\beta_{11}$ | 0.062 | 0.584 | 1.003 | 41.147 |
| | $\beta_{20}$ | 0.068 | 0.218 | 1.011 | 37.185 |
| | $\beta_{21}$ | −0.076 | 0.700 | 1.004 | 133.345 |
| 200 | $\beta_{10}$ | −0.052 | 0.067 | 1.021 | 13.693 |
| | $\beta_{11}$ | 0.011 | 0.236 | 1.000 | 26.593 |
| | $\beta_{20}$ | 0.040 | 0.080 | 1.010 | 22.414 |
| | $\beta_{21}$ | −0.033 | 0.269 | 1.002 | 81.162 |
| 500 | $\beta_{10}$ | −0.025 | 0.028 | 1.012 | 8.884 |
| | $\beta_{11}$ | 0.022 | 0.093 | 1.002 | 16.306 |
| | $\beta_{20}$ | −0.001 | 0.042 | 1.000 | 16.202 |
| | $\beta_{21}$ | 0.010 | 0.118 | 1.000 | 54.116 |

Table 3: *Posterior* estimates for model parameters using zero-inflated artificial samples (Scenarios 1 and 2).

| $n$ | Parameter | Mean | Median | Std. Dev. | 95% HPDI | |
|---|---|---|---|---|---|---|
| | | | | | Lower | Upper |
| Scenario 1 | | | | | | |
| 50 | $\beta_{10}$ | 1.384 | 1.386 | 0.568 | 0.416 | 2.348 |
| | $\beta_{11}$ | 2.473 | 2.461 | 1.228 | 0.736 | 4.230 |
| | $\beta_{20}$ | −0.881 | −0.865 | 0.561 | −2.184 | 0.395 |
| | $\beta_{21}$ | −0.884 | −0.873 | 0.988 | −3.221 | 1.432 |
| 100 | $\beta_{10}$ | 1.403 | 1.403 | 0.390 | 0.701 | 2.105 |
| | $\beta_{11}$ | 2.862 | 2.855 | 0.679 | 1.631 | 4.105 |
| | $\beta_{20}$ | −0.916 | −0.907 | 0.490 | −1.932 | 0.080 |
| | $\beta_{21}$ | −0.947 | −0.942 | 0.878 | −2.784 | 0.878 |
| 200 | $\beta_{10}$ | 1.467 | 1.467 | 0.238 | 1.031 | 1.903 |
| | $\beta_{11}$ | 2.902 | 2.897 | 0.428 | 2.089 | 3.721 |
| | $\beta_{20}$ | −0.988 | −0.983 | 0.332 | −1.635 | −0.346 |
| | $\beta_{21}$ | −0.939 | −0.933 | 0.639 | −2.219 | 0.332 |
| 500 | $\beta_{10}$ | 1.468 | 1.468 | 0.147 | 1.188 | 1.748 |
| | $\beta_{11}$ | 2.989 | 2.987 | 0.253 | 2.505 | 3.476 |
| | $\beta_{20}$ | −0.991 | −0.988 | 0.220 | −1.414 | −0.571 |
| | $\beta_{21}$ | −0.977 | −0.975 | 0.410 | −1.758 | −0.200 |
| Scenario 2 | | | | | | |
| 50 | $\beta_{10}$ | 1.336 | 1.336 | 0.391 | 0.588 | 2.087 |
| | $\beta_{11}$ | 2.864 | 2.858 | 0.603 | 1.725 | 4.011 |
| | $\beta_{20}$ | −0.872 | −0.860 | 0.558 | −2.071 | 0.308 |
| | $\beta_{21}$ | 0.419 | 0.416 | 0.943 | −1.571 | 2.415 |
| 100 | $\beta_{10}$ | 1.422 | 1.422 | 0.296 | 0.849 | 1.997 |
| | $\beta_{11}$ | 2.925 | 2.922 | 0.445 | 2.023 | 3.828 |
| | $\beta_{20}$ | −0.951 | −0.943 | 0.464 | −1.880 | −0.032 |
| | $\beta_{21}$ | 0.484 | 0.479 | 0.758 | −1.071 | 2.052 |
| 200 | $\beta_{10}$ | 1.449 | 1.449 | 0.204 | 1.082 | 1.814 |
| | $\beta_{11}$ | 2.990 | 2.989 | 0.322 | 2.395 | 3.590 |
| | $\beta_{20}$ | −0.990 | −0.986 | 0.318 | −1.582 | −0.404 |
| | $\beta_{21}$ | 0.513 | 0.513 | 0.567 | −0.546 | 1.569 |
| 500 | $\beta_{10}$ | 1.476 | 1.476 | 0.121 | 1.239 | 1.714 |
| | $\beta_{11}$ | 2.995 | 2.994 | 0.181 | 2.631 | 3.359 |
| | $\beta_{20}$ | −0.995 | −0.993 | 0.198 | −1.380 | −0.611 |
| | $\beta_{21}$ | 0.502 | 0.502 | 0.322 | −0.139 | 1.147 |

Table 4: *Posterior* estimates for model parameters using zero-inflated artificial samples (Scenarios 3 and 4).

| $n$ | Parameter | Mean | Median | Std. Dev. | 95% HPDI | |
|---|---|---|---|---|---|---|
| | | | | | Lower | Upper |
| Scenario 3 | | | | | | |
| 50 | $\beta_{10}$ | 1.162 | 1.175 | 0.685 | −0.184 | 2.482 |
| | $\beta_{11}$ | −1.219 | −1.208 | 1.296 | −3.982 | 1.521 |
| | $\beta_{20}$ | −0.891 | −0.875 | 0.613 | −2.200 | 0.382 |
| | $\beta_{21}$ | −0.814 | −0.802 | 1.033 | −3.143 | 1.484 |
| 100 | $\beta_{10}$ | 1.380 | 1.386 | 0.476 | 0.414 | 2.335 |
| | $\beta_{11}$ | −1.536 | −1.529 | 1.021 | −3.569 | 0.479 |
| | $\beta_{20}$ | −0.944 | −0.933 | 0.468 | −1.960 | 0.057 |
| | $\beta_{21}$ | −0.896 | −0.891 | 0.819 | −2.729 | 0.928 |
| 200 | $\beta_{10}$ | 1.429 | 1.432 | 0.317 | 0.846 | 2.009 |
| | $\beta_{11}$ | −1.505 | −1.499 | 0.725 | −2.882 | −0.138 |
| | $\beta_{20}$ | −0.994 | −0.989 | 0.325 | −1.644 | −0.353 |
| | $\beta_{21}$ | −0.934 | −0.929 | 0.633 | −2.218 | 0.335 |
| 500 | $\beta_{10}$ | 1.476 | 1.477 | 0.196 | 1.108 | 1.843 |
| | $\beta_{11}$ | −1.502 | −1.500 | 0.413 | −2.317 | −0.690 |
| | $\beta_{20}$ | −0.995 | −0.993 | 0.225 | −1.418 | −0.575 |
| | $\beta_{21}$ | −0.957 | −0.956 | 0.406 | −1.736 | −0.182 |
| Scenario 4 | | | | | | |
| 50 | $\beta_{10}$ | 1.279 | 1.287 | 0.581 | 0.141 | 2.402 |
| | $\beta_{11}$ | −1.363 | −1.356 | 1.003 | −3.430 | 0.697 |
| | $\beta_{20}$ | −0.914 | −0.901 | 0.610 | −2.122 | 0.275 |
| | $\beta_{21}$ | 0.437 | 0.434 | 1.051 | −1.570 | 2.445 |
| 100 | $\beta_{10}$ | 1.386 | 1.391 | 0.424 | 0.567 | 2.198 |
| | $\beta_{11}$ | −1.438 | −1.437 | 0.762 | −2.947 | 0.066 |
| | $\beta_{20}$ | −0.932 | −0.925 | 0.462 | −1.860 | −0.016 |
| | $\beta_{21}$ | 0.424 | 0.421 | 0.834 | −1.137 | 1.991 |
| 200 | $\beta_{10}$ | 1.448 | 1.450 | 0.253 | 0.945 | 1.949 |
| | $\beta_{11}$ | −1.489 | −1.487 | 0.486 | −2.502 | −0.477 |
| | $\beta_{20}$ | −0.960 | −0.957 | 0.280 | −1.549 | −0.376 |
| | $\beta_{21}$ | 0.467 | 0.466 | 0.518 | −0.587 | 1.522 |
| 500 | $\beta_{10}$ | 1.475 | 1.476 | 0.166 | 1.150 | 1.799 |
| | $\beta_{11}$ | −1.478 | −1.478 | 0.304 | −2.083 | −0.873 |
| | $\beta_{20}$ | −1.001 | −1.000 | 0.204 | −1.388 | −0.616 |
| | $\beta_{21}$ | 0.510 | 0.509 | 0.343 | −0.133 | 1.155 |

Table 5: Coverage probabilities (%) of the HPDIs using zero-inflated artificial samples (Scenarios 1 and 2).

| $n$ | Parameter | BNCP | CP | ANCP | BNCP | CP | ANCP |
|---|---|---|---|---|---|---|---|
| | | | Scenario 1 | | | Scenario 2 | |
| 50 | $\beta_{10}$ | 3.00 | 92.80 | 4.20 | 1.00 | 93.80 | 5.20 |
| | $\beta_{11}$ | 1.00 | 88.00 | 11.00 | 1.80 | 93.40 | 4.80 |
| | $\beta_{20}$ | 1.80 | 98.20 | 0.00 | 3.60 | 95.80 | 0.60 |
| | $\beta_{21}$ | 1.60 | 98.00 | 0.40 | 1.00 | 97.60 | 1.40 |
| 100 | $\beta_{10}$ | 2.20 | 93.60 | 4.20 | 1.60 | 94.00 | 4.40 |
| | $\beta_{11}$ | 1.80 | 92.60 | 5.60 | 1.20 | 95.20 | 3.60 |
| | $\beta_{20}$ | 1.80 | 97.20 | 1.00 | 4.00 | 94.40 | 1.60 |
| | $\beta_{21}$ | 2.00 | 97.20 | 0.80 | 2.00 | 95.00 | 3.00 |
| 200 | $\beta_{10}$ | 1.60 | 93.00 | 5.40 | 2.40 | 92.60 | 5.00 |
| | $\beta_{11}$ | 1.60 | 93.20 | 5.20 | 2.20 | 94.00 | 3.80 |
| | $\beta_{20}$ | 3.20 | 95.00 | 1.80 | 3.80 | 94.00 | 2.20 |
| | $\beta_{21}$ | 3.00 | 95.60 | 1.40 | 3.20 | 93.80 | 3.00 |
| 500 | $\beta_{10}$ | 1.20 | 94.00 | 4.80 | 1.00 | 95.40 | 3.60 |
| | $\beta_{11}$ | 2.60 | 94.60 | 2.80 | 1.40 | 96.00 | 2.60 |
| | $\beta_{20}$ | 2.40 | 95.20 | 2.40 | 3.20 | 94.80 | 2.00 |
| | $\beta_{21}$ | 2.60 | 95.20 | 2.20 | 2.40 | 95.40 | 2.20 |

Table 6: Coverage probabilities (%) of the HPDIs using zero-inflated artificial samples (Scenarios 3 and 4).

| $n$ | Parameter | BNCP | CP | ANCP | BNCP | CP | ANCP |
|---|---|---|---|---|---|---|---|
| | | | Scenario 3 | | | Scenario 4 | |
| 50 | $\beta_{10}$ | 0.40 | 95.40 | 4.20 | 0.20 | 96.80 | 3.00 |
| | $\beta_{11}$ | 3.20 | 96.40 | 0.40 | 2.40 | 97.00 | 0.60 |
| | $\beta_{20}$ | 2.20 | 97.40 | 0.40 | 4.60 | 94.40 | 1.00 |
| | $\beta_{21}$ | 1.80 | 97.60 | 0.60 | 1.80 | 94.80 | 3.40 |
| 100 | $\beta_{10}$ | 1.40 | 95.80 | 2.80 | 1.20 | 95.00 | 3.80 |
| | $\beta_{11}$ | 2.20 | 96.20 | 1.60 | 2.60 | 95.00 | 2.40 |
| | $\beta_{20}$ | 2.40 | 97.40 | 0.20 | 4.20 | 94.60 | 1.20 |
| | $\beta_{21}$ | 1.80 | 97.60 | 0.60 | 1.80 | 93.60 | 4.60 |
| 200 | $\beta_{10}$ | 2.00 | 94.40 | 3.60 | 1.20 | 96.60 | 2.20 |
| | $\beta_{11}$ | 3.20 | 95.00 | 1.80 | 1.80 | 97.00 | 1.20 |
| | $\beta_{20}$ | 2.20 | 96.20 | 1.60 | 3.20 | 95.80 | 1.00 |
| | $\beta_{21}$ | 2.80 | 95.60 | 1.60 | 2.40 | 94.40 | 3.20 |
| 500 | $\beta_{10}$ | 2.20 | 94.40 | 3.40 | 2.20 | 95.80 | 2.00 |
| | $\beta_{11}$ | 2.40 | 94.80 | 2.80 | 2.40 | 95.40 | 2.20 |
| | $\beta_{20}$ | 3.80 | 94.40 | 1.80 | 2.20 | 95.00 | 2.80 |
| | $\beta_{21}$ | 2.80 | 95.20 | 2.00 | 3.40 | 94.60 | 2.00 |

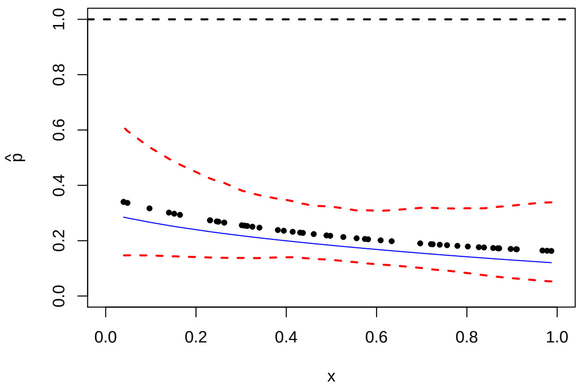


(a) $n = 50$

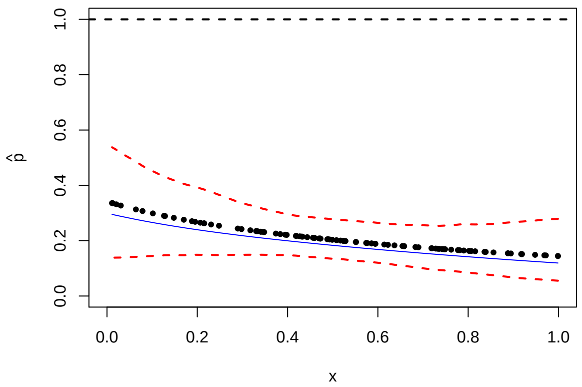


(b) $n = 100$

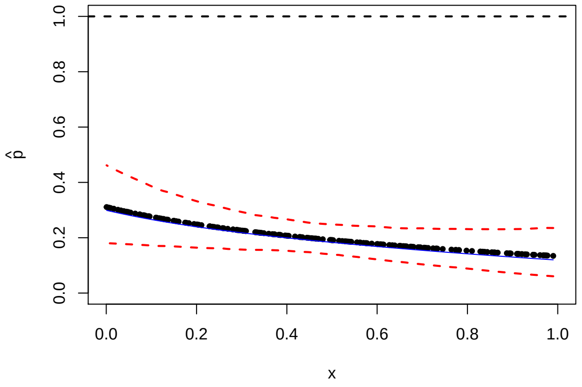


(c) $n = 200$

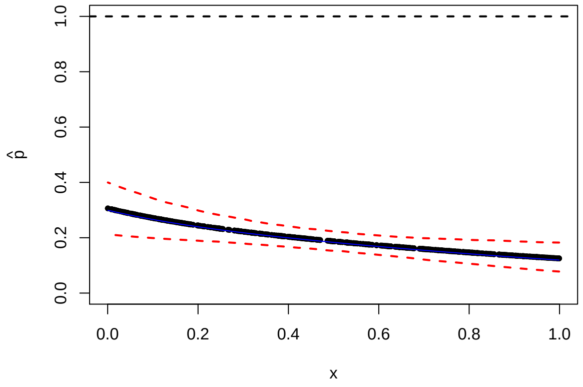


(d) $n = 500$

Figure 1: *Posterior* estimates for parameter $p$ using zero-inflated artificial samples (Scenario 1).

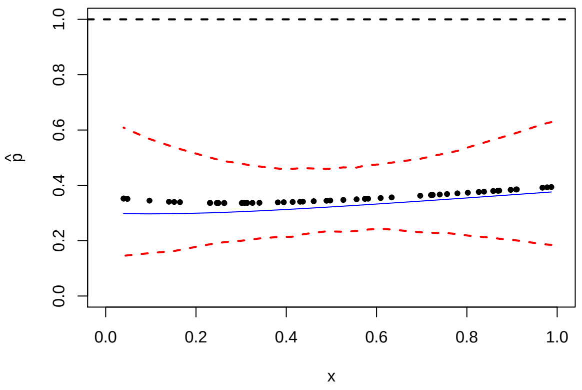


(a) $n = 50$

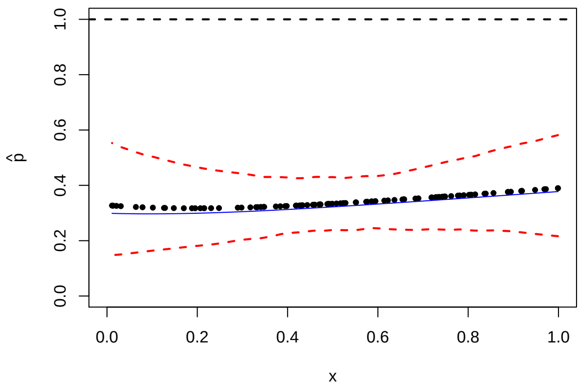


(b) $n = 100$

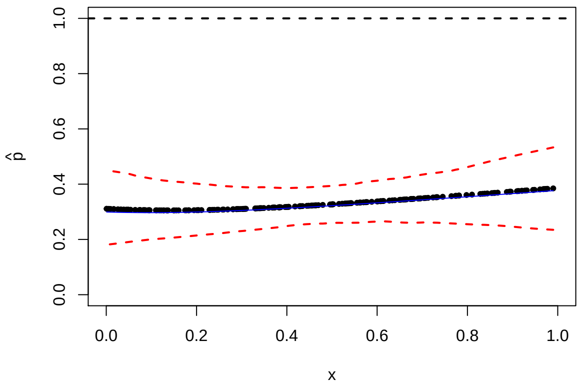


(c) $n = 200$

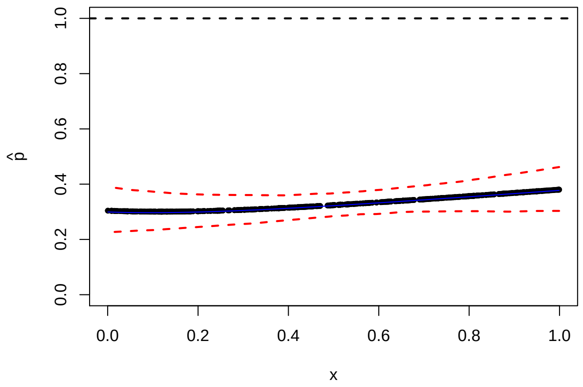


(d) $n = 500$

Figure 2: *Posterior* estimates for parameter $p$ using zero-inflated artificial samples (Scenario 2).

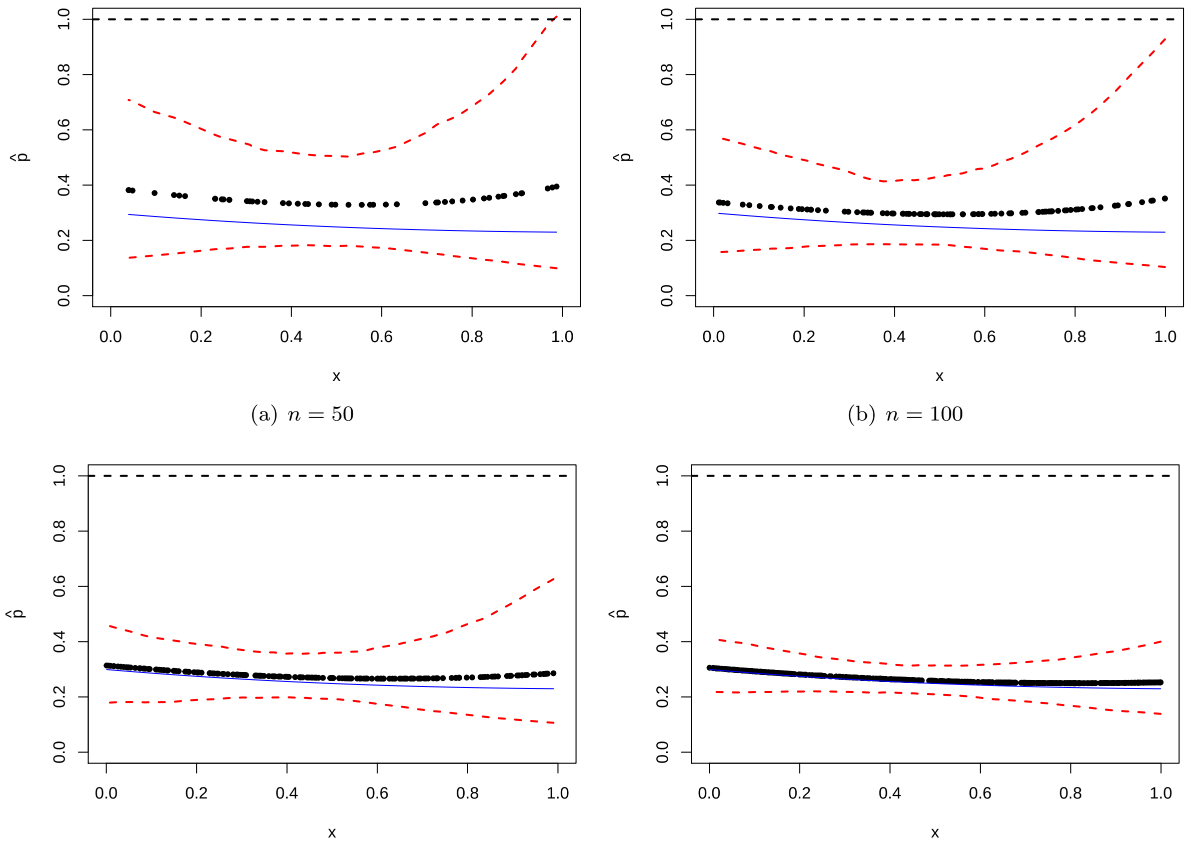


(a) $n = 50$ (b) $n = 100$

(c) $n = 200$ (d) $n = 500$

Figure 3: *Posterior* estimates for parameter $p$ using zero-inflated artificial samples (Scenario 3).

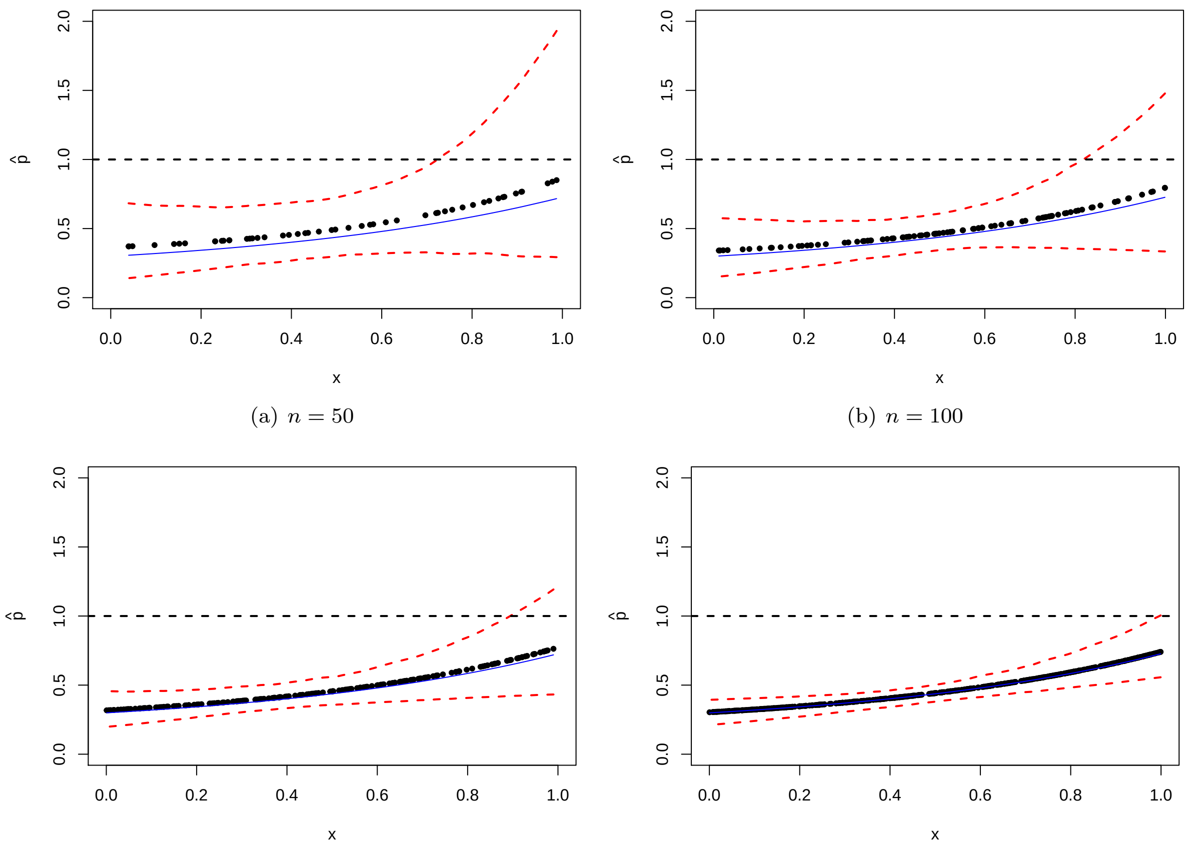


(a) $n = 50$ (b) $n = 100$

(c) $n = 200$ (d) $n = 500$

Figure 4: *Posterior* estimates for parameter $p$ using zero-inflated artificial samples (Scenario 4).

## 1.2 Using *probit* link function

Table 7: Empirical properties of the Bayesian estimators using zero-inflated artificial samples (Scenarios 1 and 2).

| $n$ | Parameter | Bias | MSE | $\sqrt{\frac{\text{MSE}}{\text{Var}}}$ | MAPE (%) |
|---|---|---|---|---|---|
| | | | Scenario 1 | | |
| 50 | $\beta_{10}$ | −0.013 | 0.992 | 1.000 | 49.716 |
| | $\beta_{11}$ | −1.969 | 9.844 | 1.284 | 81.167 |
| | $\beta_{20}$ | 0.020 | 0.214 | 1.001 | 36.875 |
| | $\beta_{21}$ | 0.191 | 0.754 | 1.025 | 69.121 |
| 100 | $\beta_{10}$ | −0.007 | 0.415 | 1.000 | 32.842 |
| | $\beta_{11}$ | −0.847 | 3.051 | 1.144 | 42.982 |
| | $\beta_{20}$ | 0.003 | 0.148 | 1.000 | 30.516 |
| | $\beta_{21}$ | 0.063 | 0.564 | 1.004 | 59.161 |
| 200 | $\beta_{10}$ | −0.010 | 0.134 | 1.000 | 18.566 |
| | $\beta_{11}$ | −0.405 | 1.003 | 1.093 | 24.666 |
| | $\beta_{20}$ | 0.009 | 0.054 | 1.001 | 18.170 |
| | $\beta_{21}$ | 0.010 | 0.266 | 1.000 | 40.134 |
| 500 | $\beta_{10}$ | −0.005 | 0.052 | 1.000 | 12.110 |
| | $\beta_{11}$ | −0.138 | 0.236 | 1.043 | 12.837 |
| | $\beta_{20}$ | 0.001 | 0.025 | 1.000 | 12.704 |
| | $\beta_{21}$ | 0.004 | 0.096 | 1.000 | 24.369 |
| | | | Scenario 2 | | |
| 50 | $\beta_{10}$ | −0.213 | 0.299 | 1.086 | 27.133 |
| | $\beta_{11}$ | −0.117 | 0.586 | 1.012 | 19.488 |
| | $\beta_{20}$ | 0.052 | 0.180 | 1.008 | 33.672 |
| | $\beta_{21}$ | −0.026 | 0.497 | 1.001 | 108.882 |
| 100 | $\beta_{10}$ | −0.131 | 0.143 | 1.066 | 19.972 |
| | $\beta_{11}$ | −0.026 | 0.292 | 1.001 | 14.063 |
| | $\beta_{20}$ | 0.003 | 0.108 | 1.000 | 26.370 |
| | $\beta_{21}$ | −0.002 | 0.311 | 1.000 | 89.775 |
| 200 | $\beta_{10}$ | −0.056 | 0.055 | 1.030 | 12.321 |
| | $\beta_{11}$ | −0.018 | 0.121 | 1.001 | 9.102 |
| | $\beta_{20}$ | −0.002 | 0.035 | 1.000 | 15.016 |
| | $\beta_{21}$ | 0.006 | 0.114 | 1.000 | 54.306 |
| 500 | $\beta_{10}$ | −0.030 | 0.024 | 1.020 | 8.184 |
| | $\beta_{11}$ | 0.004 | 0.052 | 1.000 | 6.078 |
| | $\beta_{20}$ | 0.003 | 0.016 | 1.000 | 10.023 |
| | $\beta_{21}$ | −0.004 | 0.043 | 1.000 | 32.685 |

Table 8: Empirical properties of the Bayesian estimators using zero-inflated artificial samples (Scenarios 3 and 4).

| $n$ | Parameter | Bias | MSE | $\sqrt{\frac{\text{MSE}}{\text{Var}}}$ | MAPE (%) |
|---|---|---|---|---|---|
| | | | Scenario 3 | | |
| 50 | $\beta_{10}$ | −0.558 | 1.168 | 1.167 | 54.835 |
| | $\beta_{11}$ | 0.371 | 2.334 | 1.031 | 81.328 |
| | $\beta_{20}$ | 0.064 | 0.217 | 1.009 | 37.291 |
| | $\beta_{21}$ | 0.090 | 0.739 | 1.006 | 69.044 |
| 100 | $\beta_{10}$ | −0.335 | 0.687 | 1.093 | 41.996 |
| | $\beta_{11}$ | 0.157 | 2.126 | 1.006 | 79.353 |
| | $\beta_{20}$ | 0.000 | 0.142 | 1.000 | 29.447 |
| | $\beta_{21}$ | 0.085 | 0.498 | 1.007 | 54.999 |
| 200 | $\beta_{10}$ | −0.131 | 0.215 | 1.042 | 23.877 |
| | $\beta_{11}$ | −0.013 | 1.186 | 1.000 | 57.991 |
| | $\beta_{20}$ | 0.008 | 0.057 | 1.001 | 18.644 |
| | $\beta_{21}$ | 0.024 | 0.260 | 1.001 | 39.806 |
| 500 | $\beta_{10}$ | −0.043 | 0.078 | 1.012 | 14.353 |
| | $\beta_{11}$ | −0.058 | 0.534 | 1.003 | 37.506 |
| | $\beta_{20}$ | 0.007 | 0.022 | 1.001 | 11.511 |
| | $\beta_{21}$ | −0.001 | 0.092 | 1.000 | 23.509 |
| | | | Scenario 4 | | |
| 50 | $\beta_{10}$ | −0.300 | 0.621 | 1.081 | 39.539 |
| | $\beta_{11}$ | 0.201 | 1.467 | 1.014 | 64.091 |
| | $\beta_{20}$ | 0.101 | 0.173 | 1.031 | 33.391 |
| | $\beta_{21}$ | −0.119 | 0.464 | 1.016 | 107.901 |
| 100 | $\beta_{10}$ | −0.116 | 0.306 | 1.023 | 28.224 |
| | $\beta_{11}$ | 0.066 | 0.849 | 1.003 | 47.504 |
| | $\beta_{20}$ | −0.017 | 0.099 | 1.002 | 24.656 |
| | $\beta_{21}$ | 0.045 | 0.269 | 1.004 | 82.445 |
| 200 | $\beta_{10}$ | −0.089 | 0.115 | 1.036 | 17.815 |
| | $\beta_{11}$ | 0.058 | 0.372 | 1.005 | 32.020 |
| | $\beta_{20}$ | 0.001 | 0.042 | 1.000 | 16.520 |
| | $\beta_{21}$ | 0.016 | 0.132 | 1.001 | 57.849 |
| 500 | $\beta_{10}$ | −0.026 | 0.041 | 1.008 | 10.693 |
| | $\beta_{11}$ | 0.001 | 0.136 | 1.000 | 19.688 |
| | $\beta_{20}$ | 0.000 | 0.015 | 1.000 | 9.807 |
| | $\beta_{21}$ | 0.014 | 0.042 | 1.002 | 33.297 |

Table 9: *Posterior* estimates for model parameters using zero-inflated artificial samples (Scenarios 1 and 2).

| $n$ | Parameter | Mean | Median | Std. Dev. | 95% HPDI | |
|---|---|---|---|---|---|---|
| | | | | | Lower | Upper |
| Scenario 1 | | | | | | |
| 50 | $\beta_{10}$ | 1.487 | 1.490 | 0.996 | −0.136 | 3.104 |
| | $\beta_{11}$ | 1.031 | 1.022 | 2.442 | −2.290 | 4.386 |
| | $\beta_{20}$ | −0.980 | −0.964 | 0.462 | −1.971 | −0.013 |
| | $\beta_{21}$ | −0.809 | −0.784 | 0.847 | −2.737 | 1.072 |
| 100 | $\beta_{10}$ | 1.493 | 1.495 | 0.644 | 0.330 | 2.654 |
| | $\beta_{11}$ | 2.153 | 2.141 | 1.527 | −0.182 | 4.495 |
| | $\beta_{20}$ | −0.997 | −0.988 | 0.384 | −1.754 | −0.256 |
| | $\beta_{21}$ | −0.937 | −0.926 | 0.748 | −2.419 | 0.521 |
| 200 | $\beta_{10}$ | 1.490 | 1.491 | 0.367 | 0.818 | 2.158 |
| | $\beta_{11}$ | 2.595 | 2.585 | 0.916 | 1.128 | 4.080 |
| | $\beta_{20}$ | −0.991 | −0.988 | 0.233 | −1.458 | −0.528 |
| | $\beta_{21}$ | −0.990 | −0.980 | 0.516 | −2.004 | 0.015 |
| 500 | $\beta_{10}$ | 1.495 | 1.495 | 0.228 | 1.078 | 1.911 |
| | $\beta_{11}$ | 2.862 | 2.857 | 0.465 | 2.022 | 3.707 |
| | $\beta_{20}$ | −0.999 | −0.998 | 0.158 | −1.300 | −0.701 |
| | $\beta_{21}$ | −0.996 | −0.993 | 0.310 | −1.602 | −0.392 |
| Scenario 2 | | | | | | |
| 50 | $\beta_{10}$ | 1.287 | 1.286 | 0.504 | 0.339 | 2.232 |
| | $\beta_{11}$ | 2.883 | 2.876 | 0.757 | 1.478 | 4.296 |
| | $\beta_{20}$ | −0.948 | −0.939 | 0.421 | −1.767 | −0.139 |
| | $\beta_{21}$ | 0.474 | 0.470 | 0.704 | −0.863 | 1.819 |
| 100 | $\beta_{10}$ | 1.369 | 1.369 | 0.355 | 0.656 | 2.079 |
| | $\beta_{11}$ | 2.974 | 2.970 | 0.540 | 1.887 | 4.067 |
| | $\beta_{20}$ | −0.997 | −0.992 | 0.328 | −1.623 | −0.380 |
| | $\beta_{21}$ | 0.498 | 0.495 | 0.558 | −0.536 | 1.537 |
| 200 | $\beta_{10}$ | 1.444 | 1.443 | 0.227 | 0.991 | 1.897 |
| | $\beta_{11}$ | 2.982 | 2.979 | 0.347 | 2.265 | 3.701 |
| | $\beta_{20}$ | −1.002 | −1.000 | 0.188 | −1.395 | −0.612 |
| | $\beta_{21}$ | 0.506 | 0.505 | 0.338 | −0.186 | 1.199 |
| 500 | $\beta_{10}$ | 1.470 | 1.470 | 0.152 | 1.176 | 1.763 |
| | $\beta_{11}$ | 3.004 | 3.003 | 0.228 | 2.568 | 3.443 |
| | $\beta_{20}$ | −0.997 | −0.996 | 0.124 | −1.252 | −0.742 |
| | $\beta_{21}$ | 0.496 | 0.496 | 0.207 | 0.077 | 0.917 |

Table 10: *Posterior* estimates for model parameters using zero-inflated artificial samples (Scenarios 3 and 4).

| $n$ | Parameter | Mean | Median | Std. Dev. | 95% HPDI | |
|---|---|---|---|---|---|---|
| | | | | | Lower | Upper |
| Scenario 3 | | | | | | |
| 50 | $\beta_{10}$ | 0.942 | 0.961 | 0.926 | −1.084 | 2.925 |
| | $\beta_{11}$ | −1.129 | −1.127 | 1.482 | −5.479 | 3.199 |
| | $\beta_{20}$ | −0.936 | −0.922 | 0.462 | −1.922 | 0.025 |
| | $\beta_{21}$ | −0.910 | −0.880 | 0.855 | −2.863 | 0.986 |
| 100 | $\beta_{10}$ | 1.165 | 1.180 | 0.758 | −0.395 | 2.695 |
| | $\beta_{11}$ | −1.343 | −1.338 | 1.450 | −4.798 | 2.099 |
| | $\beta_{20}$ | −1.000 | −0.991 | 0.377 | −1.757 | −0.260 |
| | $\beta_{21}$ | −0.915 | −0.904 | 0.701 | −2.390 | 0.539 |
| 200 | $\beta_{10}$ | 1.369 | 1.375 | 0.445 | 0.502 | 2.224 |
| | $\beta_{11}$ | −1.513 | −1.501 | 1.089 | −3.844 | 0.809 |
| | $\beta_{20}$ | −0.992 | −0.989 | 0.238 | −1.458 | −0.532 |
| | $\beta_{21}$ | −0.976 | −0.966 | 0.510 | −1.984 | 0.019 |
| 500 | $\beta_{10}$ | 1.457 | 1.459 | 0.275 | 0.921 | 1.986 |
| | $\beta_{11}$ | −1.558 | −1.551 | 0.728 | −2.976 | −0.152 |
| | $\beta_{20}$ | −0.993 | −0.991 | 0.149 | −1.293 | −0.694 |
| | $\beta_{21}$ | −1.001 | −0.998 | 0.304 | −1.605 | −0.399 |
| Scenario 4 | | | | | | |
| 50 | $\beta_{10}$ | 1.200 | 1.211 | 0.729 | −0.199 | 2.576 |
| | $\beta_{11}$ | −1.299 | −1.292 | 1.194 | −3.770 | 1.162 |
| | $\beta_{20}$ | −0.899 | −0.891 | 0.404 | −1.710 | −0.099 |
| | $\beta_{21}$ | 0.381 | 0.379 | 0.670 | −0.951 | 1.720 |
| 100 | $\beta_{10}$ | 1.384 | 1.390 | 0.541 | 0.325 | 2.433 |
| | $\beta_{11}$ | −1.434 | −1.431 | 0.919 | −3.288 | 0.421 |
| | $\beta_{20}$ | −1.017 | −1.013 | 0.314 | −1.644 | −0.396 |
| | $\beta_{21}$ | 0.545 | 0.542 | 0.517 | −0.490 | 1.587 |
| 200 | $\beta_{10}$ | 1.411 | 1.414 | 0.327 | 0.772 | 2.041 |
| | $\beta_{11}$ | −1.442 | −1.440 | 0.607 | −2.652 | −0.232 |
| | $\beta_{20}$ | −0.999 | −0.997 | 0.205 | −1.392 | −0.610 |
| | $\beta_{21}$ | 0.516 | 0.516 | 0.363 | −0.175 | 1.208 |
| 500 | $\beta_{10}$ | 1.474 | 1.476 | 0.200 | 1.069 | 1.878 |
| | $\beta_{11}$ | −1.499 | −1.498 | 0.369 | −2.223 | −0.776 |
| | $\beta_{20}$ | −1.000 | −0.999 | 0.122 | −1.254 | −0.745 |
| | $\beta_{21}$ | 0.514 | 0.513 | 0.204 | 0.095 | 0.933 |

Table 11: Coverage probabilities (%) of the HPDIs using zero-inflated artificial samples (Scenarios 1 and 2).

| $n$ | Parameter | BNCP | CP | ANCP | BNCP | CP | ANCP |
|---|---|---|---|---|---|---|---|
| | | | Scenario 1 | | | Scenario 2 | |
| 50 | $\beta_{10}$ | 2.60 | 94.60 | 2.80 | 1.20 | 93.20 | 5.60 |
| | $\beta_{11}$ | 0.80 | 73.80 | 25.40 | 1.60 | 93.60 | 4.80 |
| | $\beta_{20}$ | 2.40 | 97.40 | 0.20 | 5.20 | 93.80 | 1.00 |
| | $\beta_{21}$ | 2.40 | 97.60 | 0.00 | 1.60 | 95.20 | 3.20 |
| 100 | $\beta_{10}$ | 2.00 | 96.20 | 1.80 | 1.20 | 95.40 | 3.40 |
| | $\beta_{11}$ | 0.40 | 87.00 | 12.60 | 1.20 | 95.20 | 3.60 |
| | $\beta_{20}$ | 4.20 | 94.60 | 1.20 | 3.40 | 95.00 | 1.60 |
| | $\beta_{21}$ | 3.00 | 95.80 | 1.20 | 3.00 | 93.80 | 3.20 |
| 200 | $\beta_{10}$ | 4.20 | 92.40 | 3.40 | 1.00 | 95.20 | 3.80 |
| | $\beta_{11}$ | 0.60 | 90.00 | 9.40 | 1.60 | 95.60 | 2.80 |
| | $\beta_{20}$ | 3.20 | 94.80 | 2.00 | 2.00 | 96.40 | 1.60 |
| | $\beta_{21}$ | 3.40 | 95.40 | 1.20 | 1.80 | 96.60 | 1.60 |
| 500 | $\beta_{10}$ | 3.40 | 95.20 | 1.40 | 2.80 | 93.60 | 3.60 |
| | $\beta_{11}$ | 1.00 | 92.60 | 6.40 | 1.60 | 95.00 | 3.40 |
| | $\beta_{20}$ | 2.80 | 95.20 | 2.00 | 2.20 | 96.40 | 1.40 |
| | $\beta_{21}$ | 2.60 | 95.40 | 2.00 | 2.20 | 95.60 | 2.20 |

Table 12: Coverage probabilities (%) of the HPDIs using zero-inflated artificial samples (Scenarios 3 and 4).

| $n$ | Parameter | BNCP | CP | ANCP | BNCP | CP | ANCP |
|---|---|---|---|---|---|---|---|
| | | | Scenario 3 | | | Scenario 4 | |
| 50 | $\beta_{10}$ | 0.00 | 98.60 | 1.40 | 1.00 | 96.40 | 2.60 |
| | $\beta_{11}$ | 0.60 | 99.40 | 0.00 | 2.80 | 96.40 | 0.80 |
| | $\beta_{20}$ | 3.20 | 96.80 | 0.00 | 4.40 | 94.80 | 0.80 |
| | $\beta_{21}$ | 2.40 | 97.00 | 0.60 | 1.20 | 94.40 | 4.40 |
| 100 | $\beta_{10}$ | 0.40 | 98.00 | 1.60 | 2.60 | 96.00 | 1.40 |
| | $\beta_{11}$ | 1.00 | 98.60 | 0.40 | 1.00 | 97.40 | 1.60 |
| | $\beta_{20}$ | 2.60 | 96.60 | 0.80 | 1.60 | 97.20 | 1.20 |
| | $\beta_{21}$ | 3.80 | 95.40 | 0.80 | 2.00 | 96.60 | 1.40 |
| 200 | $\beta_{10}$ | 0.40 | 96.60 | 3.00 | 1.80 | 95.60 | 2.60 |
| | $\beta_{11}$ | 1.20 | 98.00 | 0.80 | 2.20 | 96.20 | 1.60 |
| | $\beta_{20}$ | 3.00 | 95.00 | 2.00 | 2.60 | 96.20 | 1.20 |
| | $\beta_{21}$ | 3.80 | 95.80 | 0.40 | 1.60 | 95.40 | 3.00 |
| 500 | $\beta_{10}$ | 1.80 | 96.20 | 2.00 | 2.20 | 96.00 | 1.80 |
| | $\beta_{11}$ | 2.00 | 95.60 | 2.40 | 2.00 | 95.60 | 2.40 |
| | $\beta_{20}$ | 3.20 | 94.80 | 2.00 | 2.80 | 95.60 | 1.60 |
| | $\beta_{21}$ | 1.80 | 96.40 | 1.80 | 2.00 | 96.20 | 1.80 |

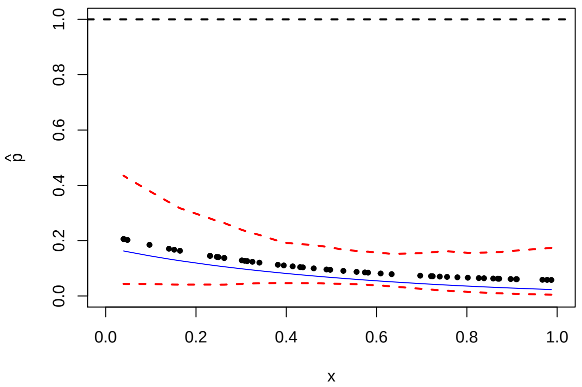


(a) $n = 50$

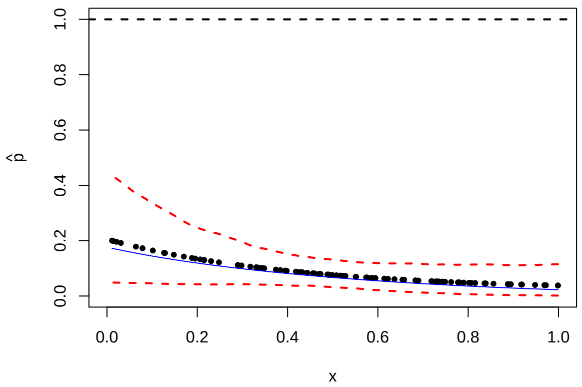


(b) $n = 100$

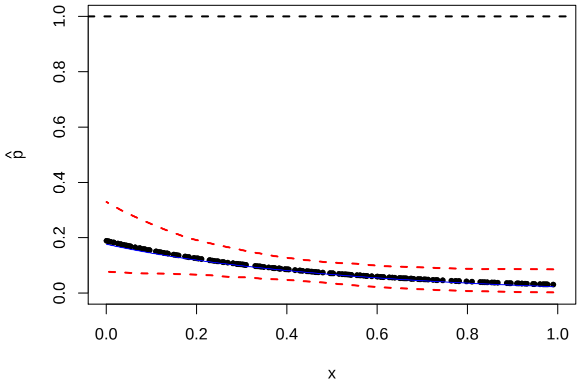


(c) $n = 200$

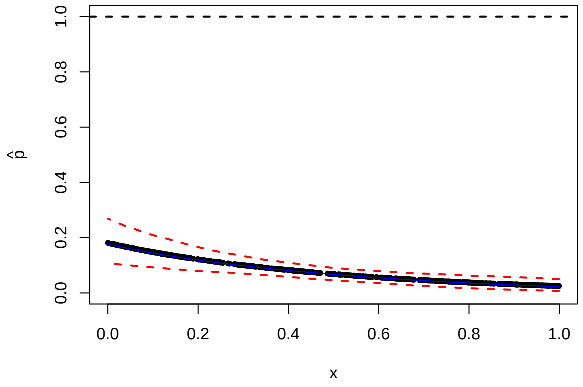


(d) $n = 500$

Figure 5: *Posterior* estimates for parameter $p$ using zero-inflated artificial samples (Scenario 1).

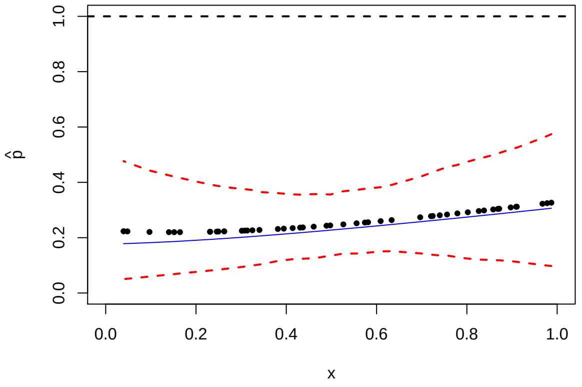


(a) $n = 50$

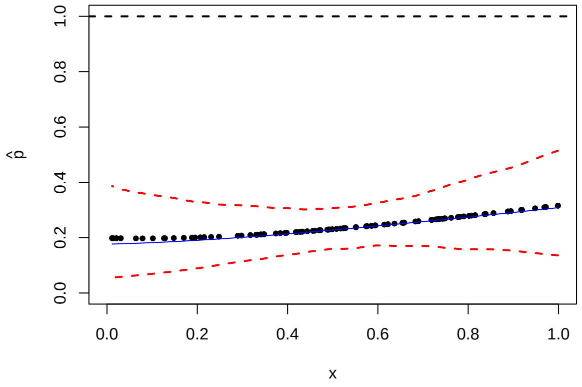


(b) $n = 100$

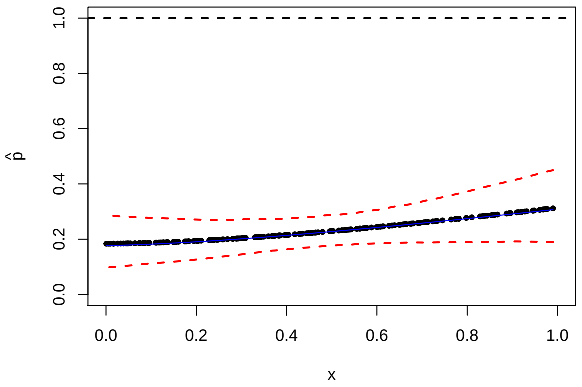


(c) $n = 200$

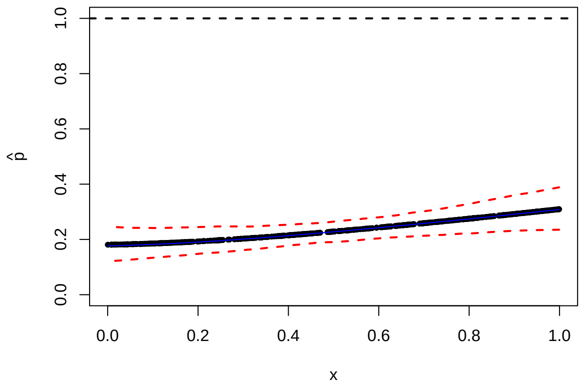


(d) $n = 500$

Figure 6: *Posterior* estimates for parameter $p$ using zero-inflated artificial samples (Scenario 2).

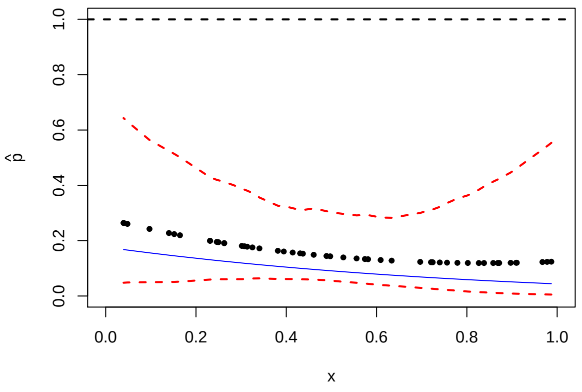


(a) $n = 50$

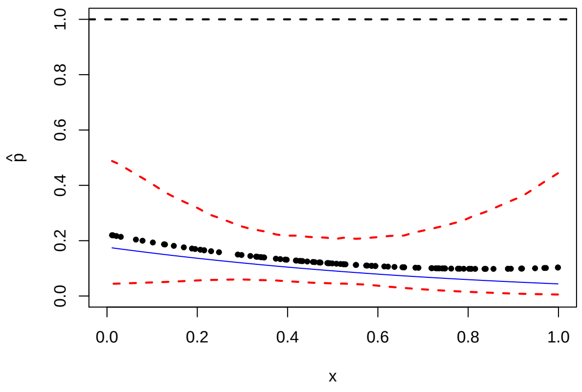


(b) $n = 100$

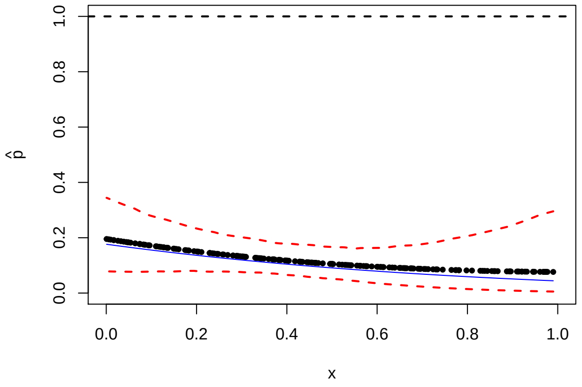


(c) $n = 200$

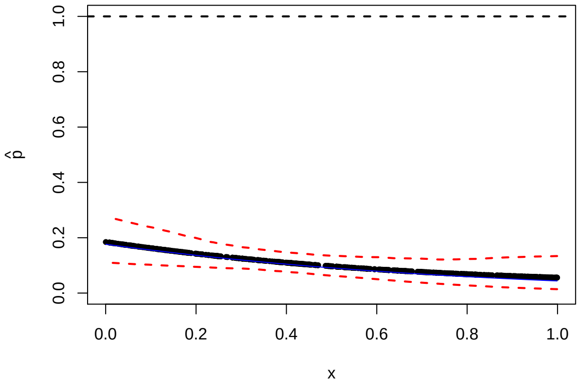


(d) $n = 500$

Figure 7: *Posterior* estimates for parameter $p$ using zero-inflated artificial samples (Scenario 3).

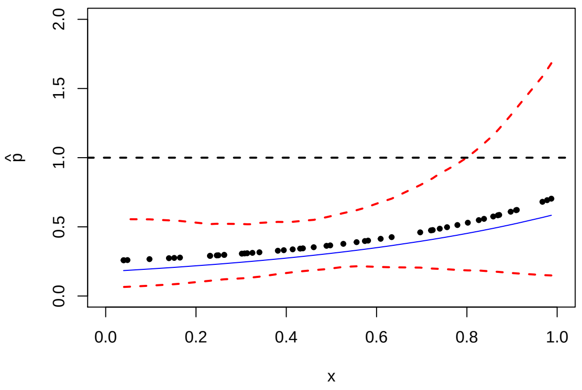


(a) $n = 50$

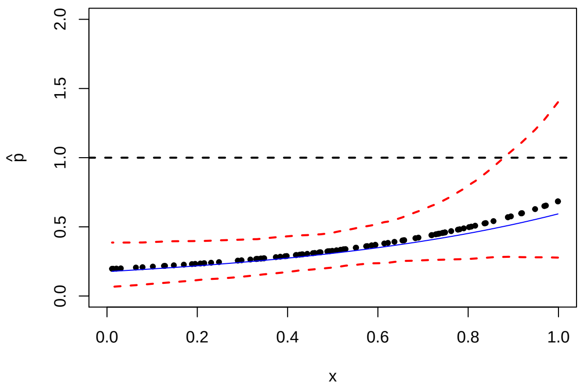


(b) $n = 100$

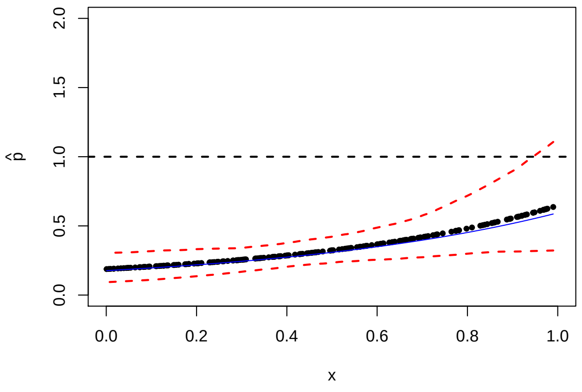


(c) $n = 200$

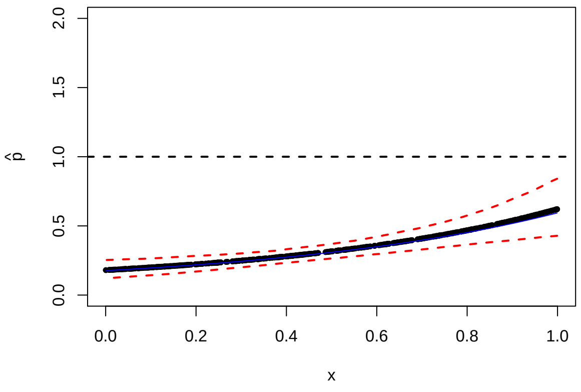


(d) $n = 500$

Figure 8: *Posterior* estimates for parameter $p$ using zero-inflated artificial samples (Scenario 4).

## 1.3 Using *complementary log-log* link function

Table 13: Empirical properties of the Bayesian estimators using zero-inflated artificial samples (Scenarios 1 and 2).

| $n$ | Parameter | Bias | MSE | $\sqrt{\frac{\text{MSE}}{\text{Var}}}$ | MAPE (%) |
|---|---|---|---|---|---|
| | | | Scenario 1 | | |
| 50 | $\beta_{10}$ | −0.145 | 0.290 | 1.038 | 27.436 |
| | $\beta_{11}$ | −0.369 | 1.254 | 1.059 | 26.086 |
| | $\beta_{20}$ | 0.028 | 0.260 | 1.002 | 40.885 |
| | $\beta_{21}$ | 0.222 | 0.816 | 1.032 | 72.841 |
| 100 | $\beta_{10}$ | −0.090 | 0.132 | 1.032 | 19.110 |
| | $\beta_{11}$ | −0.148 | 0.415 | 1.028 | 16.902 |
| | $\beta_{20}$ | 0.015 | 0.183 | 1.001 | 35.014 |
| | $\beta_{21}$ | 0.122 | 0.604 | 1.012 | 61.264 |
| 200 | $\beta_{10}$ | −0.022 | 0.047 | 1.005 | 11.434 |
| | $\beta_{11}$ | −0.107 | 0.185 | 1.032 | 11.402 |
| | $\beta_{20}$ | 0.018 | 0.074 | 1.002 | 21.593 |
| | $\beta_{21}$ | 0.060 | 0.296 | 1.006 | 42.673 |
| 500 | $\beta_{10}$ | −0.011 | 0.018 | 1.003 | 7.083 |
| | $\beta_{11}$ | −0.038 | 0.061 | 1.012 | 6.449 |
| | $\beta_{20}$ | 0.005 | 0.031 | 1.000 | 13.982 |
| | $\beta_{21}$ | 0.025 | 0.116 | 1.003 | 26.966 |
| | | | Scenario 2 | | |
| 50 | $\beta_{10}$ | −0.116 | 0.164 | 1.044 | 20.395 |
| | $\beta_{11}$ | −0.179 | 0.375 | 1.046 | 15.972 |
| | $\beta_{20}$ | 0.098 | 0.243 | 1.020 | 39.207 |
| | $\beta_{21}$ | −0.083 | 0.657 | 1.005 | 127.997 |
| 100 | $\beta_{10}$ | −0.060 | 0.074 | 1.026 | 14.508 |
| | $\beta_{11}$ | −0.106 | 0.192 | 1.030 | 11.570 |
| | $\beta_{20}$ | 0.039 | 0.129 | 1.006 | 28.591 |
| | $\beta_{21}$ | −0.018 | 0.357 | 1.000 | 96.053 |
| 200 | $\beta_{10}$ | −0.052 | 0.034 | 1.042 | 9.499 |
| | $\beta_{11}$ | −0.015 | 0.082 | 1.001 | 7.587 |
| | $\beta_{20}$ | 0.022 | 0.054 | 1.004 | 18.028 |
| | $\beta_{21}$ | −0.021 | 0.154 | 1.001 | 61.469 |
| 500 | $\beta_{10}$ | −0.017 | 0.012 | 1.012 | 5.755 |
| | $\beta_{11}$ | −0.004 | 0.028 | 1.000 | 4.473 |
| | $\beta_{20}$ | 0.006 | 0.023 | 1.001 | 12.253 |
| | $\beta_{21}$ | 0.000 | 0.062 | 1.000 | 40.099 |

Table 14: Empirical properties of the Bayesian estimators using zero-inflated artificial samples (Scenarios 3 and 4).

| $n$ | Parameter | Bias | MSE | $\sqrt{\frac{\text{MSE}}{\text{Var}}}$ | MAPE (%) |
|---|---|---|---|---|---|
| | | | Scenario 3 | | |
| 50 | $\beta_{10}$ | −0.286 | 0.547 | 1.085 | 36.060 |
| | $\beta_{11}$ | 0.242 | 1.647 | 1.018 | 67.210 |
| | $\beta_{20}$ | 0.026 | 0.281 | 1.001 | 41.631 |
| | $\beta_{21}$ | 0.248 | 0.961 | 1.034 | 78.137 |
| 100 | $\beta_{10}$ | −0.108 | 0.245 | 1.025 | 24.112 |
| | $\beta_{11}$ | −0.014 | 0.921 | 1.000 | 49.679 |
| | $\beta_{20}$ | 0.010 | 0.174 | 1.000 | 33.560 |
| | $\beta_{21}$ | 0.120 | 0.569 | 1.013 | 60.454 |
| 200 | $\beta_{10}$ | −0.055 | 0.083 | 1.019 | 14.855 |
| | $\beta_{11}$ | −0.023 | 0.442 | 1.001 | 34.941 |
| | $\beta_{20}$ | 0.012 | 0.072 | 1.001 | 21.081 |
| | $\beta_{21}$ | 0.036 | 0.278 | 1.002 | 41.432 |
| 500 | $\beta_{10}$ | −0.024 | 0.033 | 1.008 | 9.631 |
| | $\beta_{11}$ | −0.024 | 0.166 | 1.002 | 21.483 |
| | $\beta_{20}$ | 0.002 | 0.032 | 1.000 | 14.379 |
| | $\beta_{21}$ | 0.014 | 0.110 | 1.001 | 26.533 |
| | | | Scenario 4 | | |
| 50 | $\beta_{10}$ | −0.232 | 0.352 | 1.087 | 29.729 |
| | $\beta_{11}$ | 0.165 | 0.972 | 1.014 | 52.225 |
| | $\beta_{20}$ | 0.048 | 0.209 | 1.006 | 37.053 |
| | $\beta_{21}$ | −0.007 | 0.550 | 1.000 | 117.533 |
| 100 | $\beta_{10}$ | −0.116 | 0.147 | 1.049 | 20.012 |
| | $\beta_{11}$ | 0.078 | 0.434 | 1.007 | 34.804 |
| | $\beta_{20}$ | 0.037 | 0.125 | 1.006 | 28.175 |
| | $\beta_{21}$ | −0.017 | 0.331 | 1.000 | 92.817 |
| 200 | $\beta_{10}$ | −0.043 | 0.066 | 1.014 | 13.459 |
| | $\beta_{11}$ | 0.017 | 0.265 | 1.000 | 26.876 |
| | $\beta_{20}$ | 0.029 | 0.054 | 1.008 | 18.318 |
| | $\beta_{21}$ | −0.031 | 0.174 | 1.003 | 65.728 |
| 500 | $\beta_{10}$ | −0.006 | 0.022 | 1.001 | 7.707 |
| | $\beta_{11}$ | −0.015 | 0.078 | 1.001 | 14.695 |
| | $\beta_{20}$ | 0.005 | 0.022 | 1.001 | 11.691 |
| | $\beta_{21}$ | −0.012 | 0.059 | 1.001 | 38.117 |

Table 15: *Posterior* estimates for model parameters using zero-inflated artificial samples (Scenarios 1 and 2).

| $n$ | Parameter | Mean | Median | Std. Dev. | 95% HPDI Lower | 95% HPDI Upper |
|---|---|---|---|---|---|---|
| Scenario 1 | | | | | | |
| 50 | $\beta_{10}$ | 1.355 | 1.355 | 0.519 | 0.438 | 2.270 |
| | $\beta_{11}$ | 2.631 | 2.620 | 1.057 | 1.022 | 4.252 |
| | $\beta_{20}$ | −0.972 | −0.945 | 0.510 | −2.072 | 0.088 |
| | $\beta_{21}$ | −0.778 | −0.768 | 0.876 | −2.783 | 1.212 |
| 100 | $\beta_{10}$ | 1.410 | 1.411 | 0.352 | 0.740 | 2.079 |
| | $\beta_{11}$ | 2.852 | 2.844 | 0.627 | 1.679 | 4.037 |
| | $\beta_{20}$ | −0.985 | −0.969 | 0.427 | −1.837 | −0.159 |
| | $\beta_{21}$ | −0.878 | −0.877 | 0.767 | −2.449 | 0.681 |
| 200 | $\beta_{10}$ | 1.478 | 1.478 | 0.216 | 1.073 | 1.886 |
| | $\beta_{11}$ | 2.893 | 2.889 | 0.417 | 2.131 | 3.662 |
| | $\beta_{20}$ | −0.982 | −0.975 | 0.271 | −1.518 | −0.454 |
| | $\beta_{21}$ | −0.940 | −0.935 | 0.541 | −2.034 | 0.147 |
| 500 | $\beta_{10}$ | 1.489 | 1.489 | 0.135 | 1.226 | 1.752 |
| | $\beta_{11}$ | 2.962 | 2.961 | 0.243 | 2.500 | 3.423 |
| | $\beta_{20}$ | −0.995 | −0.992 | 0.176 | −1.348 | −0.648 |
| | $\beta_{21}$ | −0.975 | −0.974 | 0.339 | −1.645 | −0.307 |
| Scenario 2 | | | | | | |
| 50 | $\beta_{10}$ | 1.384 | 1.384 | 0.388 | 0.688 | 2.080 |
| | $\beta_{11}$ | 2.821 | 2.817 | 0.586 | 1.763 | 3.881 |
| | $\beta_{20}$ | −0.902 | −0.882 | 0.484 | −1.844 | 0.004 |
| | $\beta_{21}$ | 0.417 | 0.414 | 0.806 | −1.104 | 1.944 |
| 100 | $\beta_{10}$ | 1.440 | 1.440 | 0.265 | 0.917 | 1.964 |
| | $\beta_{11}$ | 2.894 | 2.892 | 0.425 | 2.079 | 3.714 |
| | $\beta_{20}$ | −0.961 | −0.950 | 0.357 | −1.685 | −0.253 |
| | $\beta_{21}$ | 0.482 | 0.478 | 0.597 | −0.709 | 1.674 |
| 200 | $\beta_{10}$ | 1.448 | 1.448 | 0.176 | 1.109 | 1.786 |
| | $\beta_{11}$ | 2.985 | 2.983 | 0.286 | 2.434 | 3.537 |
| | $\beta_{20}$ | −0.978 | −0.973 | 0.232 | −1.441 | −0.523 |
| | $\beta_{21}$ | 0.479 | 0.480 | 0.392 | −0.329 | 1.286 |
| 500 | $\beta_{10}$ | 1.483 | 1.483 | 0.108 | 1.263 | 1.704 |
| | $\beta_{11}$ | 2.996 | 2.995 | 0.167 | 2.660 | 3.333 |
| | $\beta_{20}$ | −0.994 | −0.991 | 0.152 | −1.297 | −0.694 |
| | $\beta_{21}$ | 0.500 | 0.499 | 0.248 | 0.009 | 0.994 |

Table 16: *Posterior* estimates for model parameters using zero-inflated artificial samples (Scenarios 3 and 4).

| $n$ | Parameter | Mean | Median | Std. Dev. | 95% HPDI | |
|---|---|---|---|---|---|---|
| | | | | | Lower | Upper |
| | | | Scenario 3 | | | |
| 50 | $\beta_{10}$ | 1.214 | 1.223 | 0.682 | −0.069 | 2.474 |
| | $\beta_{11}$ | −1.258 | −1.248 | 1.260 | −3.918 | 1.391 |
| | $\beta_{20}$ | −0.974 | −0.949 | 0.530 | −2.073 | 0.081 |
| | $\beta_{21}$ | −0.752 | −0.743 | 0.949 | −2.749 | 1.224 |
| 100 | $\beta_{10}$ | 1.392 | 1.398 | 0.483 | 0.477 | 2.293 |
| | $\beta_{11}$ | −1.514 | −1.510 | 0.960 | −3.446 | 0.395 |
| | $\beta_{20}$ | −0.990 | −0.974 | 0.418 | −1.845 | −0.162 |
| | $\beta_{21}$ | −0.880 | −0.878 | 0.745 | −2.450 | 0.691 |
| 200 | $\beta_{10}$ | 1.445 | 1.447 | 0.283 | 0.903 | 1.986 |
| | $\beta_{11}$ | −1.523 | −1.518 | 0.664 | −2.827 | −0.225 |
| | $\beta_{20}$ | −0.988 | −0.981 | 0.268 | −1.530 | −0.457 |
| | $\beta_{21}$ | −0.964 | −0.959 | 0.526 | −2.069 | 0.134 |
| 500 | $\beta_{10}$ | 1.476 | 1.478 | 0.180 | 1.129 | 1.821 |
| | $\beta_{11}$ | −1.524 | −1.522 | 0.407 | −2.307 | −0.745 |
| | $\beta_{20}$ | −0.998 | −0.995 | 0.180 | −1.350 | −0.648 |
| | $\beta_{21}$ | −0.986 | −0.985 | 0.331 | −1.659 | −0.315 |
| | | | Scenario 4 | | | |
| 50 | $\beta_{10}$ | 1.268 | 1.275 | 0.546 | 0.231 | 2.295 |
| | $\beta_{11}$ | −1.335 | −1.330 | 0.972 | −3.202 | 0.524 |
| | $\beta_{20}$ | −0.952 | −0.933 | 0.455 | −1.901 | −0.037 |
| | $\beta_{21}$ | 0.493 | 0.490 | 0.742 | −1.036 | 2.025 |
| 100 | $\beta_{10}$ | 1.384 | 1.389 | 0.365 | 0.624 | 2.141 |
| | $\beta_{11}$ | −1.422 | −1.421 | 0.654 | −2.805 | −0.033 |
| | $\beta_{20}$ | −0.963 | −0.951 | 0.352 | −1.690 | −0.256 |
| | $\beta_{21}$ | 0.483 | 0.480 | 0.575 | −0.706 | 1.677 |
| 200 | $\beta_{10}$ | 1.457 | 1.458 | 0.254 | 0.990 | 1.921 |
| | $\beta_{11}$ | −1.483 | −1.482 | 0.515 | −2.415 | −0.551 |
| | $\beta_{20}$ | −0.971 | −0.967 | 0.231 | −1.433 | −0.517 |
| | $\beta_{21}$ | 0.469 | 0.469 | 0.416 | −0.337 | 1.275 |
| 500 | $\beta_{10}$ | 1.494 | 1.494 | 0.146 | 1.193 | 1.792 |
| | $\beta_{11}$ | −1.515 | −1.514 | 0.278 | −2.074 | −0.957 |
| | $\beta_{20}$ | −0.995 | −0.993 | 0.146 | −1.299 | −0.694 |
| | $\beta_{21}$ | 0.488 | 0.488 | 0.242 | −0.004 | 0.983 |

Table 17: Coverage probabilities (%) of the HPDIs using zero-inflated artificial samples (Scenarios 1 and 2).

| $n$ | Parameter | BNCP | CP | ANCP | BNCP | CP | ANCP |
|---|---|---|---|---|---|---|---|
| | | | Scenario 1 | | | Scenario 2 | |
| 50 | $\beta_{10}$ | 1.60 | 92.40 | 6.00 | 2.00 | 91.80 | 6.20 |
| | $\beta_{11}$ | 2.00 | 89.40 | 8.60 | 1.80 | 91.20 | 7.00 |
| | $\beta_{20}$ | 4.60 | 95.20 | 0.20 | 6.40 | 92.60 | 1.00 |
| | $\beta_{21}$ | 1.80 | 97.80 | 0.40 | 2.20 | 94.40 | 3.40 |
| 100 | $\beta_{10}$ | 1.40 | 94.60 | 4.00 | 2.60 | 94.60 | 2.80 |
| | $\beta_{11}$ | 0.40 | 94.00 | 5.60 | 0.80 | 93.40 | 5.80 |
| | $\beta_{20}$ | 4.80 | 94.80 | 0.40 | 3.40 | 95.00 | 1.60 |
| | $\beta_{21}$ | 3.40 | 95.40 | 1.20 | 1.40 | 95.00 | 3.60 |
| 200 | $\beta_{10}$ | 2.20 | 94.00 | 3.80 | 1.40 | 94.80 | 3.80 |
| | $\beta_{11}$ | 1.60 | 93.40 | 5.00 | 1.80 | 94.80 | 3.40 |
| | $\beta_{20}$ | 4.60 | 94.40 | 1.00 | 4.40 | 94.40 | 1.20 |
| | $\beta_{21}$ | 2.60 | 96.00 | 1.40 | 1.40 | 95.60 | 3.00 |
| 500 | $\beta_{10}$ | 1.80 | 95.00 | 3.20 | 1.00 | 96.60 | 2.40 |
| | $\beta_{11}$ | 2.00 | 93.40 | 4.60 | 1.40 | 96.80 | 1.80 |
| | $\beta_{20}$ | 2.80 | 95.20 | 2.00 | 2.20 | 96.80 | 1.00 |
| | $\beta_{21}$ | 2.80 | 95.20 | 2.00 | 2.20 | 95.20 | 2.60 |

Table 18: Coverage probabilities (%) of the HPDIs using zero-inflated artificial samples (Scenarios 3 and 4).

| $n$ | Parameter | BNCP | CP | ANCP | BNCP | CP | ANCP |
|---|---|---|---|---|---|---|---|
| | | | Scenario 3 | | | Scenario 4 | |
| 50 | $\beta_{10}$ | 1.40 | 93.80 | 4.80 | 0.60 | 95.60 | 3.80 |
| | $\beta_{11}$ | 2.20 | 97.00 | 0.80 | 3.40 | 95.60 | 1.00 |
| | $\beta_{20}$ | 2.20 | 96.80 | 1.00 | 4.00 | 95.80 | 0.20 |
| | $\beta_{21}$ | 3.60 | 96.40 | 0.00 | 1.60 | 96.20 | 2.20 |
| 100 | $\beta_{10}$ | 1.40 | 95.60 | 3.00 | 1.20 | 96.60 | 2.20 |
| | $\beta_{11}$ | 2.60 | 95.80 | 1.60 | 2.00 | 96.00 | 2.00 |
| | $\beta_{20}$ | 4.20 | 95.00 | 0.80 | 3.60 | 95.40 | 1.00 |
| | $\beta_{21}$ | 1.80 | 97.40 | 0.80 | 1.00 | 97.00 | 2.00 |
| 200 | $\beta_{10}$ | 2.40 | 95.20 | 2.40 | 1.80 | 94.00 | 4.20 |
| | $\beta_{11}$ | 1.80 | 96.20 | 2.00 | 3.40 | 93.40 | 3.20 |
| | $\beta_{20}$ | 2.60 | 95.80 | 1.60 | 2.60 | 96.20 | 1.20 |
| | $\beta_{21}$ | 2.80 | 96.20 | 1.00 | 2.40 | 94.60 | 3.00 |
| 500 | $\beta_{10}$ | 2.20 | 94.40 | 3.40 | 3.00 | 94.80 | 2.20 |
| | $\beta_{11}$ | 2.00 | 95.20 | 2.80 | 1.40 | 95.40 | 3.20 |
| | $\beta_{20}$ | 3.40 | 94.80 | 1.80 | 2.80 | 96.20 | 1.00 |
| | $\beta_{21}$ | 1.60 | 96.60 | 1.80 | 1.80 | 95.40 | 2.80 |

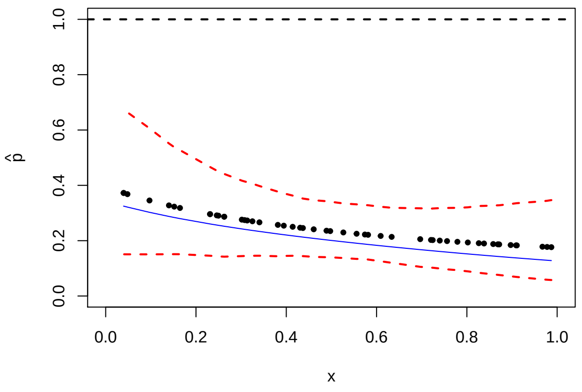


(a) $n = 50$

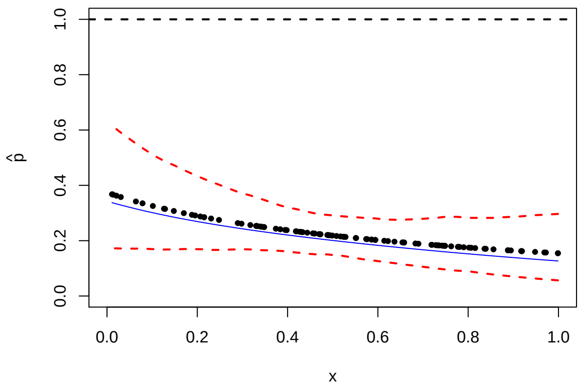


(b) $n = 100$

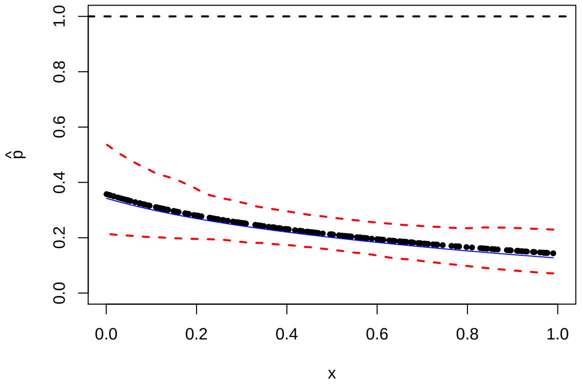


(c) $n = 200$

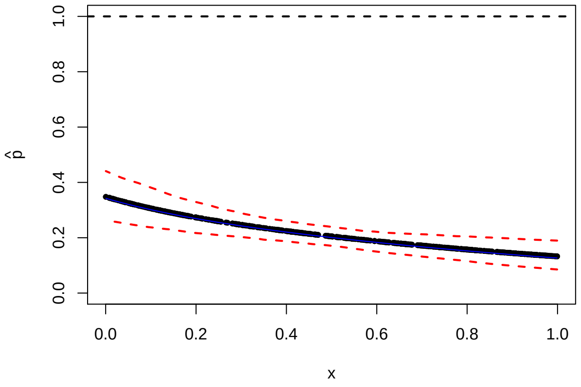


(d) $n = 500$

Figure 9: *Posterior* estimates for parameter $p$ using zero-inflated artificial samples (Scenario 1).

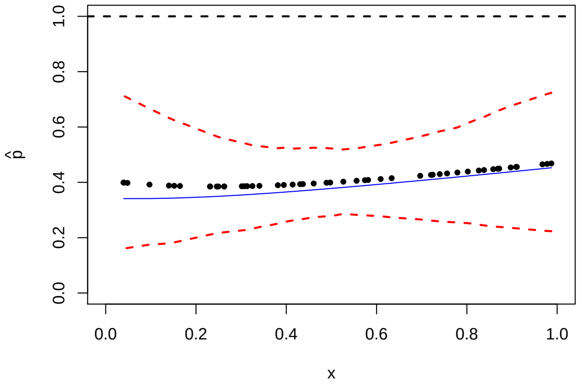


(a) $n = 50$

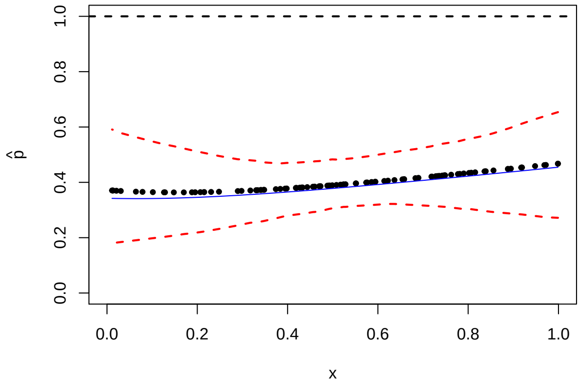


(b) $n = 100$

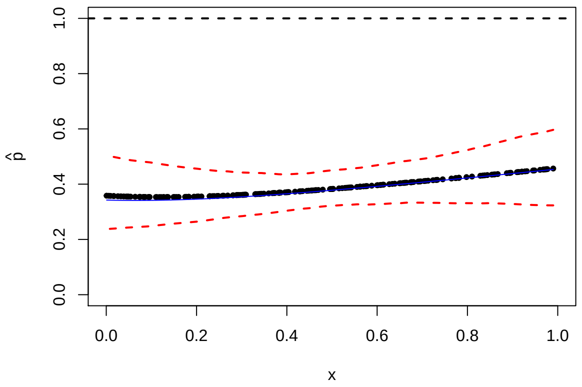


(c) $n = 200$

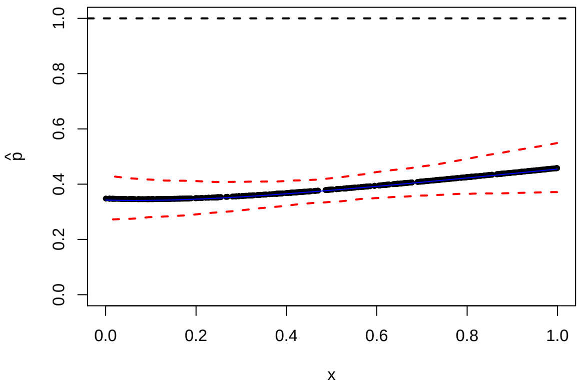


(d) $n = 500$

Figure 10: *Posterior* estimates for parameter $p$ using zero-inflated artificial samples (Scenario 2).

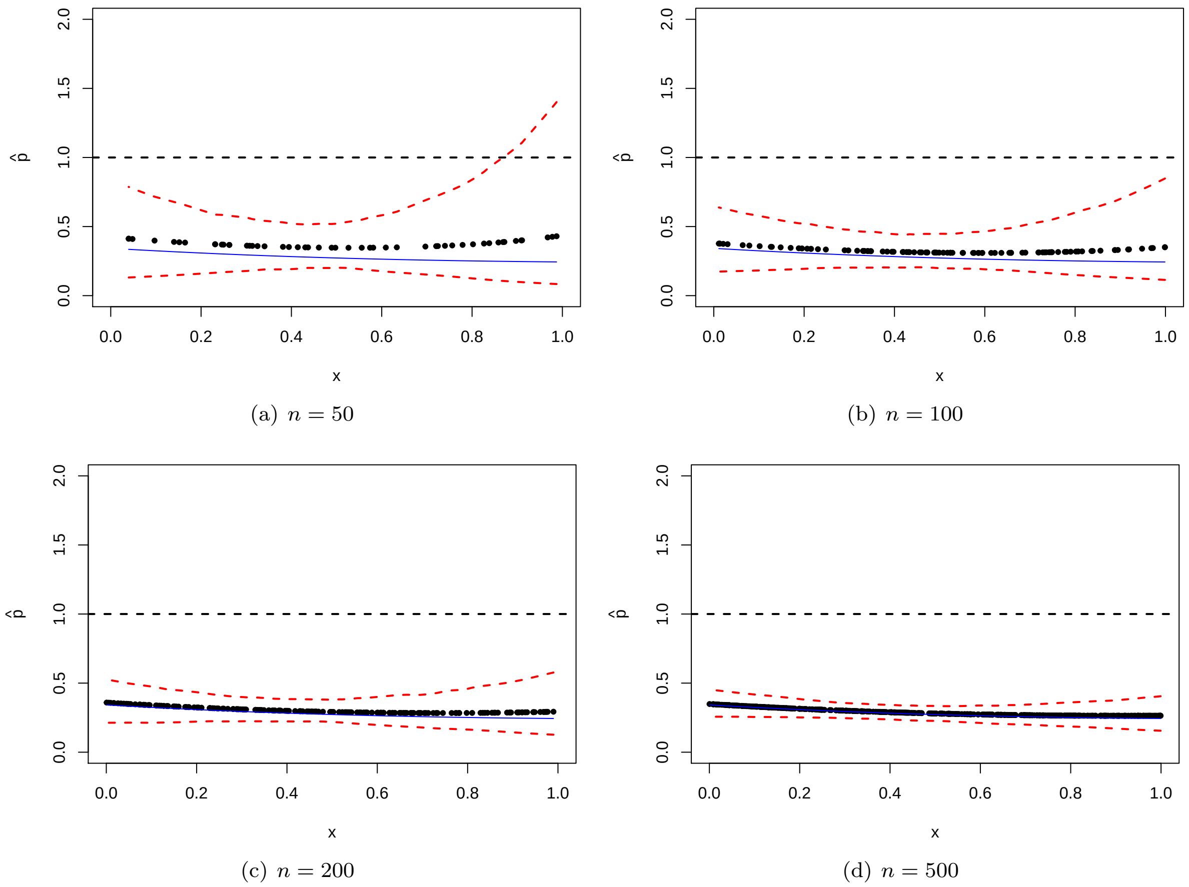


(a) $n = 50$ (b) $n = 100$

(c) $n = 200$ (d) $n = 500$

Figure 11: *Posterior* estimates for parameter $p$ using zero-inflated artificial samples (Scenario 3).

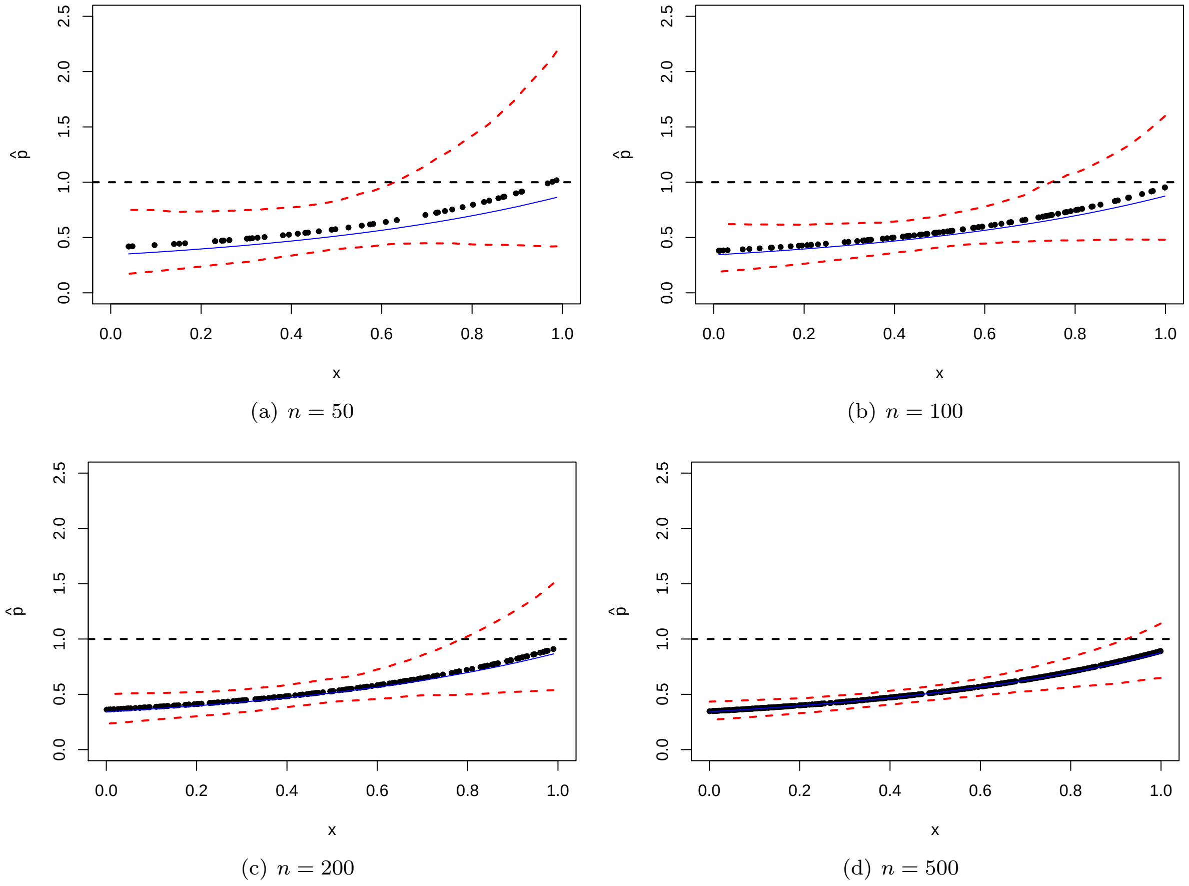


(a) $n = 50$ (b) $n = 100$

(c) $n = 200$ (d) $n = 500$

Figure 12: *Posterior* estimates for parameter $p$ using zero-inflated artificial samples (Scenario 4).

# 2 Zero-deflated artificial data

## 2.1 Using *logit* link function

Table 19: Empirical properties of the Bayesian estimators using zero-deflated artificial samples (Scenarios 1 and 2).

| $n$ | Parameter | Bias | MSE | $\sqrt{\frac{\text{MSE}}{\text{Var}}}$ | MAPE (%) |
|---|---|---|---|---|---|
| | | | Scenario 1 | | |
| 50 | $\beta_{10}$ | 0.116 | 0.342 | 1.020 | 46.269 |
| | $\beta_{11}$ | −0.209 | 0.905 | 1.025 | 75.544 |
| | $\beta_{20}$ | −0.071 | 0.298 | 1.008 | 85.289 |
| | $\beta_{21}$ | −0.017 | 0.904 | 1.000 | 148.588 |
| 100 | $\beta_{10}$ | 0.040 | 0.221 | 1.004 | 36.505 |
| | $\beta_{11}$ | −0.069 | 0.581 | 1.004 | 60.373 |
| | $\beta_{20}$ | −0.010 | 0.189 | 1.000 | 68.498 |
| | $\beta_{21}$ | −0.061 | 0.566 | 1.003 | 118.380 |
| 200 | $\beta_{10}$ | 0.043 | 0.093 | 1.010 | 25.193 |
| | $\beta_{11}$ | −0.067 | 0.268 | 1.008 | 41.512 |
| | $\beta_{20}$ | −0.016 | 0.079 | 1.002 | 44.834 |
| | $\beta_{21}$ | −0.005 | 0.277 | 1.000 | 85.763 |
| 500 | $\beta_{10}$ | −0.004 | 0.041 | 1.000 | 16.515 |
| | $\beta_{11}$ | 0.004 | 0.105 | 1.000 | 25.966 |
| | $\beta_{20}$ | 0.005 | 0.035 | 1.000 | 30.158 |
| | $\beta_{21}$ | −0.022 | 0.107 | 1.002 | 52.544 |
| | | | Scenario 2 | | |
| 50 | $\beta_{10}$ | 0.070 | 0.329 | 1.007 | 44.744 |
| | $\beta_{11}$ | −0.121 | 0.928 | 1.008 | 75.648 |
| | $\beta_{20}$ | −0.188 | 0.383 | 1.050 | 33.581 |
| | $\beta_{21}$ | 0.144 | 0.939 | 1.011 | 78.153 |
| 100 | $\beta_{10}$ | 0.040 | 0.185 | 1.004 | 33.702 |
| | $\beta_{11}$ | −0.075 | 0.508 | 1.006 | 56.059 |
| | $\beta_{20}$ | −0.069 | 0.251 | 1.010 | 27.134 |
| | $\beta_{21}$ | 0.028 | 0.648 | 1.001 | 64.126 |
| 200 | $\beta_{10}$ | 0.004 | 0.082 | 1.000 | 22.578 |
| | $\beta_{11}$ | −0.007 | 0.269 | 1.000 | 41.316 |
| | $\beta_{20}$ | −0.066 | 0.099 | 1.023 | 16.943 |
| | $\beta_{21}$ | 0.074 | 0.297 | 1.010 | 43.689 |
| 500 | $\beta_{10}$ | 0.013 | 0.038 | 1.002 | 15.487 |
| | $\beta_{11}$ | −0.021 | 0.100 | 1.002 | 25.244 |
| | $\beta_{20}$ | −0.022 | 0.045 | 1.006 | 11.579 |
| | $\beta_{21}$ | 0.018 | 0.121 | 1.001 | 27.868 |

Table 20: Empirical properties of the Bayesian estimators using zero-deflated artificial samples (Scenarios 3 and 4).

| $n$ | Parameter | Bias | MSE | $\sqrt{\frac{\text{MSE}}{\text{Var}}}$ | MAPE (%) |
|---|---|---|---|---|---|
| Scenario 3 | | | | | |
| 50 | $\beta_{10}$ | 0.007 | 0.416 | 1.000 | 51.077 |
| | $\beta_{11}$ | 0.578 | 1.484 | 1.136 | 63.871 |
| | $\beta_{20}$ | −0.046 | 0.298 | 1.004 | 88.854 |
| | $\beta_{21}$ | −0.032 | 0.908 | 1.000 | 156.233 |
| 100 | $\beta_{10}$ | 0.004 | 0.320 | 1.000 | 45.682 |
| | $\beta_{11}$ | 0.249 | 1.085 | 1.030 | 55.080 |
| | $\beta_{20}$ | −0.039 | 0.198 | 1.004 | 70.640 |
| | $\beta_{21}$ | −0.012 | 0.563 | 1.000 | 120.764 |
| 200 | $\beta_{10}$ | 0.001 | 0.155 | 1.000 | 31.560 |
| | $\beta_{11}$ | 0.142 | 0.607 | 1.017 | 41.578 |
| | $\beta_{20}$ | −0.023 | 0.080 | 1.003 | 45.237 |
| | $\beta_{21}$ | −0.004 | 0.265 | 1.000 | 81.871 |
| 500 | $\beta_{10}$ | −0.006 | 0.064 | 1.000 | 20.189 |
| | $\beta_{11}$ | 0.077 | 0.237 | 1.013 | 25.468 |
| | $\beta_{20}$ | −0.002 | 0.033 | 1.000 | 28.544 |
| | $\beta_{21}$ | −0.009 | 0.098 | 1.000 | 49.648 |
| Scenario 4 | | | | | |
| 50 | $\beta_{10}$ | −0.009 | 0.356 | 1.000 | 47.455 |
| | $\beta_{11}$ | 0.562 | 1.376 | 1.139 | 62.652 |
| | $\beta_{20}$ | −0.126 | 0.372 | 1.022 | 32.045 |
| | $\beta_{21}$ | 0.058 | 1.017 | 1.002 | 80.557 |
| 100 | $\beta_{10}$ | −0.053 | 0.294 | 1.005 | 42.976 |
| | $\beta_{11}$ | 0.366 | 1.155 | 1.064 | 56.965 |
| | $\beta_{20}$ | −0.103 | 0.239 | 1.023 | 26.300 |
| | $\beta_{21}$ | 0.113 | 0.650 | 1.010 | 65.186 |
| 200 | $\beta_{10}$ | 0.012 | 0.113 | 1.001 | 27.492 |
| | $\beta_{11}$ | 0.154 | 0.530 | 1.023 | 39.172 |
| | $\beta_{20}$ | −0.059 | 0.108 | 1.016 | 17.372 |
| | $\beta_{21}$ | 0.070 | 0.335 | 1.007 | 46.286 |
| 500 | $\beta_{10}$ | −0.013 | 0.052 | 1.002 | 17.895 |
| | $\beta_{11}$ | 0.086 | 0.207 | 1.018 | 24.165 |
| | $\beta_{20}$ | −0.013 | 0.045 | 1.002 | 11.191 |
| | $\beta_{21}$ | 0.017 | 0.122 | 1.001 | 27.718 |

Table 21: *Posterior* estimates for model parameters using zero-deflated artificial samples (Scenarios 1 and 2).

| $n$ | Parameter | Mean | Median | Std. Dev. | 95% HPDI | |
|---|---|---|---|---|---|---|
| | | | | | Lower | Upper |
| Scenario 1 | | | | | | |
| 50 | $\beta_{10}$ | −0.884 | −0.871 | 0.574 | −2.129 | 0.336 |
| | $\beta_{11}$ | 0.791 | 0.787 | 0.928 | −1.156 | 2.737 |
| | $\beta_{20}$ | 0.429 | 0.423 | 0.541 | −0.722 | 1.591 |
| | $\beta_{21}$ | 0.483 | 0.478 | 0.951 | −1.517 | 2.486 |
| 100 | $\beta_{10}$ | −0.960 | −0.952 | 0.469 | −1.917 | −0.013 |
| | $\beta_{11}$ | 0.931 | 0.928 | 0.759 | −0.590 | 2.455 |
| | $\beta_{20}$ | 0.490 | 0.485 | 0.435 | −0.399 | 1.387 |
| | $\beta_{21}$ | 0.439 | 0.437 | 0.750 | −1.119 | 1.995 |
| 200 | $\beta_{10}$ | −0.957 | −0.953 | 0.301 | −1.568 | −0.351 |
| | $\beta_{11}$ | 0.933 | 0.933 | 0.513 | −0.086 | 1.956 |
| | $\beta_{20}$ | 0.484 | 0.482 | 0.280 | −0.078 | 1.051 |
| | $\beta_{21}$ | 0.495 | 0.493 | 0.526 | −0.564 | 1.554 |
| 500 | $\beta_{10}$ | −1.004 | −1.003 | 0.203 | −1.408 | −0.602 |
| | $\beta_{11}$ | 1.004 | 1.003 | 0.324 | 0.376 | 1.633 |
| | $\beta_{20}$ | 0.505 | 0.503 | 0.187 | 0.137 | 0.874 |
| | $\beta_{21}$ | 0.478 | 0.478 | 0.327 | −0.164 | 1.121 |
| Scenario 2 | | | | | | |
| 50 | $\beta_{10}$ | −0.930 | −0.917 | 0.569 | −2.095 | 0.212 |
| | $\beta_{11}$ | 0.879 | 0.878 | 0.956 | −1.013 | 2.782 |
| | $\beta_{20}$ | 1.312 | 1.294 | 0.589 | 0.063 | 2.598 |
| | $\beta_{21}$ | −0.856 | −0.848 | 0.958 | −2.945 | 1.212 |
| 100 | $\beta_{10}$ | −0.960 | −0.953 | 0.429 | −1.852 | −0.080 |
| | $\beta_{11}$ | 0.925 | 0.923 | 0.709 | −0.548 | 2.405 |
| | $\beta_{20}$ | 1.431 | 1.420 | 0.496 | 0.445 | 2.442 |
| | $\beta_{21}$ | −0.972 | −0.964 | 0.804 | −2.626 | 0.673 |
| 200 | $\beta_{10}$ | −0.996 | −0.993 | 0.287 | −1.564 | −0.431 |
| | $\beta_{11}$ | 0.993 | 0.993 | 0.519 | −0.001 | 1.986 |
| | $\beta_{20}$ | 1.434 | 1.428 | 0.308 | 0.800 | 2.077 |
| | $\beta_{21}$ | −0.926 | −0.923 | 0.540 | −2.041 | 0.189 |
| 500 | $\beta_{10}$ | −0.987 | −0.986 | 0.194 | −1.359 | −0.618 |
| | $\beta_{11}$ | 0.979 | 0.979 | 0.315 | 0.372 | 1.587 |
| | $\beta_{20}$ | 1.478 | 1.475 | 0.211 | 1.058 | 1.902 |
| | $\beta_{21}$ | −0.982 | −0.980 | 0.347 | −1.668 | −0.300 |

Table 22: *Posterior* estimates for model parameters using zero-deflated artificial samples (Scenarios 3 and 4).

| $n$ | Parameter | Mean | Median | Std. Dev. | 95% HPDI | |
|---|---|---|---|---|---|---|
| | | | | | Lower | Upper |
| Scenario 3 | | | | | | |
| 50 | $\beta_{10}$ | −0.993 | −0.970 | 0.645 | −2.515 | 0.496 |
| | $\beta_{11}$ | −0.922 | −0.910 | 1.073 | −3.654 | 1.794 |
| | $\beta_{20}$ | 0.454 | 0.447 | 0.544 | −0.698 | 1.624 |
| | $\beta_{21}$ | 0.468 | 0.463 | 0.952 | −1.546 | 2.478 |
| 100 | $\beta_{10}$ | −0.996 | −0.980 | 0.566 | −2.206 | 0.185 |
| | $\beta_{11}$ | −1.251 | −1.244 | 1.012 | −3.489 | 0.970 |
| | $\beta_{20}$ | 0.461 | 0.457 | 0.443 | −0.427 | 1.356 |
| | $\beta_{21}$ | 0.488 | 0.486 | 0.750 | −1.067 | 2.045 |
| 200 | $\beta_{10}$ | −0.999 | −0.993 | 0.394 | −1.764 | −0.242 |
| | $\beta_{11}$ | −1.359 | −1.349 | 0.766 | −2.936 | 0.204 |
| | $\beta_{20}$ | 0.477 | 0.476 | 0.282 | −0.086 | 1.041 |
| | $\beta_{21}$ | 0.496 | 0.494 | 0.515 | −0.555 | 1.557 |
| 500 | $\beta_{10}$ | −1.006 | −1.003 | 0.252 | −1.505 | −0.512 |
| | $\beta_{11}$ | −1.423 | −1.419 | 0.480 | −2.388 | −0.466 |
| | $\beta_{20}$ | 0.498 | 0.497 | 0.182 | 0.130 | 0.868 |
| | $\beta_{21}$ | 0.491 | 0.491 | 0.312 | −0.152 | 1.134 |
| Scenario 4 | | | | | | |
| 50 | $\beta_{10}$ | −1.009 | −0.989 | 0.596 | −2.426 | 0.373 |
| | $\beta_{11}$ | −0.938 | −0.921 | 1.030 | −3.628 | 1.734 |
| | $\beta_{20}$ | 1.374 | 1.355 | 0.597 | 0.109 | 2.667 |
| | $\beta_{21}$ | −0.942 | −0.934 | 1.007 | −3.039 | 1.143 |
| 100 | $\beta_{10}$ | −1.053 | −1.040 | 0.539 | −2.169 | 0.040 |
| | $\beta_{11}$ | −1.134 | −1.127 | 1.011 | −3.277 | 1.003 |
| | $\beta_{20}$ | 1.397 | 1.386 | 0.478 | 0.412 | 2.401 |
| | $\beta_{21}$ | −0.887 | −0.880 | 0.798 | −2.538 | 0.761 |
| 200 | $\beta_{10}$ | −0.988 | −0.982 | 0.335 | −1.682 | −0.302 |
| | $\beta_{11}$ | −1.346 | −1.335 | 0.711 | −2.861 | 0.154 |
| | $\beta_{20}$ | 1.441 | 1.436 | 0.322 | 0.805 | 2.084 |
| | $\beta_{21}$ | −0.930 | −0.928 | 0.574 | −2.045 | 0.189 |
| 500 | $\beta_{10}$ | −1.013 | −1.010 | 0.227 | −1.468 | −0.563 |
| | $\beta_{11}$ | −1.414 | −1.410 | 0.447 | −2.350 | −0.485 |
| | $\beta_{20}$ | 1.487 | 1.484 | 0.213 | 1.066 | 1.912 |
| | $\beta_{21}$ | −0.983 | −0.981 | 0.348 | −1.670 | −0.299 |

Table 23: Coverage probabilities (%) of the HPDIs using zero-deflated artificial samples (Scenarios 1 and 2).

| $n$ | Parameter | BNCP | CP | ANCP | BNCP | CP | ANCP |
|---|---|---|---|---|---|---|---|
| | | | Scenario 1 | | | Scenario 2 | |
| 50 | $\beta_{10}$ | 3.40 | 96.20 | 0.40 | 4.40 | 94.40 | 1.20 |
| | $\beta_{11}$ | 0.80 | 96.60 | 2.60 | 2.20 | 95.20 | 2.60 |
| | $\beta_{20}$ | 0.40 | 97.00 | 2.60 | 0.20 | 95.60 | 4.20 |
| | $\beta_{21}$ | 1.40 | 97.60 | 1.00 | 2.40 | 97.40 | 0.20 |
| 100 | $\beta_{10}$ | 3.80 | 94.60 | 1.60 | 2.20 | 96.80 | 1.00 |
| | $\beta_{11}$ | 1.60 | 96.40 | 2.00 | 1.60 | 95.60 | 2.80 |
| | $\beta_{20}$ | 1.60 | 96.00 | 2.40 | 0.80 | 95.20 | 4.00 |
| | $\beta_{21}$ | 1.40 | 96.20 | 2.40 | 2.60 | 96.00 | 1.40 |
| 200 | $\beta_{10}$ | 3.40 | 96.20 | 0.40 | 3.00 | 95.00 | 2.00 |
| | $\beta_{11}$ | 1.20 | 96.60 | 2.20 | 3.80 | 93.60 | 2.60 |
| | $\beta_{20}$ | 0.80 | 96.00 | 3.20 | 0.80 | 96.00 | 3.20 |
| | $\beta_{21}$ | 1.20 | 97.00 | 1.80 | 2.40 | 96.40 | 1.20 |
| 500 | $\beta_{10}$ | 2.20 | 95.00 | 2.80 | 2.60 | 95.20 | 2.20 |
| | $\beta_{11}$ | 2.40 | 94.80 | 2.80 | 2.00 | 95.60 | 2.40 |
| | $\beta_{20}$ | 0.80 | 95.80 | 3.40 | 1.40 | 95.20 | 3.40 |
| | $\beta_{21}$ | 2.40 | 95.80 | 1.80 | 2.80 | 95.00 | 2.20 |

Table 24: Coverage probabilities (%) of the HPDIs using zero-deflated artificial samples (Scenarios 3 and 4).

| $n$ | Parameter | BNCP | CP | ANCP | BNCP | CP | ANCP |
|---|---|---|---|---|---|---|---|
| | | | Scenario 3 | | | Scenario 4 | |
| 50 | $\beta_{10}$ | 2.20 | 97.60 | 0.20 | 1.40 | 98.60 | 0.00 |
| | $\beta_{11}$ | 3.20 | 96.80 | 0.00 | 2.60 | 97.40 | 0.00 |
| | $\beta_{20}$ | 0.40 | 97.60 | 2.00 | 1.00 | 96.00 | 3.00 |
| | $\beta_{21}$ | 0.80 | 97.60 | 1.60 | 2.00 | 96.60 | 1.40 |
| 100 | $\beta_{10}$ | 2.40 | 97.00 | 0.60 | 1.20 | 97.60 | 1.20 |
| | $\beta_{11}$ | 1.80 | 97.60 | 0.60 | 3.40 | 96.40 | 0.20 |
| | $\beta_{20}$ | 2.00 | 95.00 | 3.00 | 0.80 | 94.60 | 4.60 |
| | $\beta_{21}$ | 1.60 | 96.40 | 2.00 | 2.80 | 96.20 | 1.00 |
| 200 | $\beta_{10}$ | 3.40 | 94.40 | 2.20 | 3.40 | 95.80 | 0.80 |
| | $\beta_{11}$ | 4.00 | 95.20 | 0.80 | 2.80 | 96.20 | 1.00 |
| | $\beta_{20}$ | 2.20 | 95.60 | 2.20 | 2.00 | 94.80 | 3.20 |
| | $\beta_{21}$ | 1.20 | 96.80 | 2.00 | 3.60 | 94.40 | 2.00 |
| 500 | $\beta_{10}$ | 3.40 | 94.60 | 2.00 | 2.20 | 94.80 | 3.00 |
| | $\beta_{11}$ | 4.00 | 94.40 | 1.60 | 3.40 | 95.80 | 0.80 |
| | $\beta_{20}$ | 2.20 | 94.60 | 3.20 | 1.20 | 95.20 | 3.60 |
| | $\beta_{21}$ | 1.40 | 96.80 | 1.80 | 3.20 | 95.40 | 1.40 |

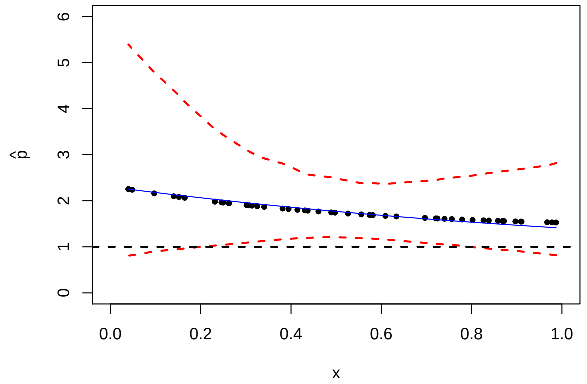


(a) $n = 50$

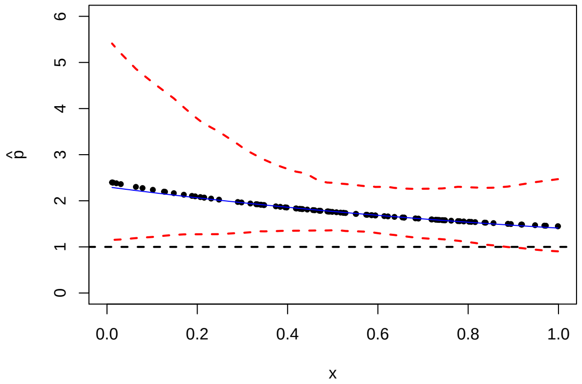


(b) $n = 100$

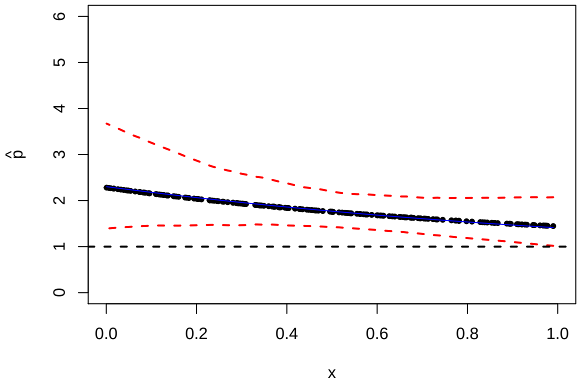


(c) $n = 200$

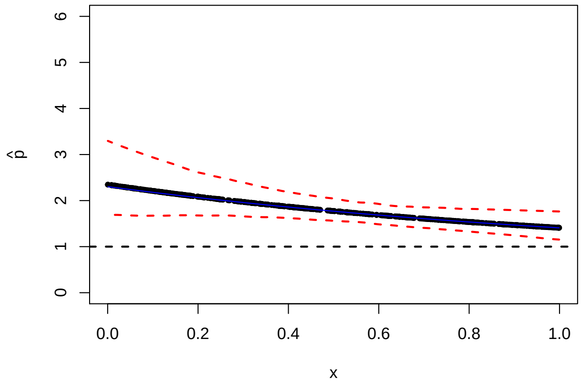


(d) $n = 500$

Figure 13: *Posterior* estimates for parameter $p$ using zero-deflated artificial samples (Scenario 1).

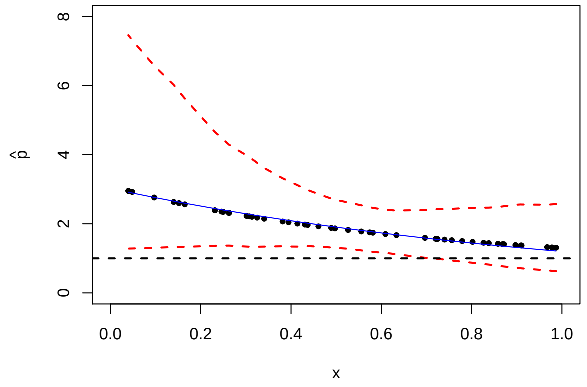


(a) $n = 50$

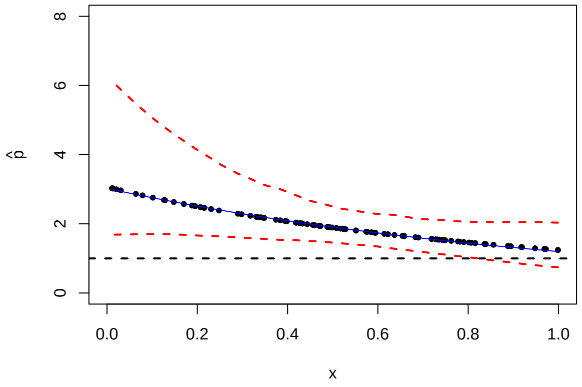


(b) $n = 100$

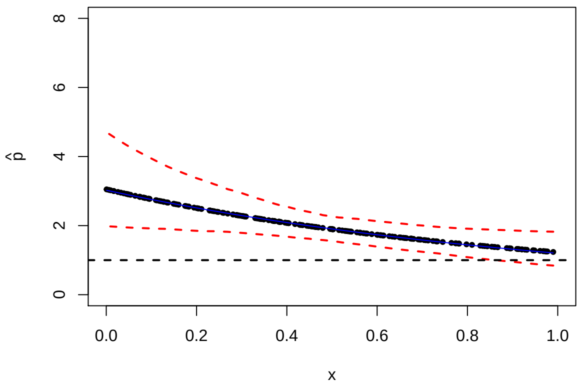


(c) $n = 200$

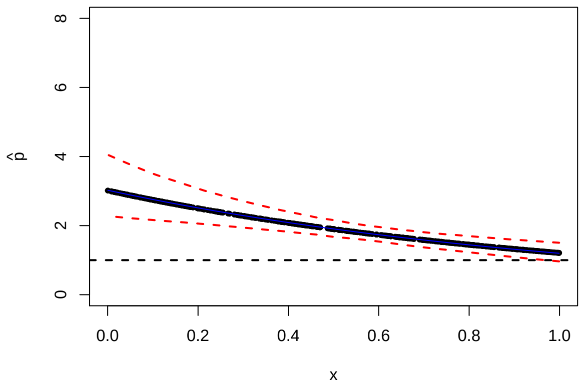


(d) $n = 500$

Figure 14: *Posterior* estimates for parameter $p$ using zero-deflated artificial samples (Scenario 2).

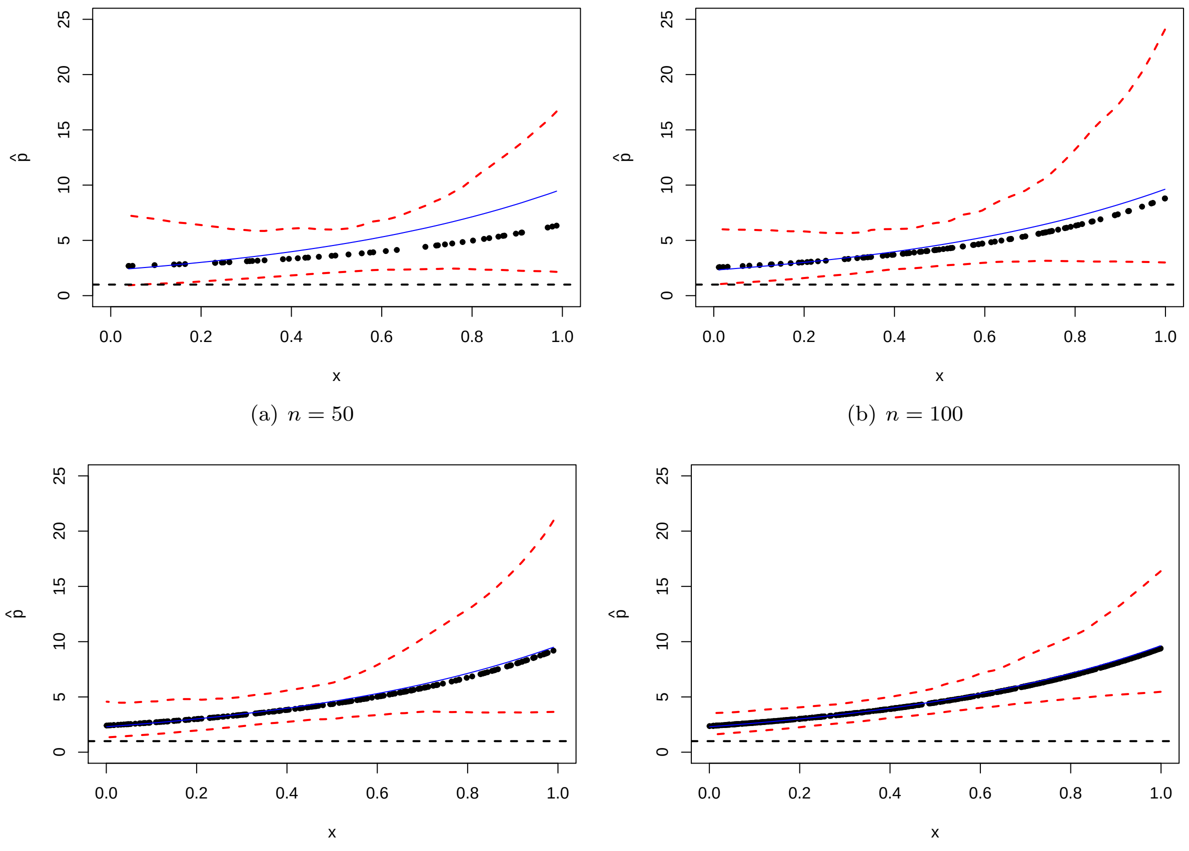


(a) $n = 50$ (b) $n = 100$

(c) $n = 200$ (d) $n = 500$

Figure 15: *Posterior* estimates for parameter $p$ using zero-deflated artificial samples (Scenario 3).

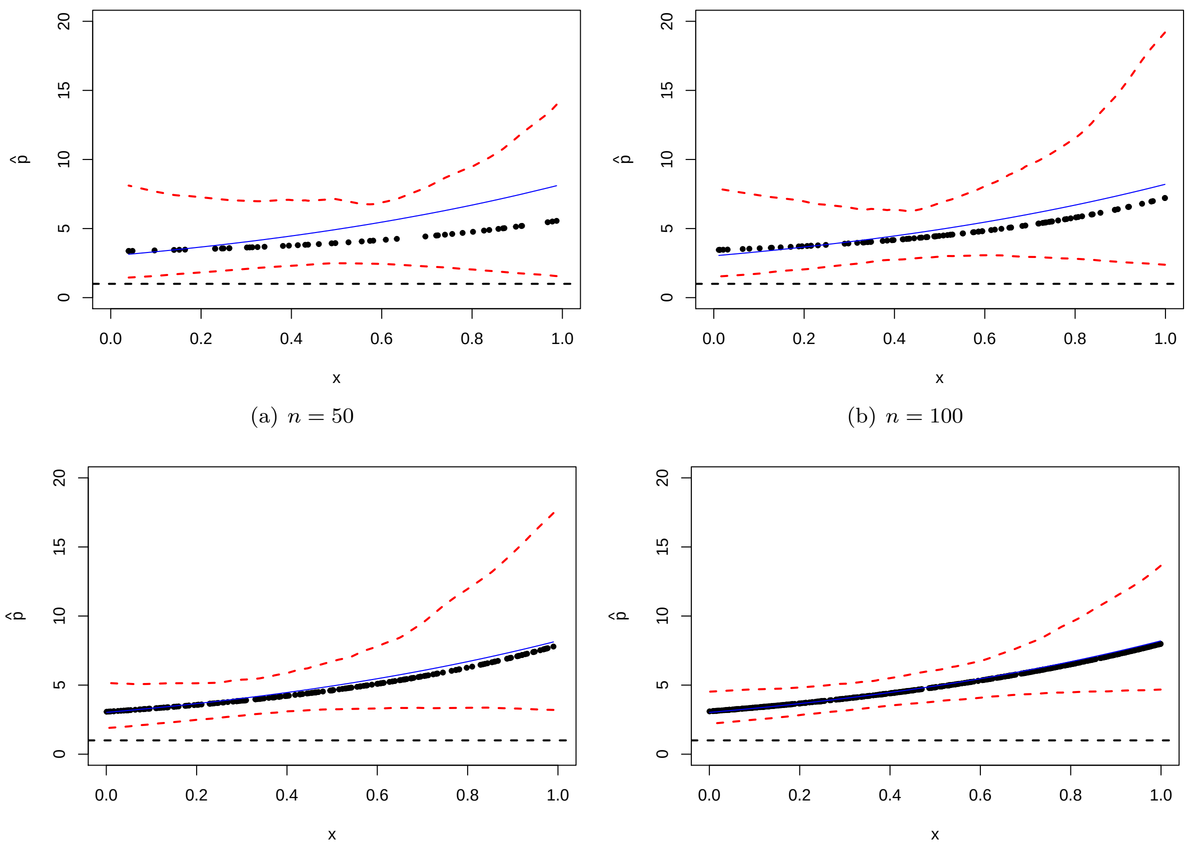


(a) $n = 50$ (b) $n = 100$

(c) $n = 200$ (d) $n = 500$

Figure 16: *Posterior* estimates for parameter $p$ using zero-deflated artificial samples (Scenario 4).

## 2.2 Using *probit* link function

Table 25: Empirical properties of the Bayesian estimators using zero-deflated artificial samples (Scenarios 1 and 2).

| $n$ | Parameter | Bias | MSE | $\sqrt{\frac{\text{MSE}}{\text{Var}}}$ | MAPE (%) |
|---|---|---|---|---|---|
| | | | Scenario 1 | | |
| 50 | $\beta_{10}$ | 0.081 | 0.324 | 1.010 | 45.581 |
| | $\beta_{11}$ | −0.164 | 0.839 | 1.016 | 74.955 |
| | $\beta_{20}$ | −0.001 | 0.156 | 1.000 | 62.082 |
| | $\beta_{21}$ | −0.050 | 0.471 | 1.003 | 109.956 |
| 100 | $\beta_{10}$ | 0.053 | 0.197 | 1.007 | 35.633 |
| | $\beta_{11}$ | −0.075 | 0.485 | 1.006 | 55.800 |
| | $\beta_{20}$ | −0.034 | 0.092 | 1.006 | 47.660 |
| | $\beta_{21}$ | 0.030 | 0.301 | 1.002 | 86.294 |
| 200 | $\beta_{10}$ | 0.012 | 0.078 | 1.001 | 22.714 |
| | $\beta_{11}$ | −0.017 | 0.231 | 1.001 | 38.300 |
| | $\beta_{20}$ | −0.022 | 0.035 | 1.007 | 29.615 |
| | $\beta_{21}$ | 0.020 | 0.123 | 1.002 | 55.809 |
| 500 | $\beta_{10}$ | 0.016 | 0.040 | 1.003 | 15.990 |
| | $\beta_{11}$ | −0.028 | 0.098 | 1.004 | 25.124 |
| | $\beta_{20}$ | 0.006 | 0.016 | 1.001 | 19.987 |
| | $\beta_{21}$ | −0.011 | 0.047 | 1.001 | 35.119 |
| | | | Scenario 2 | | |
| 50 | $\beta_{10}$ | 0.050 | 0.266 | 1.005 | 41.381 |
| | $\beta_{11}$ | −0.084 | 0.744 | 1.005 | 67.351 |
| | $\beta_{20}$ | −0.072 | 0.205 | 1.013 | 24.018 |
| | $\beta_{21}$ | 0.057 | 0.512 | 1.003 | 56.514 |
| 100 | $\beta_{10}$ | 0.012 | 0.171 | 1.000 | 32.812 |
| | $\beta_{11}$ | −0.022 | 0.445 | 1.001 | 52.762 |
| | $\beta_{20}$ | −0.014 | 0.138 | 1.001 | 19.169 |
| | $\beta_{21}$ | 0.002 | 0.380 | 1.000 | 48.545 |
| 200 | $\beta_{10}$ | 0.010 | 0.072 | 1.001 | 21.244 |
| | $\beta_{11}$ | −0.020 | 0.214 | 1.001 | 37.192 |
| | $\beta_{20}$ | −0.031 | 0.043 | 1.012 | 11.013 |
| | $\beta_{21}$ | 0.042 | 0.136 | 1.007 | 29.241 |
| 500 | $\beta_{10}$ | 0.019 | 0.028 | 1.007 | 13.449 |
| | $\beta_{11}$ | −0.028 | 0.074 | 1.005 | 21.794 |
| | $\beta_{20}$ | 0.002 | 0.026 | 1.000 | 8.697 |
| | $\beta_{21}$ | −0.004 | 0.061 | 1.000 | 19.548 |

Table 26: Empirical properties of the Bayesian estimators using zero-deflated artificial samples (Scenarios 3 and 4).

| $n$ | Parameter | Bias | MSE | $\sqrt{\frac{\text{MSE}}{\text{Var}}}$ | MAPE (%) |
|---|---|---|---|---|---|
| Scenario 3 | | | | | |
| 50 | $\beta_{10}$ | 0.018 | 0.371 | 1.000 | 47.775 |
| | $\beta_{11}$ | 0.463 | 1.251 | 1.099 | 58.669 |
| | $\beta_{20}$ | −0.019 | 0.146 | 1.001 | 60.599 |
| | $\beta_{21}$ | −0.040 | 0.446 | 1.002 | 104.663 |
| 100 | $\beta_{10}$ | −0.003 | 0.287 | 1.000 | 43.011 |
| | $\beta_{11}$ | 0.253 | 0.969 | 1.035 | 51.804 |
| | $\beta_{20}$ | −0.020 | 0.079 | 1.002 | 44.592 |
| | $\beta_{21}$ | 0.017 | 0.253 | 1.001 | 79.059 |
| 200 | $\beta_{10}$ | −0.028 | 0.125 | 1.003 | 27.594 |
| | $\beta_{11}$ | 0.159 | 0.539 | 1.024 | 38.997 |
| | $\beta_{20}$ | −0.003 | 0.035 | 1.000 | 29.779 |
| | $\beta_{21}$ | 0.006 | 0.136 | 1.000 | 56.819 |
| 500 | $\beta_{10}$ | −0.005 | 0.058 | 1.000 | 19.370 |
| | $\beta_{11}$ | 0.054 | 0.223 | 1.007 | 25.061 |
| | $\beta_{20}$ | −0.002 | 0.014 | 1.000 | 19.111 |
| | $\beta_{21}$ | −0.006 | 0.046 | 1.000 | 34.241 |
| Scenario 4 | | | | | |
| 50 | $\beta_{10}$ | −0.076 | 0.372 | 1.008 | 48.160 |
| | $\beta_{11}$ | 0.624 | 1.415 | 1.174 | 62.839 |
| | $\beta_{20}$ | −0.069 | 0.215 | 1.011 | 25.209 |
| | $\beta_{21}$ | 0.034 | 0.546 | 1.001 | 58.479 |
| 100 | $\beta_{10}$ | −0.051 | 0.231 | 1.006 | 38.240 |
| | $\beta_{11}$ | 0.352 | 0.930 | 1.074 | 51.961 |
| | $\beta_{20}$ | 0.013 | 0.119 | 1.001 | 17.847 |
| | $\beta_{21}$ | −0.050 | 0.320 | 1.004 | 44.541 |
| 200 | $\beta_{10}$ | 0.005 | 0.111 | 1.000 | 25.939 |
| | $\beta_{11}$ | 0.133 | 0.544 | 1.017 | 39.311 |
| | $\beta_{20}$ | −0.010 | 0.059 | 1.001 | 12.777 |
| | $\beta_{21}$ | 0.001 | 0.163 | 1.000 | 32.126 |
| 500 | $\beta_{10}$ | −0.001 | 0.045 | 1.000 | 17.131 |
| | $\beta_{11}$ | 0.079 | 0.185 | 1.017 | 23.264 |
| | $\beta_{20}$ | −0.011 | 0.022 | 1.003 | 8.025 |
| | $\beta_{21}$ | 0.014 | 0.054 | 1.002 | 18.553 |

Table 27: *Posterior* estimates for model parameters using zero-deflated artificial samples (Scenarios 1 and 2).

| $n$ | Parameter | Mean | Median | Std. Dev. | 95% HPDI | |
|---|---|---|---|---|---|---|
| | | | | | Lower | Upper |
| Scenario 1 | | | | | | |
| 50 | $\beta_{10}$ | −0.919 | −0.906 | 0.563 | −2.094 | 0.239 |
| | $\beta_{11}$ | 0.836 | 0.832 | 0.901 | −0.993 | 2.668 |
| | $\beta_{20}$ | 0.499 | 0.495 | 0.394 | −0.267 | 1.276 |
| | $\beta_{21}$ | 0.450 | 0.446 | 0.684 | −0.905 | 1.811 |
| 100 | $\beta_{10}$ | −0.947 | −0.940 | 0.440 | −1.850 | −0.054 |
| | $\beta_{11}$ | 0.925 | 0.922 | 0.693 | −0.500 | 2.357 |
| | $\beta_{20}$ | 0.466 | 0.464 | 0.302 | −0.114 | 1.047 |
| | $\beta_{21}$ | 0.530 | 0.528 | 0.548 | −0.501 | 1.560 |
| 200 | $\beta_{10}$ | −0.988 | −0.985 | 0.280 | −1.567 | −0.412 |
| | $\beta_{11}$ | 0.983 | 0.982 | 0.480 | 0.025 | 1.945 |
| | $\beta_{20}$ | 0.478 | 0.477 | 0.186 | 0.114 | 0.844 |
| | $\beta_{21}$ | 0.520 | 0.518 | 0.350 | −0.176 | 1.214 |
| 500 | $\beta_{10}$ | −0.984 | −0.983 | 0.199 | −1.362 | −0.608 |
| | $\beta_{11}$ | 0.972 | 0.972 | 0.311 | 0.385 | 1.560 |
| | $\beta_{20}$ | 0.506 | 0.506 | 0.125 | 0.269 | 0.744 |
| | $\beta_{21}$ | 0.489 | 0.489 | 0.217 | 0.068 | 0.909 |
| Scenario 2 | | | | | | |
| 50 | $\beta_{10}$ | −0.950 | −0.938 | 0.514 | −2.034 | 0.121 |
| | $\beta_{11}$ | 0.916 | 0.914 | 0.858 | −0.855 | 2.694 |
| | $\beta_{20}$ | 1.428 | 1.410 | 0.447 | 0.496 | 2.388 |
| | $\beta_{21}$ | −0.943 | −0.931 | 0.713 | −2.445 | 0.534 |
| 100 | $\beta_{10}$ | −0.988 | −0.981 | 0.413 | −1.826 | −0.160 |
| | $\beta_{11}$ | 0.978 | 0.976 | 0.667 | −0.406 | 2.370 |
| | $\beta_{20}$ | 1.486 | 1.477 | 0.371 | 0.771 | 2.218 |
| | $\beta_{21}$ | −0.998 | −0.991 | 0.616 | −2.162 | 0.154 |
| 200 | $\beta_{10}$ | −0.990 | −0.987 | 0.268 | −1.521 | −0.462 |
| | $\beta_{11}$ | 0.980 | 0.981 | 0.463 | 0.048 | 1.915 |
| | $\beta_{20}$ | 1.469 | 1.465 | 0.205 | 1.017 | 1.924 |
| | $\beta_{21}$ | −0.958 | −0.956 | 0.366 | −1.726 | −0.194 |
| 500 | $\beta_{10}$ | −0.981 | −0.980 | 0.167 | −1.327 | −0.636 |
| | $\beta_{11}$ | 0.972 | 0.972 | 0.270 | 0.403 | 1.540 |
| | $\beta_{20}$ | 1.502 | 1.500 | 0.163 | 1.204 | 1.803 |
| | $\beta_{21}$ | −1.004 | −1.003 | 0.246 | −1.475 | −0.536 |

Table 28: *Posterior* estimates for model parameters using zero-deflated artificial samples (Scenarios 3 and 4).

| $n$ | Parameter | Mean | Median | Std. Dev. | 95% HPDI Lower | 95% HPDI Upper |
|---|---|---|---|---|---|---|
| Scenario 3 | | | | | | |
| 50 | $\beta_{10}$ | −0.982 | −0.960 | 0.609 | −2.438 | 0.431 |
| | $\beta_{11}$ | −1.037 | −1.023 | 1.018 | −3.661 | 1.571 |
| | $\beta_{20}$ | 0.481 | 0.477 | 0.381 | −0.284 | 1.248 |
| | $\beta_{21}$ | 0.460 | 0.456 | 0.666 | −0.883 | 1.816 |
| 100 | $\beta_{10}$ | −1.003 | −0.989 | 0.536 | −2.138 | 0.113 |
| | $\beta_{11}$ | −1.247 | −1.240 | 0.951 | −3.347 | 0.841 |
| | $\beta_{20}$ | 0.480 | 0.479 | 0.281 | −0.100 | 1.063 |
| | $\beta_{21}$ | 0.517 | 0.515 | 0.503 | −0.514 | 1.551 |
| 200 | $\beta_{10}$ | −1.028 | −1.022 | 0.352 | −1.750 | −0.312 |
| | $\beta_{11}$ | −1.341 | −1.332 | 0.717 | −2.829 | 0.126 |
| | $\beta_{20}$ | 0.497 | 0.496 | 0.187 | 0.133 | 0.863 |
| | $\beta_{21}$ | 0.506 | 0.504 | 0.368 | −0.188 | 1.203 |
| 500 | $\beta_{10}$ | −1.005 | −1.002 | 0.240 | −1.475 | −0.536 |
| | $\beta_{11}$ | −1.446 | −1.443 | 0.470 | −2.354 | −0.540 |
| | $\beta_{20}$ | 0.498 | 0.498 | 0.120 | 0.261 | 0.736 |
| | $\beta_{21}$ | 0.494 | 0.494 | 0.214 | 0.074 | 0.913 |
| Scenario 4 | | | | | | |
| 50 | $\beta_{10}$ | −1.076 | −1.056 | 0.605 | −2.432 | 0.241 |
| | $\beta_{11}$ | −0.876 | −0.862 | 1.013 | −3.440 | 1.667 |
| | $\beta_{20}$ | 1.431 | 1.414 | 0.459 | 0.503 | 2.389 |
| | $\beta_{21}$ | −0.966 | −0.956 | 0.738 | −2.459 | 0.509 |
| 100 | $\beta_{10}$ | −1.051 | −1.039 | 0.478 | −2.089 | −0.028 |
| | $\beta_{11}$ | −1.147 | −1.140 | 0.898 | −3.168 | 0.856 |
| | $\beta_{20}$ | 1.513 | 1.503 | 0.345 | 0.795 | 2.250 |
| | $\beta_{21}$ | −1.050 | −1.043 | 0.563 | −2.218 | 0.105 |
| 200 | $\beta_{10}$ | −0.995 | −0.990 | 0.332 | −1.645 | −0.353 |
| | $\beta_{11}$ | −1.367 | −1.356 | 0.725 | −2.797 | 0.049 |
| | $\beta_{20}$ | 1.490 | 1.486 | 0.242 | 1.034 | 1.952 |
| | $\beta_{21}$ | −0.999 | −0.997 | 0.404 | −1.771 | −0.233 |
| 500 | $\beta_{10}$ | −1.001 | −0.999 | 0.212 | −1.425 | −0.581 |
| | $\beta_{11}$ | −1.421 | −1.418 | 0.423 | −2.297 | −0.552 |
| | $\beta_{20}$ | 1.489 | 1.487 | 0.150 | 1.192 | 1.789 |
| | $\beta_{21}$ | −0.986 | −0.984 | 0.233 | −1.456 | −0.518 |

Table 29: Coverage probabilities (%) of the HPDIs using zero-deflated artificial samples (Scenarios 1 and 2).

| $n$ | Parameter | BNCP | CP | ANCP | BNCP | CP | ANCP |
|---|---|---|---|---|---|---|---|
| | | Scenario 1 | | | Scenario 2 | | |
| 50 | $\beta_{10}$ | 4.60 | 94.80 | 0.60 | 3.40 | 96.20 | 0.40 |
| | $\beta_{11}$ | 0.80 | 96.80 | 2.40 | 1.60 | 95.80 | 2.60 |
| | $\beta_{20}$ | 1.60 | 95.80 | 2.60 | 0.00 | 97.00 | 3.00 |
| | $\beta_{21}$ | 1.40 | 96.00 | 2.60 | 1.60 | 97.80 | 0.60 |
| 100 | $\beta_{10}$ | 3.60 | 95.20 | 1.20 | 2.60 | 96.40 | 1.00 |
| | $\beta_{11}$ | 0.80 | 96.00 | 3.20 | 1.60 | 96.60 | 1.80 |
| | $\beta_{20}$ | 1.60 | 95.40 | 3.00 | 1.60 | 94.20 | 4.20 |
| | $\beta_{21}$ | 2.80 | 94.40 | 2.80 | 3.40 | 94.60 | 2.00 |
| 200 | $\beta_{10}$ | 2.60 | 96.20 | 1.20 | 2.80 | 95.40 | 1.80 |
| | $\beta_{11}$ | 2.20 | 96.00 | 1.80 | 1.60 | 96.20 | 2.20 |
| | $\beta_{20}$ | 1.80 | 94.80 | 3.40 | 0.80 | 95.60 | 3.60 |
| | $\beta_{21}$ | 1.80 | 95.40 | 2.80 | 3.40 | 95.40 | 1.20 |
| 500 | $\beta_{10}$ | 4.60 | 93.60 | 1.80 | 4.00 | 94.80 | 1.20 |
| | $\beta_{11}$ | 2.00 | 94.40 | 3.60 | 1.00 | 96.60 | 2.40 |
| | $\beta_{20}$ | 1.80 | 95.40 | 2.80 | 3.00 | 94.00 | 3.00 |
| | $\beta_{21}$ | 3.20 | 94.60 | 2.20 | 2.60 | 94.20 | 3.20 |

Table 30: Coverage probabilities (%) of the HPDIs using zero-deflated artificial samples (Scenarios 3 and 4).

| $n$ | Parameter | BNCP | CP | ANCP | BNCP | CP | ANCP |
|---|---|---|---|---|---|---|---|
| | | Scenario 3 | | | Scenario 4 | | |
| 50 | $\beta_{10}$ | 1.80 | 98.00 | 0.20 | 1.00 | 98.00 | 1.00 |
| | $\beta_{11}$ | 1.80 | 98.00 | 0.20 | 3.80 | 96.20 | 0.00 |
| | $\beta_{20}$ | 1.40 | 95.60 | 3.00 | 0.40 | 96.00 | 3.60 |
| | $\beta_{21}$ | 1.40 | 96.00 | 2.60 | 3.20 | 95.60 | 1.20 |
| 100 | $\beta_{10}$ | 1.40 | 98.20 | 0.40 | 2.00 | 97.20 | 0.80 |
| | $\beta_{11}$ | 2.60 | 97.00 | 0.40 | 2.20 | 97.20 | 0.60 |
| | $\beta_{20}$ | 1.80 | 96.40 | 1.80 | 0.60 | 97.00 | 2.40 |
| | $\beta_{21}$ | 2.00 | 95.60 | 2.40 | 1.40 | 96.80 | 1.80 |
| 200 | $\beta_{10}$ | 2.60 | 95.80 | 1.60 | 4.00 | 93.40 | 2.60 |
| | $\beta_{11}$ | 3.20 | 96.20 | 0.60 | 5.60 | 93.40 | 1.00 |
| | $\beta_{20}$ | 2.40 | 95.40 | 2.20 | 2.00 | 94.60 | 3.40 |
| | $\beta_{21}$ | 2.40 | 94.40 | 3.20 | 2.60 | 94.60 | 2.80 |
| 500 | $\beta_{10}$ | 2.20 | 95.60 | 2.20 | 3.20 | 95.60 | 1.20 |
| | $\beta_{11}$ | 4.00 | 94.00 | 2.00 | 2.40 | 96.80 | 0.80 |
| | $\beta_{20}$ | 3.60 | 94.80 | 1.60 | 1.40 | 94.40 | 4.20 |
| | $\beta_{21}$ | 2.40 | 94.40 | 3.20 | 2.40 | 95.60 | 2.00 |

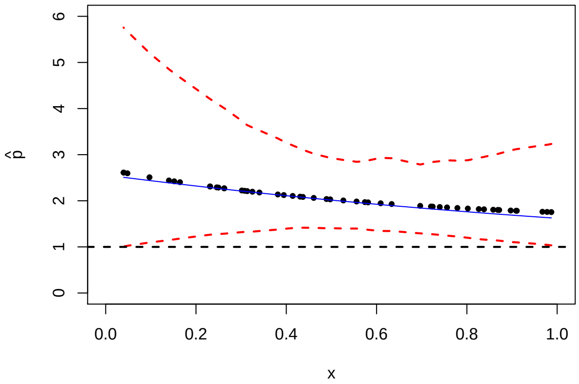


(a) $n = 50$

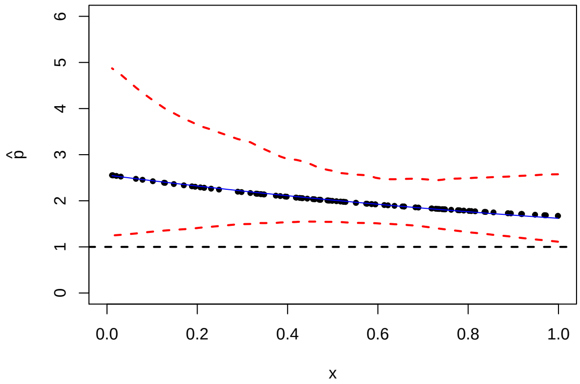


(b) $n = 100$

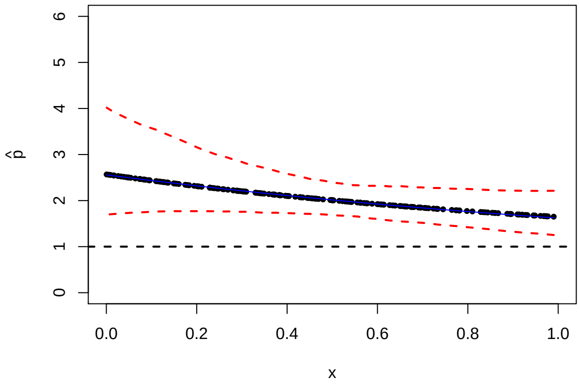


(c) $n = 200$

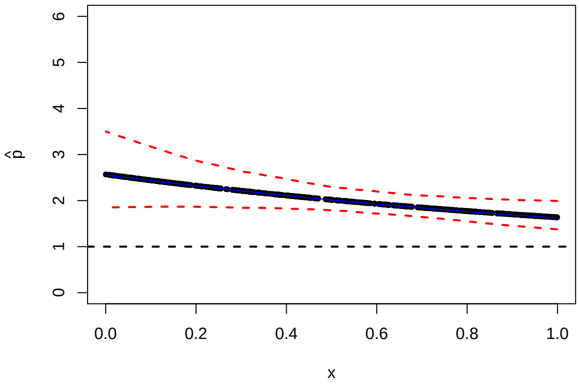


(d) $n = 500$

Figure 17: *Posterior* estimates for parameter $p$ using zero-deflated artificial samples (Scenario 1).

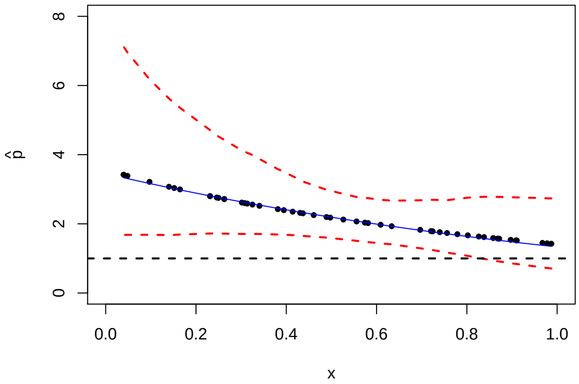


(a) $n = 50$

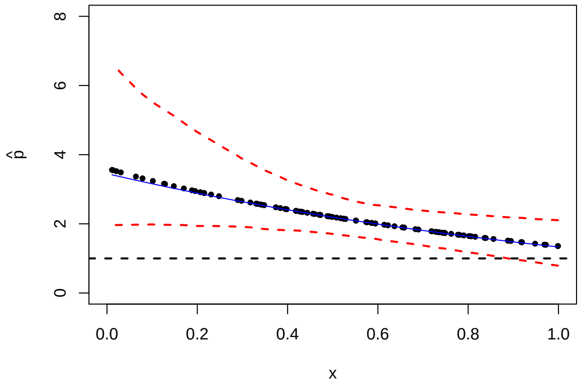


(b) $n = 100$

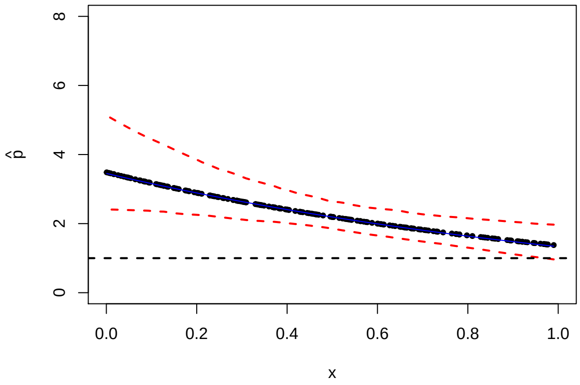


(c) $n = 200$

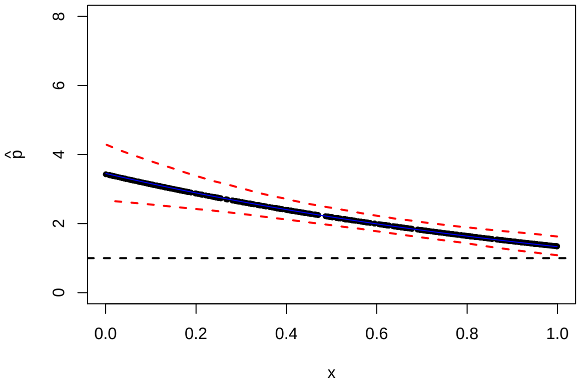


(d) $n = 500$

Figure 18: *Posterior* estimates for parameter $p$ using zero-deflated artificial samples (Scenario 2).

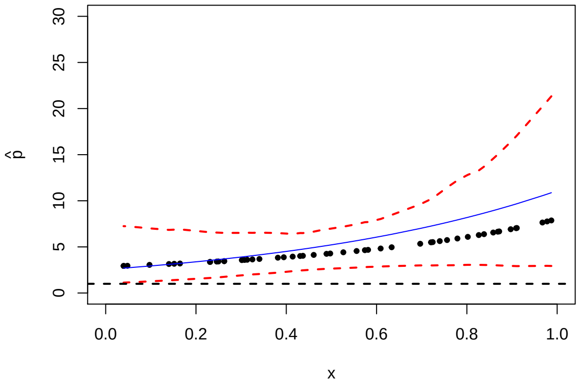


(a) $n = 50$

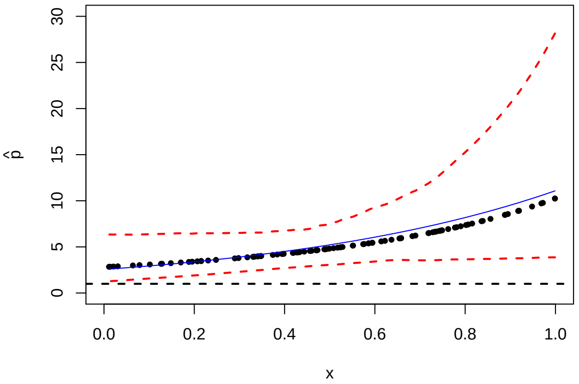


(b) $n = 100$

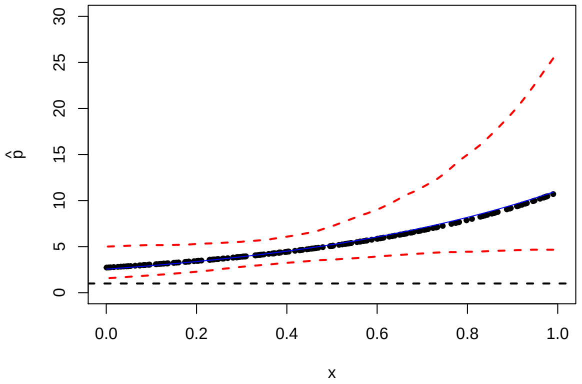


(c) $n = 200$

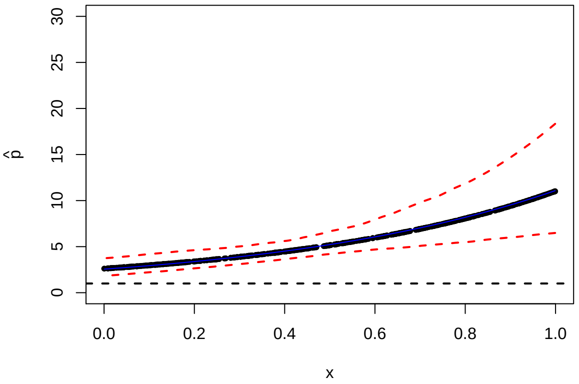


(d) $n = 500$

Figure 19: *Posterior* estimates for parameter $p$ using zero-deflated artificial samples (Scenario 3).

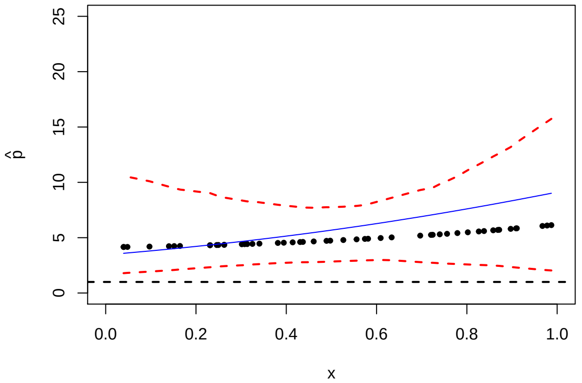


(a) $n = 50$

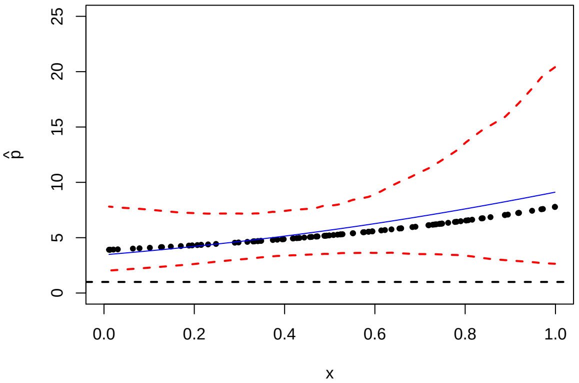


(b) $n = 100$

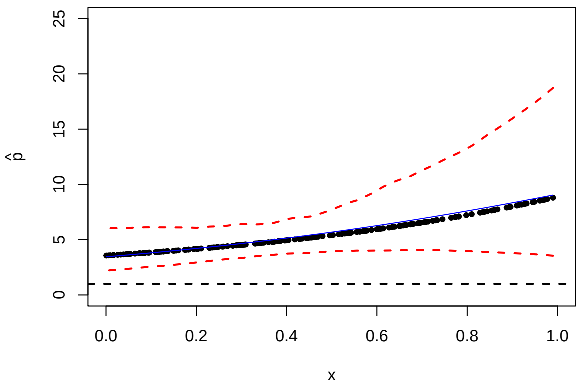


(c) $n = 200$

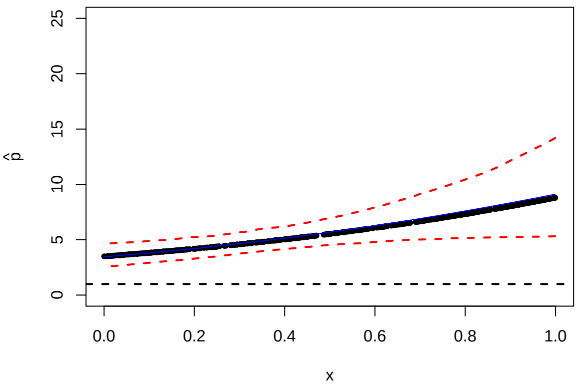


(d) $n = 500$

Figure 20: *Posterior* estimates for parameter $p$ using zero-deflated artificial samples (Scenario 4).

## 2.3 Using *complementary log-log* link function

Table 31: Empirical properties of the Bayesian estimators using zero-deflated artificial samples (Scenarios 1 and 2).

| $n$ | Parameter | Bias | MSE | $\sqrt{\frac{\text{MSE}}{\text{Var}}}$ | MAPE (%) |
|---|---|---|---|---|---|
| | | | Scenario 1 | | |
| 50 | $\beta_{10}$ | 0.029 | 0.293 | 1.001 | 43.753 |
| | $\beta_{11}$ | −0.053 | 0.701 | 1.002 | 66.609 |
| | $\beta_{20}$ | 0.003 | 0.145 | 1.000 | 58.741 |
| | $\beta_{21}$ | 0.026 | 0.453 | 1.001 | 104.497 |
| 100 | $\beta_{10}$ | 0.038 | 0.173 | 1.004 | 33.532 |
| | $\beta_{11}$ | −0.076 | 0.442 | 1.007 | 52.590 |
| | $\beta_{20}$ | 0.008 | 0.084 | 1.000 | 45.671 |
| | $\beta_{21}$ | −0.018 | 0.265 | 1.001 | 81.344 |
| 200 | $\beta_{10}$ | −0.008 | 0.083 | 1.000 | 23.138 |
| | $\beta_{11}$ | −0.005 | 0.227 | 1.000 | 38.595 |
| | $\beta_{20}$ | −0.015 | 0.037 | 1.003 | 30.755 |
| | $\beta_{21}$ | 0.024 | 0.128 | 1.002 | 57.261 |
| 500 | $\beta_{10}$ | 0.016 | 0.030 | 1.004 | 14.056 |
| | $\beta_{11}$ | −0.024 | 0.073 | 1.004 | 21.857 |
| | $\beta_{20}$ | 0.000 | 0.013 | 1.000 | 18.317 |
| | $\beta_{21}$ | 0.001 | 0.038 | 1.000 | 31.269 |
| | | | Scenario 2 | | |
| 50 | $\beta_{10}$ | 0.051 | 0.227 | 1.006 | 37.842 |
| | $\beta_{11}$ | −0.087 | 0.653 | 1.006 | 63.138 |
| | $\beta_{20}$ | 0.018 | 0.241 | 1.001 | 26.949 |
| | $\beta_{21}$ | −0.021 | 0.604 | 1.000 | 62.615 |
| 100 | $\beta_{10}$ | 0.046 | 0.148 | 1.007 | 30.337 |
| | $\beta_{11}$ | −0.075 | 0.421 | 1.007 | 51.438 |
| | $\beta_{20}$ | −0.003 | 0.141 | 1.000 | 19.373 |
| | $\beta_{21}$ | −0.005 | 0.344 | 1.000 | 45.974 |
| 200 | $\beta_{10}$ | 0.013 | 0.065 | 1.001 | 20.105 |
| | $\beta_{11}$ | −0.028 | 0.188 | 1.002 | 34.055 |
| | $\beta_{20}$ | 0.012 | 0.051 | 1.001 | 11.641 |
| | $\beta_{21}$ | −0.010 | 0.131 | 1.000 | 28.292 |
| 500 | $\beta_{10}$ | 0.008 | 0.028 | 1.001 | 13.446 |
| | $\beta_{11}$ | −0.013 | 0.072 | 1.001 | 21.606 |
| | $\beta_{20}$ | 0.003 | 0.026 | 1.000 | 8.472 |
| | $\beta_{21}$ | −0.012 | 0.059 | 1.001 | 19.093 |

Table 32: Empirical properties of the Bayesian estimators using zero-deflated artificial samples (Scenarios 3 and 4).

| $n$ | Parameter | Bias | MSE | $\sqrt{\frac{\text{MSE}}{\text{Var}}}$ | MAPE (%) |
|---|---|---|---|---|---|
| Scenario 3 | | | | | |
| 50 | $\beta_{10}$ | 0.046 | 0.333 | 1.003 | 45.611 |
| | $\beta_{11}$ | 0.370 | 1.070 | 1.071 | 55.202 |
| | $\beta_{20}$ | −0.008 | 0.149 | 1.000 | 59.638 |
| | $\beta_{21}$ | 0.000 | 0.478 | 1.000 | 105.774 |
| 100 | $\beta_{10}$ | −0.013 | 0.246 | 1.000 | 39.508 |
| | $\beta_{11}$ | 0.294 | 0.834 | 1.056 | 48.762 |
| | $\beta_{20}$ | −0.005 | 0.082 | 1.000 | 44.686 |
| | $\beta_{21}$ | −0.009 | 0.230 | 1.000 | 75.884 |
| 200 | $\beta_{10}$ | 0.007 | 0.100 | 1.000 | 25.257 |
| | $\beta_{11}$ | 0.129 | 0.482 | 1.018 | 36.353 |
| | $\beta_{20}$ | −0.006 | 0.029 | 1.001 | 26.834 |
| | $\beta_{21}$ | −0.004 | 0.111 | 1.000 | 53.146 |
| 500 | $\beta_{10}$ | −0.002 | 0.051 | 1.000 | 18.389 |
| | $\beta_{11}$ | 0.048 | 0.179 | 1.006 | 22.600 |
| | $\beta_{20}$ | 0.003 | 0.014 | 1.000 | 19.103 |
| | $\beta_{21}$ | −0.009 | 0.045 | 1.001 | 34.072 |
| Scenario 4 | | | | | |
| 50 | $\beta_{10}$ | −0.038 | 0.322 | 1.002 | 45.114 |
| | $\beta_{11}$ | 0.564 | 1.249 | 1.159 | 58.836 |
| | $\beta_{20}$ | −0.024 | 0.225 | 1.001 | 25.803 |
| | $\beta_{21}$ | −0.003 | 0.524 | 1.000 | 58.195 |
| 100 | $\beta_{10}$ | 0.013 | 0.228 | 1.000 | 38.213 |
| | $\beta_{11}$ | 0.188 | 0.898 | 1.020 | 49.778 |
| | $\beta_{20}$ | 0.010 | 0.140 | 1.000 | 19.337 |
| | $\beta_{21}$ | −0.042 | 0.334 | 1.003 | 45.964 |
| 200 | $\beta_{10}$ | −0.015 | 0.088 | 1.001 | 23.347 |
| | $\beta_{11}$ | 0.163 | 0.449 | 1.031 | 35.552 |
| | $\beta_{20}$ | −0.008 | 0.064 | 1.000 | 13.124 |
| | $\beta_{21}$ | 0.021 | 0.169 | 1.001 | 32.044 |
| 500 | $\beta_{10}$ | 0.007 | 0.041 | 1.000 | 16.224 |
| | $\beta_{11}$ | 0.031 | 0.170 | 1.003 | 21.934 |
| | $\beta_{20}$ | 0.011 | 0.027 | 1.002 | 8.345 |
| | $\beta_{21}$ | −0.013 | 0.058 | 1.002 | 18.718 |

Table 33: *Posterior* estimates for model parameters using zero-deflated artificial samples (Scenarios 1 and 2).

| $n$ | Parameter | Mean | Median | Std. Dev. | 95% HPDI Lower | 95% HPDI Upper |
|---|---|---|---|---|---|---|
| Scenario 1 | | | | | | |
| 50 | $\beta_{10}$ | −0.971 | −0.960 | 0.540 | −2.078 | 0.113 |
| | $\beta_{11}$ | 0.947 | 0.943 | 0.836 | −0.760 | 2.667 |
| | $\beta_{20}$ | 0.503 | 0.506 | 0.381 | −0.256 | 1.256 |
| | $\beta_{21}$ | 0.526 | 0.513 | 0.672 | −0.831 | 1.907 |
| 100 | $\beta_{10}$ | −0.962 | −0.956 | 0.414 | −1.805 | −0.126 |
| | $\beta_{11}$ | 0.924 | 0.922 | 0.661 | −0.418 | 2.270 |
| | $\beta_{20}$ | 0.508 | 0.511 | 0.290 | −0.051 | 1.062 |
| | $\beta_{21}$ | 0.482 | 0.478 | 0.514 | −0.499 | 1.474 |
| 200 | $\beta_{10}$ | −1.008 | −1.006 | 0.287 | −1.550 | −0.470 |
| | $\beta_{11}$ | 0.995 | 0.995 | 0.476 | 0.095 | 1.897 |
| | $\beta_{20}$ | 0.485 | 0.486 | 0.191 | 0.139 | 0.829 |
| | $\beta_{21}$ | 0.524 | 0.522 | 0.358 | −0.132 | 1.181 |
| 500 | $\beta_{10}$ | −0.984 | −0.983 | 0.174 | −1.338 | −0.633 |
| | $\beta_{11}$ | 0.976 | 0.975 | 0.270 | 0.424 | 1.528 |
| | $\beta_{20}$ | 0.500 | 0.500 | 0.113 | 0.277 | 0.722 |
| | $\beta_{21}$ | 0.501 | 0.500 | 0.194 | 0.110 | 0.892 |
| Scenario 2 | | | | | | |
| 50 | $\beta_{10}$ | −0.949 | −0.939 | 0.474 | −1.991 | 0.077 |
| | $\beta_{11}$ | 0.913 | 0.911 | 0.803 | −0.768 | 2.600 |
| | $\beta_{20}$ | 1.518 | 1.477 | 0.491 | 0.488 | 2.633 |
| | $\beta_{21}$ | −1.021 | −0.984 | 0.777 | −2.690 | 0.579 |
| 100 | $\beta_{10}$ | −0.954 | −0.948 | 0.382 | −1.750 | −0.168 |
| | $\beta_{11}$ | 0.925 | 0.924 | 0.644 | −0.384 | 2.233 |
| | $\beta_{20}$ | 1.497 | 1.480 | 0.376 | 0.756 | 2.270 |
| | $\beta_{21}$ | −1.005 | −0.986 | 0.586 | −2.210 | 0.168 |
| 200 | $\beta_{10}$ | −0.987 | −0.985 | 0.254 | −1.496 | −0.484 |
| | $\beta_{11}$ | 0.972 | 0.972 | 0.432 | 0.090 | 1.855 |
| | $\beta_{20}$ | 1.512 | 1.505 | 0.226 | 1.046 | 1.991 |
| | $\beta_{21}$ | −1.010 | −1.002 | 0.362 | −1.792 | −0.242 |
| 500 | $\beta_{10}$ | −0.992 | −0.991 | 0.169 | −1.325 | −0.661 |
| | $\beta_{11}$ | 0.987 | 0.987 | 0.268 | 0.447 | 1.525 |
| | $\beta_{20}$ | 1.503 | 1.500 | 0.162 | 1.206 | 1.804 |
| | $\beta_{21}$ | −1.012 | −1.009 | 0.243 | −1.478 | −0.552 |

Table 34: *Posterior* estimates for model parameters using zero-deflated artificial samples (Scenarios 3 and 4).

| $n$ | Parameter | Mean | Median | Std. Dev. | 95% HPDI Lower | 95% HPDI Upper |
|---|---|---|---|---|---|---|
| Scenario 3 | | | | | | |
| 50 | $\beta_{10}$ | −0.954 | −0.935 | 0.576 | −2.304 | 0.363 |
| | $\beta_{11}$ | −1.131 | −1.117 | 0.966 | −3.610 | 1.332 |
| | $\beta_{20}$ | 0.492 | 0.496 | 0.386 | −0.264 | 1.243 |
| | $\beta_{21}$ | 0.500 | 0.489 | 0.691 | −0.845 | 1.866 |
| 100 | $\beta_{10}$ | −1.013 | −1.001 | 0.496 | −2.073 | 0.029 |
| | $\beta_{11}$ | −1.206 | −1.200 | 0.864 | −3.170 | 0.750 |
| | $\beta_{20}$ | 0.495 | 0.498 | 0.286 | −0.063 | 1.047 |
| | $\beta_{21}$ | 0.491 | 0.488 | 0.479 | −0.488 | 1.476 |
| 200 | $\beta_{10}$ | −0.993 | −0.988 | 0.316 | −1.664 | −0.333 |
| | $\beta_{11}$ | −1.371 | −1.363 | 0.682 | −2.756 | 0.002 |
| | $\beta_{20}$ | 0.494 | 0.495 | 0.169 | 0.149 | 0.836 |
| | $\beta_{21}$ | 0.496 | 0.494 | 0.333 | −0.155 | 1.150 |
| 500 | $\beta_{10}$ | −1.002 | −1.000 | 0.226 | −1.440 | −0.568 |
| | $\beta_{11}$ | −1.452 | −1.450 | 0.421 | −2.304 | −0.602 |
| | $\beta_{20}$ | 0.503 | 0.504 | 0.120 | 0.280 | 0.725 |
| | $\beta_{21}$ | 0.491 | 0.491 | 0.212 | 0.101 | 0.881 |
| Scenario 4 | | | | | | |
| 50 | $\beta_{10}$ | −1.038 | −1.020 | 0.566 | −2.329 | 0.224 |
| | $\beta_{11}$ | −0.936 | −0.921 | 0.964 | −3.372 | 1.476 |
| | $\beta_{20}$ | 1.476 | 1.440 | 0.474 | 0.477 | 2.552 |
| | $\beta_{21}$ | −1.003 | −0.967 | 0.724 | −2.620 | 0.549 |
| 100 | $\beta_{10}$ | −0.987 | −0.977 | 0.477 | −1.984 | −0.011 |
| | $\beta_{11}$ | −1.312 | −1.303 | 0.929 | −3.247 | 0.616 |
| | $\beta_{20}$ | 1.510 | 1.493 | 0.375 | 0.766 | 2.287 |
| | $\beta_{21}$ | −1.042 | −1.021 | 0.576 | −2.251 | 0.134 |
| 200 | $\beta_{10}$ | −1.015 | −1.011 | 0.296 | −1.643 | −0.394 |
| | $\beta_{11}$ | −1.337 | −1.328 | 0.650 | −2.696 | 0.008 |
| | $\beta_{20}$ | 1.492 | 1.485 | 0.253 | 1.028 | 1.967 |
| | $\beta_{21}$ | −0.979 | −0.971 | 0.410 | −1.757 | −0.211 |
| 500 | $\beta_{10}$ | −0.993 | −0.992 | 0.203 | −1.403 | −0.589 |
| | $\beta_{11}$ | −1.469 | −1.466 | 0.411 | −2.304 | −0.635 |
| | $\beta_{20}$ | 1.511 | 1.508 | 0.163 | 1.212 | 1.813 |
| | $\beta_{21}$ | −1.013 | −1.010 | 0.241 | −1.479 | −0.551 |

Table 35: Coverage probabilities (%) of the HPDIs using zero-deflated artificial samples (Scenarios 1 and 2).

| $n$ | Parameter | BNCP | CP | ANCP | BNCP | CP | ANCP |
|---|---|---|---|---|---|---|---|
| | | | Scenario 1 | | | Scenario 2 | |
| 50 | $\beta_{10}$ | 3.40 | 95.80 | 0.80 | 3.40 | 95.80 | 0.80 |
| | $\beta_{11}$ | 1.20 | 96.60 | 2.20 | 1.00 | 96.00 | 3.00 |
| | $\beta_{20}$ | 1.60 | 96.80 | 1.60 | 0.00 | 97.40 | 2.60 |
| | $\beta_{21}$ | 0.80 | 97.40 | 1.80 | 2.00 | 98.00 | 0.00 |
| 100 | $\beta_{10}$ | 3.20 | 96.20 | 0.60 | 3.80 | 94.60 | 1.60 |
| | $\beta_{11}$ | 1.20 | 95.80 | 3.00 | 1.80 | 95.80 | 2.40 |
| | $\beta_{20}$ | 2.00 | 96.80 | 1.20 | 0.40 | 96.80 | 2.80 |
| | $\beta_{21}$ | 2.40 | 96.00 | 1.60 | 2.40 | 96.80 | 0.80 |
| 200 | $\beta_{10}$ | 3.00 | 93.80 | 3.20 | 2.60 | 95.20 | 2.20 |
| | $\beta_{11}$ | 2.20 | 95.00 | 2.80 | 2.00 | 96.00 | 2.00 |
| | $\beta_{20}$ | 3.20 | 93.80 | 3.00 | 0.60 | 96.80 | 2.60 |
| | $\beta_{21}$ | 3.40 | 94.40 | 2.20 | 2.20 | 97.40 | 0.40 |
| 500 | $\beta_{10}$ | 2.20 | 96.60 | 1.20 | 3.40 | 94.40 | 2.20 |
| | $\beta_{11}$ | 0.80 | 96.40 | 2.80 | 1.80 | 94.40 | 3.80 |
| | $\beta_{20}$ | 2.20 | 96.00 | 1.80 | 2.40 | 92.80 | 4.80 |
| | $\beta_{21}$ | 2.20 | 95.40 | 2.40 | 3.00 | 94.60 | 2.40 |

Table 36: Coverage probabilities (%) of the HPDIs using zero-deflated artificial samples (Scenarios 3 and 4).

| $n$ | Parameter | BNCP | CP | ANCP | BNCP | CP | ANCP |
|---|---|---|---|---|---|---|---|
| | | | Scenario 3 | | | Scenario 4 | |
| 50 | $\beta_{10}$ | 3.20 | 96.80 | 0.00 | 2.20 | 97.80 | 0.00 |
| | $\beta_{11}$ | 1.00 | 98.60 | 0.40 | 3.80 | 96.20 | 0.00 |
| | $\beta_{20}$ | 1.00 | 97.40 | 1.60 | 0.00 | 96.40 | 3.60 |
| | $\beta_{21}$ | 0.80 | 97.00 | 2.20 | 1.80 | 98.00 | 0.20 |
| 100 | $\beta_{10}$ | 2.60 | 96.60 | 0.80 | 3.00 | 96.20 | 0.80 |
| | $\beta_{11}$ | 2.80 | 96.80 | 0.40 | 4.00 | 95.40 | 0.60 |
| | $\beta_{20}$ | 2.00 | 95.80 | 2.20 | 0.20 | 96.80 | 3.00 |
| | $\beta_{21}$ | 1.60 | 96.40 | 2.00 | 1.40 | 98.20 | 0.40 |
| 200 | $\beta_{10}$ | 2.20 | 96.80 | 1.00 | 2.40 | 96.20 | 1.40 |
| | $\beta_{11}$ | 3.40 | 95.40 | 1.20 | 3.80 | 95.60 | 0.60 |
| | $\beta_{20}$ | 1.80 | 95.60 | 2.60 | 1.20 | 94.40 | 4.40 |
| | $\beta_{21}$ | 2.80 | 95.00 | 2.20 | 4.20 | 94.00 | 1.80 |
| 500 | $\beta_{10}$ | 1.60 | 96.20 | 2.20 | 3.00 | 95.20 | 1.80 |
| | $\beta_{11}$ | 3.00 | 96.00 | 1.00 | 3.00 | 96.00 | 1.00 |
| | $\beta_{20}$ | 2.40 | 94.00 | 3.60 | 2.80 | 94.80 | 2.40 |
| | $\beta_{21}$ | 2.20 | 95.00 | 2.80 | 2.00 | 95.60 | 2.40 |

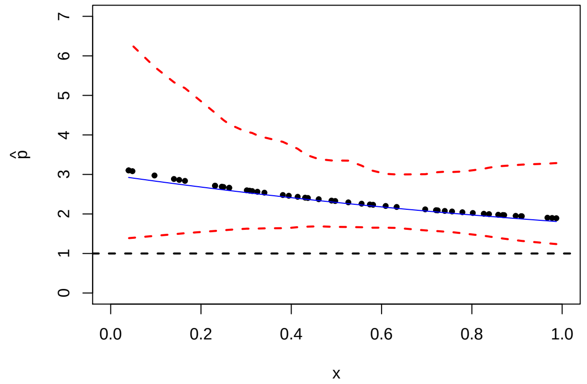


(a) $n = 50$

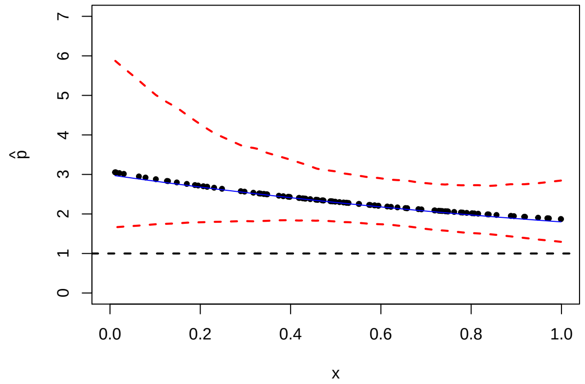


(b) $n = 100$

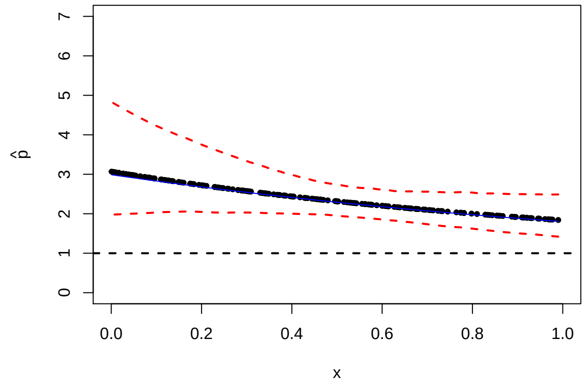


(c) $n = 200$

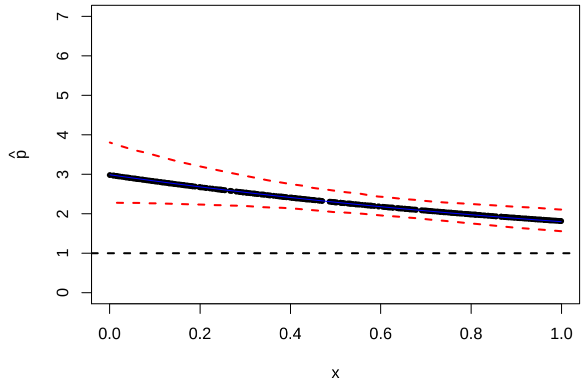


(d) $n = 500$

Figure 21: *Posterior* estimates for parameter $p$ using zero-deflated artificial samples (Scenario 1).

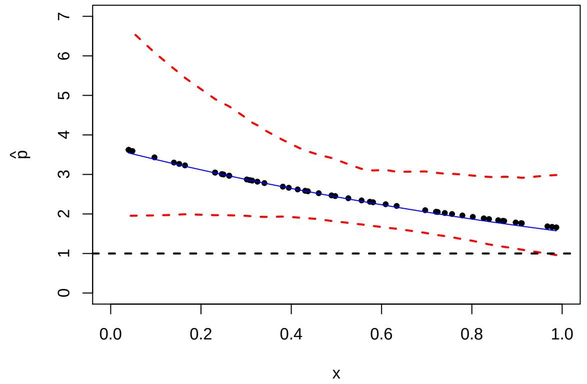


(a) $n = 50$

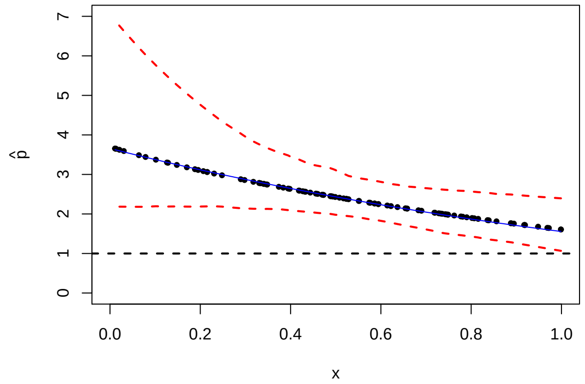


(b) $n = 100$

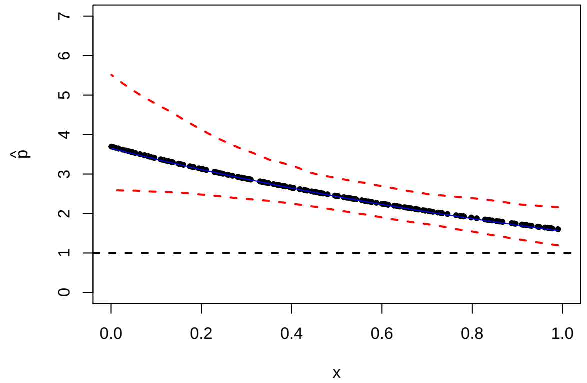


(c) $n = 200$

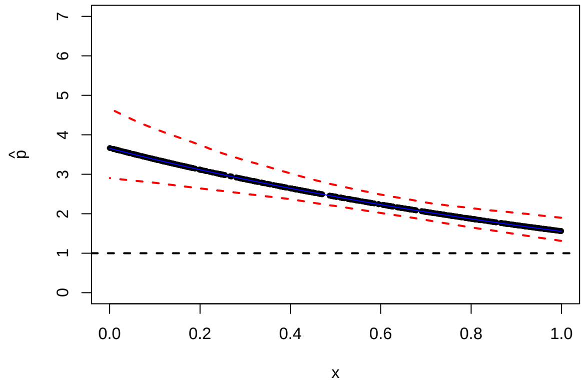


(d) $n = 500$

Figure 22: *Posterior* estimates for parameter $p$ using zero-deflated artificial samples (Scenario 2).

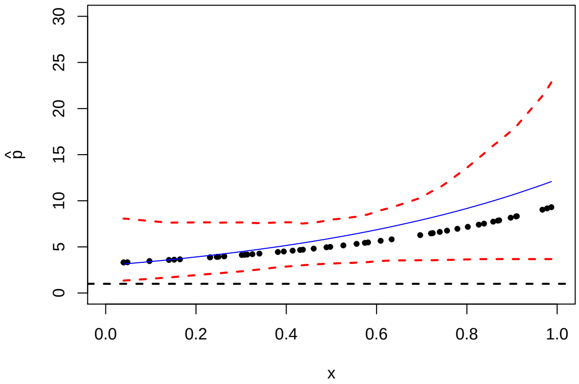


(a) $n = 50$

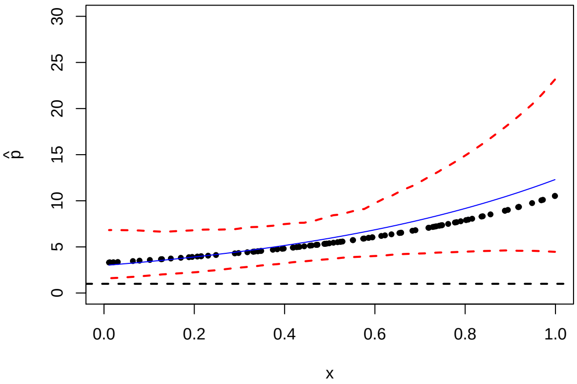


(b) $n = 100$

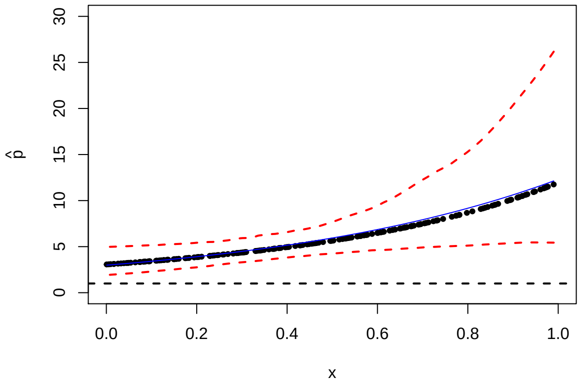


(c) $n = 200$

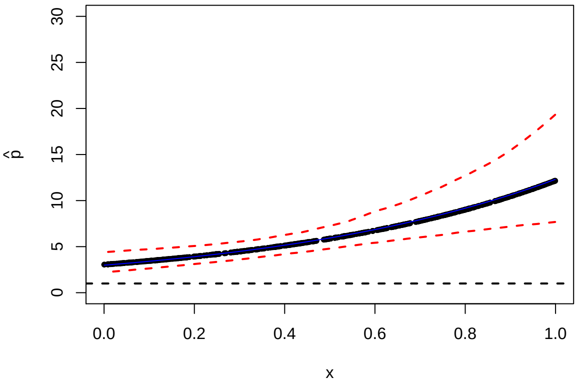


(d) $n = 500$

Figure 23: *Posterior* estimates for parameter $p$ using zero-deflated artificial samples (Scenario 3).

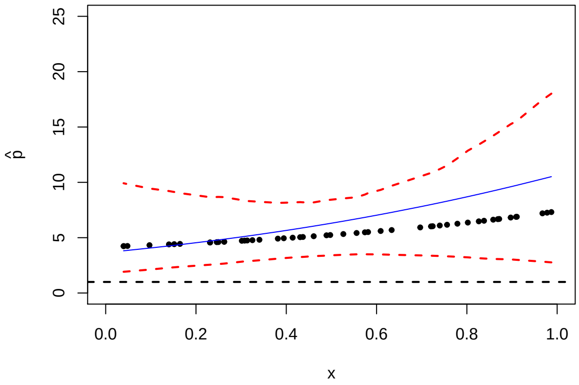


(a) $n = 50$

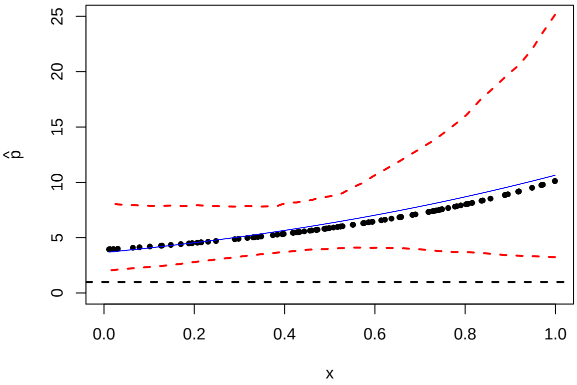


(b) $n = 100$

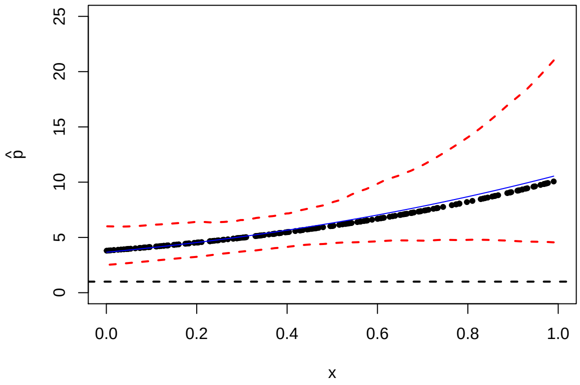


(c) $n = 200$

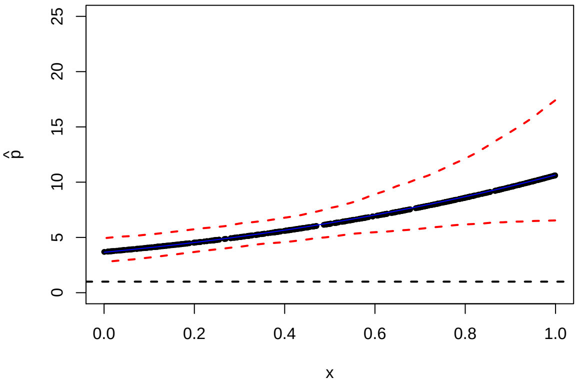


(d) $n = 500$

Figure 24: *Posterior* estimates for parameter $p$ using zero-deflated artificial samples (Scenario 4).
